# Pediatric efficacy and safety in common cold treated with herbal medicine (PEACH): a systematic review and meta-analysis

**DOI:** 10.3389/fphar.2025.1703997

**Published:** 2026-01-14

**Authors:** Soo-Dam Kim, Su Won Lee, Seong-Cheon Woo, Dong-Hyeon Kim, Lee Keon Jun, Jong-Hee Kim, Dong-Yeol Yang, Byung-Gab Kang, Yang-Chun Park, Boram Lee, Yee Ran Lyu

**Affiliations:** 1 KM Science Research Division, Korea Institute of Oriental Medicine, Daejeon, Republic of Korea; 2 Jaseng Spine and Joint Research Institute, Jaseng Medical Foundation, Seoul, Republic of Korea; 3 Department of Internal Medicine, College of Korean Medicine, Daejeon University, Daejeon, Republic of Korea; 4 Department of Korean Internal Medicine, Korean Medicine Hospital, Pusan National University, Yangsan, Republic of Korea

**Keywords:** common cold, herbal medicine, Korean medicine, meta-analysis, pediatrics, systematic review

## Abstract

**Introduction:**

The common cold is one of the most frequent illnesses in children, yet effective and safe treatments remain limited. Concerns regarding the safety of conventional medications persist. Herbal medicine (HM) has long been used, but the quantity and certainty of evidence in children have not been systematically summarized.

**Methods:**

We conducted a systematic review and meta-analysis of randomized controlled trials (RCTs) following PRISMA guidelines (PROSPERO CRD42024610421). Nine databases were searched to October 2024. Eligible studies included pediatric patients (<19 years) treated with oral HM compared with conventional medicine, placebo, or no treatment. Risk of bias was assessed with RoB 2, and certainty of evidence was rated using GRADE.

**Results:**

Sixty-six RCTs involving 7,176 participants were included. HM significantly improved total effective rate compared with conventional medicine (RR = 1.16, 95% CI [1.12–1.21]), with combination therapy providing additional benefits (RR = 1.17, 95% CI [1.13–1.22]). HM shortened overall symptom improvement time (SMD = −1.01, 95% CI [–1.41, −0.60]) and reduced total symptom severity (SMD = −0.80, 95% CI [–1.22, −0.38]) compared with conventional medicine. Both HM monotherapy and combination therapy demonstrated a markedly lower incidence of adverse events compared with conventional medicine (RR = 0.28, 95% CI [0.20, 0.40] and RR = 0.53, 95% CI [0.32, 0.88], respectively), with reported events being mild and mainly gastrointestinal. The certainty of evidence was predominantly moderate according to the GRADE assessment, increasing confidence in these findings.

**Conclusion:**

HM appears effective and safe for pediatric common cold, providing faster recovery and fewer adverse events. These findings, supported by moderate-certainty evidence, justify consideration of HM in pediatric care, although further high-quality, multicenter RCTs are needed.

**Systematic Review Registeration:**

https://www.crd.york.ac.uk/prospero/display_record.php?RecordID=610421, identifier CRD42024610421.

## 1 Introduction

The common cold is one of the most frequent illnesses in children, accounting a large proportion of outpatient visits and school absences worldwide (Turyasiima et al., 2024; Zhang et al., 2022; Worrall, 2011). Pediatric patients experience an average of 6–10 colds annually, particularly during early childhood (Canadian Paediatric Society Infectious Diseases and Immunization Committee, 2005, Worrall, 2011). Although the condition is generally self-limiting, its high incidence imposes substantial social and economic burdens, including parental work loss and inappropriate antibiotic use (Keshvari et al., 2023; Heikkinen and Järvinen, 2003; Cotton et al., 2008). In South Korea, the common cold remains one of the top diagnoses in pediatric primary care, leading to frequent medical consultations and medication prescriptions (Shin et al., 2015). While typically mild, pediatric common cold is characterized by frequent recurrence associated with immature immune function and continuous exposure to diverse respiratory viruses, and may lead to complications such as otitis media and bronchitis, intensifying parental concern (Heikkinen and Järvinen, 2003). As a result, caregivers often seek timely and effective interventions to reduce symptom duration and prevent worsening, emphasizing the need for safe and accessible treatment strategies in pediatric care (Pachter et al., 1998).

Despite ongoing efforts, there is currently no curative treatment for the common cold. Standard medical management primarily focuses on symptomatic relief through antipyretics, antihistamines, decongestants, and cough suppressants (DeGeorge et al., 2019; Nahas and Balla, 2011). However, these treatments often exhibit limited effectiveness and raise considerable safety concerns, particularly for younger pediatric patients (Diantini et al., 2024). Notably, the use of decongestants and cough medications is discouraged in children under 6 years because of potential adverse events, including excessive sedation, cardiac arrhythmia, and seizures (Summerlin and Eiland, 2025). Furthermore, frequent antibiotic prescriptions for viral respiratory infections contribute significantly to the global crisis of antimicrobial resistance, further complicating pediatric care (Llor and Bjerrum, 2014; Picca et al., 2023). These limitations highlight the urgent need for safer and more effective alternatives suitable for children. Consequently, integrative approaches, including complementary and traditional medicines, are increasingly gaining attention as potential adjunctive or alternative therapies in pediatric respiratory management (Lucas et al., 2019).

Herbal medicine (HM), a core modality of Korean Medicine (KM), has been widely used for pediatric respiratory illnesses, including the common cold. Herbal formulas such as Soshiho-tang (Xiaochaihu-tang in Chinese, Shosaiko-to in Japanese) and Eunkyosan (Yinqiao-san in Chinese, Gingyosan in Japanese) are commonly prescribed based on symptom differentiation and have been reported to relieve cough, nasal congestion, and fever with favorable safety profiles (Kim et al., 2023; Lee et al., 2020; Tran et al., 2023). Clinical studies suggest that these herbal formulas exert anti-inflammatory, antiviral, and immunomodulatory effects, including the regulation of cytokines such as IL-6 and TNF-α (Tran et al., 2023; Choi et al., 2021). Randomized controlled trials (RCTs) in patients with the common cold have demonstrated significant symptom reduction and shortened illness duration compared to standard care or placebo (Kim et al., 2023). Such findings provide both clinical and mechanistic support for the use of HM in respiratory care. Nevertheless, systematic reviews focusing specifically on children remain scarce. Most existing reviews include mixed-age populations or lack consistent outcome reporting, underscoring the need for pediatric-focused meta-analyses to inform evidence-based recommendations (Wu et al., 2007; Li et al., 2015; Chen et al., 2014).

The current Korean Medicine Clinical Practice Guidelines (KMCPGs) for the common cold, which were developed in 2021, were largely derived from adult populations and contain limited pediatric-specific recommendations (The Society of Internal Korean Medicine, 2021). Only a few herbal formulas (e.g., Soshiho-tang, Eunkyosan, and Socheongryong-tang) are mentioned for children, and most recommendations are based on low-level evidence or expert consensus, rather than robust clinical trials (The Society of Internal Korean Medicine, 2021). This imbalance reflects the lack of pediatric-specific data and creates challenges for clinicians seeking evidence-based decisions in pediatric practice.

To address this gap, the present study was conducted as part of an advanced development project to refine the KMCPGs for the common cold to overcome these limitations and enhance clinical applicability. The objective of this systematic review and meta-analysis was to evaluate the effect and safety of HM, focusing on pediatric patients with the common cold. The findings are expected to provide a scientific foundation for future updates of the KMCPGs and support the development of more reliable and standardized treatment strategies for pediatric respiratory care.

## 2 Methods

This systematic review and meta-analysis were conducted in accordance with a predefined protocol registered in the International Prospective Register of Systematic Reviews (PROSPERO: CRD42024610421). The reporting of this review follows the Preferred Reporting Items for Systematic Reviews and Meta-Analyses (PRISMA) 2020 guidelines (Page et al., 2021).

### 2.1 Definition of preparations

The interventions evaluated in this review were multiple oral traditional East Asian polyherbal preparations rather than a single standardized product. Full species names including authorities and family, as well as pharmacopeial drug names where available, were reported for all botanical drugs (Heinrich et al., 2022; Rivera et al., 2014). Taxonomic validation was conducted using the Plants of the World Online (Kew, https://powo.science.kew.org/) and Medicinal Plant Names Services (MPNS, https://mpns.kew.org) databases. The composition of each preparation, as reported in the original trials, is summarized in Supplementary Appendix 1, and missing compositional information is explicitly labelled as not reported (NR). Because this study is a systematic review, extraction methods, processing procedures, and manufacturing conditions were not investigated directly by the authors and are reported only as described in the original studies when available.

### 2.2 Criteria for inclusion and exclusion

RCTs with two or more arms were included according to the following criteria: (1) Studies involving participants under 19 years of age diagnosed with the common cold; (2) Studies using oral HM interventions based on traditional East Asian medicine theories. Studies using non-oral forms or herbal products not grounded in traditional theories (e.g., American ginseng) were excluded; (3) Studies comparing HM with conventional medicine (biomedicine), placebo, or no treatment. Studies involving HM combined with other therapies as treatment interventions were included if the other therapies were used equally in both the treatment and the control groups. Studies comparing only different forms of HM or other Korean medicine interventions were excluded; (4) Studies that assessed at least one of the following outcomes: total symptom severity, total symptom improvement time, severity or improvement time of individual symptoms (e.g., nasal congestion, rhinorrhea, cough, fever), quality of life (QoL), total effective rate (TER), or incidence of adverse events (AEs); (5) In cases of duplicate publications using the same dataset (e.g., journal and conference abstracts), the journal publication was included. If both were published in journals, the earlier one was selected; (6) Conference abstracts without sufficient outcome data for analysis were excluded; (7) There were no restrictions regarding the language of publication.

### 2.3 Literature searches

A comprehensive literature search was conducted across nine electronic databases to identify studies evaluating the efficacy and safety of traditional HM in patients with the common cold. The databases included PubMed, EMBASE, the Cochrane Central Register of Controlled Trials (CENTRAL), the China National Knowledge Infrastructure (CNKI), Wanfang Data, Citation Information by NII (CiNii), ScienceON, the Research Information Sharing Service (RISS), and the Oriental Medicine Advanced Searching Integrated System (OASIS). The search was conducted up to 14 October 2024. Key search terms included combinations of “Common Cold,” “Rhinovirus,” “Rhinitis,” “Respiratory Tract Infections,” “herbal medicine,” “traditional medicine,” “Korean medicine,” “Chinese medicine,” “Kampo medicine,” “plants,” and “randomized controlled trial.” The detailed search strategies used for each database are presented in Supplementary Table S1. The reference lists of relevant studies were reviewed to find any eligible studies. To include grey literature, conference proceedings and theses were also included. If the full text of a potentially eligible study was not accessible through the database, we attempted to obtain it by contacting institutional libraries or corresponding authors directly. Only studies for which the full text could be retrieved were included in the review.

### 2.4 Study selection

All search results were managed using EndNote version 21, and duplicate records were removed. Two independent reviewers (S.D.K. and B.L.) screened the titles and abstracts to exclude studies that did not meet the eligibility criteria. Full texts of the remaining studies were then assessed to determine final inclusion. Any disagreements between the two reviewers were resolved through discussion or by consulting a third reviewer (Y.R.L.).

### 2.5 Data extraction

Two independent reviewers (S.D.K. and B.L.) extracted the following data from each included study: title and year of publication, total number of participants, participant characteristics (e.g., age, sex, and duration of illness), details of the intervention and control groups, treatment dosage and duration, and outcome measures. Any discrepancies in the data extraction process were resolved through discussion or by consulting a third reviewer (Y.R.L.).

### 2.6 Risk of bias and quality assessment

The risk of bias in the included studies was assessed using the revised Cochrane risk-of-bias tool for randomized trials (RoB 2) developed by the Cochrane Collaboration (Sterne et al., 2019). Two independent reviewers evaluated each study across five domains:

1. Bias arising from the randomization process
2. Bias due to deviations from intended interventions
3. Bias due to missing outcome data
4. Bias in measurement of the outcome
5. Bias in selection of the reported result

Each domain was judged as “low risk,” “some concerns,” or “high risk” according to the RoB two criteria. The overall risk of bias was determined based on the results across domains. Any disagreements between reviewers were resolved through discussion or consultation with a third reviewer. Based on this assessment, the certainty of the evidence for each outcome was evaluated using the GRADE (Grading of Recommendations Assessment, Development and Evaluation) approach (Goldet and Howick, 2013). The quality of evidence from RCTs was initially rated as high, but was downgraded when limitations were identified in one or more domains, including risk of bias, inconsistency (heterogeneity), indirectness (differences in population, intervention, comparator, or outcome), imprecision (wide confidence intervals or small sample sizes), and potential publication bias.

### 2.7 Data analysis

Meta-analyses were conducted using RevMan Web in accordance with the Cochrane Handbook for Systematic Reviews of Interventions (version 6.5) (Sterne et al., 2019). For dichotomous outcomes, risk ratios (RRs) with 95% confidence intervals (CIs) were used. For continuous outcomes measured on the same scale, mean differences (MDs) with 95% CIs were used; when different scales were applied across studies, standardized mean differences (SMDs) were calculated. Statistical heterogeneity was assessed using the I^2^ statistic. Given the considerable clinical heterogeneity among the included studies, a random-effects model was applied. Publication bias was assessed visually using funnel plots when at least 10 studies were available for a given outcome. Sensitivity analyses were conducted by excluding studies with high risk of bias or methodological limitations (e.g., unusually large sample size or effect estimate) to evaluate the robustness of the results. Subgroup analyses were planned, when data were available, based on factors such as treatment duration and type of HM intervention to explore potential sources of heterogeneity. In addition, a frequency analysis of botanical drugs used in the included trials was performed to identify the most commonly prescribed botanical drugs and their relative distribution.

## 3 Results

### 3.1 Literature search

**FIGURE 1:**
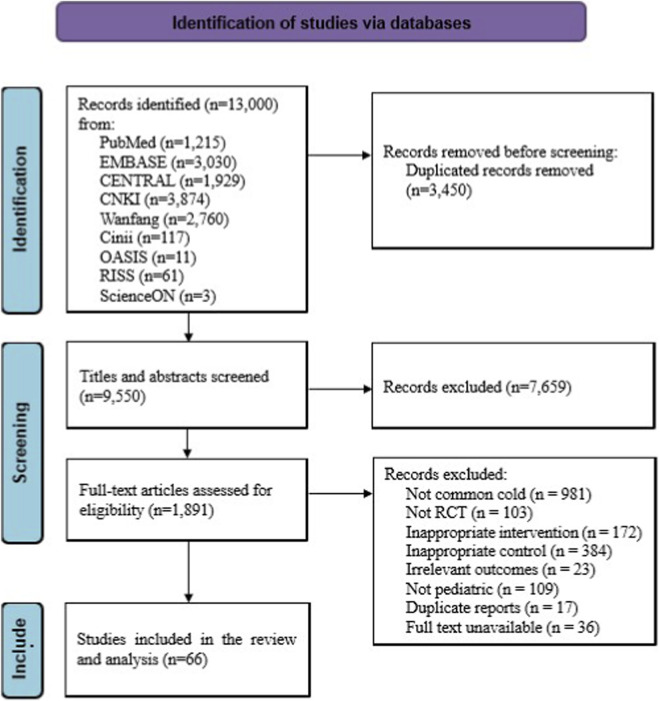
Flow chart of selection process. RCT, randomized controlled trial.

A total of 13,000 records were initially identified through database searches. After removing 3,450 duplicate records, 9,550 studies remained for title and abstract screening. Of these, 7,659 were excluded as irrelevant. The full texts of 1,891 articles were assessed for eligibility, and 1,825 were excluded for the following reasons: not related to the common cold (n = 981), not RCTs (n = 103), inappropriate intervention (n = 172), inappropriate control (n = 384), irrelevant outcome measures (n = 23), not conducted in pediatric populations (n = 109), duplicate reports (n = 17), and inaccessible full texts (n = 36). Ultimately, 66 studies were included in the meta-analysis. The study selection process is illustrated in the PRISMA flow diagram (Figure 1).

### 3.2 Study characteristics

This review included a total of 66 RCTs involving 7,176 participants. The participants ranged in age from 6 months and were primarily children under the age of 12. Regarding the duration of illness, most studies did not report the information (n = 28); however, the majority indicated that participants received treatment within 3 days of symptom onset. Of the included trials, 52 evaluated HM alone, while 14 assessed HM combined with conventional medicine compared to conventional medicine alone.

The most frequent HMs used in the included studies were Xiao Chai Hu Tang (22.1%), followed by Ganmao Qingre granules (7.4%), Xingfang Baidu San (7.4%), Yin Qiao San (4.4%), and Ge Gen Tang (4.4%). In the control groups, common comparators included ribavirin (oral or injectable), amoxicillin-based antibiotics, pediatric paracetamol (with or without chlorpheniramine maleate and artificial cow-bezoar), ibuprofen suspension, ketotifen, and conventional symptomatic treatments. Treatment duration varied across studies, ranging from 3 to 7 days in most trials, with a few extending to 2–4 weeks. The most commonly assessed outcome was the TER, followed by time to symptom resolution (e.g., fever, cough) and reports of AEs (Table 1). The detailed compositions of the herbal formulas used in the included studies are provided in Supplementary Appendix 1.

**TABLE 1:** Study characteristics included in the analysis.

Study ID	Disease duration	Age	Sample size	Intervention	Control	Duration	Outcomes measures	Adverse events
An (2012)	TG: 1–3 days CG: NR	TG: 6 months–12 years CG: 6 months–12 years	TG: 30 CG: 30	Jin Yu Chai Hu tang	Ribavirin + Roxithromycin + ibuprofen + antipyretic	7 days	1) TER 2) Adverse events	None
Bai and Hou (2019)	NR	TG: 6.5 ± 1.3 CG: 6.6 ± 1.2	TG: 75 CG: 75	Jinyinhua Lu + control	Xiaoerfeirekechuan oral liquid	14 days	1) Symptom improvement time (fever, cough) 2) TER 3) Adverse events	TG: Diarrhea (1 case), rash (1 case) CG: Diarrhea (3 case), rash (3 case), vomiting (2 case)
Cai (2020)	NR	TG: 7.3 ± 1.3 CG: 7.5 ± 1.2	TG: 70 CG: 70	Yunshi Ganmao Heji + Ganmao Jiedu granules	Pediatric paracetamol, artificial cow-bezoar and chlorphenamine maleate granules	7 days	1) Symptom improvement time (cough, sore throat, fever) 2) TER 3) Adverse events	NR
Cao (2018)	TG: 29.0 ± 2.4 h CG: 29.0 ± 2.8 h	TG: 6.0 ± 1.4 CG: 6.5 ± 1.2	TG: 36 CG: 36	Xiao Chai Hu tang	Ibuprofen, ketotifen, amoxicillin, Procaterol	6 days	TER	NR
Ceng (2012)	NR	TG: 3–13 CG: 3–14	TG: 60 CG: 54	Xiao Chai Hu tang (M)	Ibuprofen, ketotifen, amoxicillin, Procaterol	3 days	1) TER 2) Adverse events	None
Chen et al. (2015)	NR	TG: 4.46 ± 4.74 CG: 5.21 ± 3.54	TG: 30 CG: 30	Yin qiao san (M)	Ribavirin + Roxithromycin	3 days	1) Symptom improvement time (fever) 2) TER	NR
Cong (1998)	TG: 3–5 days CG: 3–5 days	TG: 7.6 CG: 7.5	TG: 40 CG: 30	Da Huang Ma Xing Gan Cao tang	Amoxicillin Dry Syrup, paediatric compound paracetamol and amantadine Hydrochloride granules, Metamizole tablets	1 week	TER	NR
Cui and Cui (2019)	2 ± 0.5 days	6 ± 0.2	TG: 98 CG: 98	Ge Gen tang granules	Ribavirin granules	3 days	1) Total symptom improvement time 2) TER 3) Adverse events	None
Dai et al. (2018)	TG: 1.7 ± 0.66 days CG: 1.56 ± 0.6 days	TG: 5.91 ± 2.10 CG: 5.86 ± 2.07	TG: 56 CG: 56	Fuganlin oral liquid	*Bacillus Subtilis* and *Enterococcus* Faecium granules	3–6 days	1) Total symptom severity 2) TER 3) Adverse events	TG: None CG: Constipation (1 case), Rash (1 case)
Dan (2021)	TG: 30.42 ± 1.36 h CG: 29.5 ± 2.48 h	TG: 4.66 ± 2.13 CG: 5.32 ± 1.24	TG: 21 CG: 21	Pudilan Xiaoyan oral liquid	Ribavirin granules	≥7 days	1) Symptom improvement time (cough, rhinorrhea, sore throat, fever) 2) TER	NR
Di (2012)	1–2 days	6months–12 years	TG: 30 CG: 30	Ganmao Qingre granules	Chlorpheniramine and Pseudoephedrine granules	5 days	1) TER 2) Adverse events	None
Du (2014)	2 days	4.2 ± 1.6	TG: 34 CG: 29	Xiao Chai Hu tang	Amoxicillin, amantadine, ketotifen tablets, ibuprofen suspension, Bromocriptine tablets	6 days	TER	NR
Duo (2017)	TG: 3–5 days CG: 2–5 days	TG: 1–12 years CG: 2–10 years	TG: 38 CG: 34	Qizhen Tangsan + Sanchen san	Chlorpheniramine and Pseudoephedrine granules	5 days	1) TER 2) Adverse events	None
Gu (2018)	NR	TG: 4.1 ± 0.8 CG: 4.2 ± 0.9	TG: 44 CG: 44	Xiao’er Chaigui Tuire granules	Chlorpheniramine and Pseudoephedrine granules	5 days	1) Symptom improvement time (cough, sore throat) 2) TER 3) Adverse events	TG: None CG: Nausea (1 case)
Guan and Qiu (2017)	1–2 days	TG: 4.0 ± 2.3 CG: 4.1 ± 2.3	TG: 55 CG: 55	Xiao’er Chaigui Tuire granules	Chlorpheniramine and Pseudoephedrine granules	5 days	1) Total symptom improvement time 2) TER 3) Adverse events	TG: None CG: Nausea (1 case)
Han (2018)	TG: 3.20 ± 0.15 days CG: 3.31 ± 0.45 days	TG: 7.55 ± 0.45 CG: 7.15 ± 0.40	TG: 25 CG: 25	Herbal medicine + control	Paracetamol + Pentoxyverine and ammonium Chloride Syrup	NR	1) TER 2) Adverse events	TG: Hypersomnia (1 case) CG: Hypersomnia (1 case); Nausea (1 case); vomiting (2 case); Dry mouth (1 case)
Hu and Lei (2020)	NR	TG: 6.53 ± 2.31 CG: 6.43 ± 2.19	TG: 43 CG: 43	Xingfang granules	Ribavirin	7 days	1) Symptom improvement time (cough, nasal congestion, rhinorrhea) 2) TER	NR
Hua and Chen (2017)	<72 hourss	8 months–11 years	TG: 50 CG: 48	Jian’er Qingjie oral liquid	Ribavirin	3 days	TER	NR
Ju (2012)	NR	TG: 3–7 years CG: 3–7 years	TG: 81 CG: 83	Wushicha granules OR Xiao’er Ganmao granules OR Xiao’er Ganmao Shu granules	Ribavirin OR Hu Tong Children’s cold Syrup	3 days	1) Total symptom severity 2) Symptom score (fever, chills, rhinorrhea, cough, headache, nasal congestion, sore throat) 3) Symptom improvement time (fever) 4) Adverse events	TG: Asthma (1 case), abnormal Complete blood count (1 case) CG: Urticaria (1case)
Li et al. (2014)	TG: 1.62 ± 0.49 CG: 2.02 ± 0.69	TG: 4.91 ± 0.95 CG: 4.77 ± 0.77	TG: 50 CG: 50	Tuire oral liquid	Ribavirin	6 days	TER	NR
Li (2016a)	NR	TG: 6.81 ± 2.62 CG: 6.78 ± 3.12	TG: 50 CG: 50	Xiao’er Dingchuan oral liquid	Paracetamol and chlorpheniramine maleate granules	15 days	TER	NR
Li (2016b)	0.58 ± 0.32 months	Total: 8.42 ± 3.13	TG: 37 CG: 37	Huangqi Diaoying tang + control	Tongkang tablets	4 weeks	1) TER 2) Adverse events	TG: Abdominal distension (1 case) CG: Abdominal pain (2 cases), constipation (1 case)
Li (2016a)	≤3 days	TG: 3.6 ± 1.2 CG: 3.5 ± 1.3	TG: 70 CG: 70	Xiao’er Chaigui Tuire granules + control	Pediatric paracetamol and amantadine granules	3 days	1) Symptom improvement time (rhinorrhea, sore throat) 2) TER	NR
Li (2016b)	TG: 1.4–3.3 days CG: 1.5–3.5 days	TG: 2–9 CG: 3–9	TG: 50 CG: 50	Yin qiao san (M)	Ribavirin granules	3 days	1) TER 2) Adverse events	TG: 2 cases CG: 5 cases
Li (2019)	3.21 ± 1.14 days	6.21 ± 1.25	TG: 40 CG: 40	Yunshi Ganmao Heji + Xiao’er Chaigui Tuire granules	Pediatric paracetamol and chlorpheniramine maleate granules	7 days	1) Symptom improvement time (cough, sore throat) 2) TER 3) Adverse events	TG: Loss of appetite (1 cases), Rash/Urticaria (1) CG: Loss of appetite (1), Rash/Urticaria (2), Fatigue/Nausea (7)
Li (2020)	TC: 3.4 ± 0.3 days GC: 3.9 ± 0.days	TC: 6.4 ± 0.5 GC: 6.2 ± 0.7	TG: 40 CG: 40	Ge Gen tang granules	Ribavirin granules	7 days	1) Total symptom improvement time 2) TER	NR
Li (2021)	NR	3–12 years	TG: 105 CG: 102	Fenghan: Xingfang Baidu san/Fengre: Yinqiao san/Shire: Sanren tang	Children’s acetaminophen, chlorpheniramine maleate granules	3 days	1) Total symptom severity 2) TER	NR
Liu (2016)	NR	TG: 12.31 ± 2.14 CG: 11.31 ± 1.53	TG: 60 CG: 60	Huoxiang Zhengqi capsule + control	Fluid therapy, antiviral treatment	7 days	TER	NR
Liu (2018)	1–2 days	TG: 4.5 ± 2.8 CG: 4.3 ± 2.7	TG: 80 CG: 80	Ganmao Qingre granules	Children’s aminophen Yellow granules	5 days	1) TER 2) Adverse events	NR
Liu (2018)	TG: 1.15 ± 0.20 days CG: 1.12 ± 0.21 days	TG: 4.66 ± 2.52 CG: 4.52 ± 2.50	TG: 60 CG: 60	Jinyinhua Lu + control	Children’s aminophen Yellow granules	3 days	1) Symptom improvement time (fever, nasal congestion, rhinorrhea, cough) 2) TER	NR
Lu et al. (2024)	TG 3.52 ± 1.33 days CG 3.56 ± 1.34 days	TG: 9.86 ± 4.12 CG: 9.74 ± 4.23	TG: 60 CG: 60	Self-formulated Ma Gui Chai Ge tang + control	Interferon α2b spray + Xiao’er Chaihu granules	1 week	1) Total symptom severity 2) TER 3) Adverse events	TG: Digestive issues (4 case), headache (1 case), dizziness (1 case), rash (1 case), liver/kidney issue (1 case), others (2 case) CG: 13.33% incidence (3 digestive issues, dizziness (2 case), rash (1 case), liver/kidney issue (1 case), other (1 case)
Luo (1996)	NR	TG: 2.57 (5 months–7 years) CG: 3.1 (3 months–7 years)	TG: 600 CG: 600	Kangbingdu Fang	Virusol or Penicillin	3 days	TER	NR
Mao (2016)	2.0 days	6.2 (1–12 years)	TG: 98 CG: 98	Ge Gen tang granules	Ribavirin	3 days	1) Total symptom severity 2) Total symptom improvement time 3) TER 4) Adverse events	None
Peng and wang (2016)	NR	4.3 ± 1.4	TG: 32 CG: 32	Ganmao Qingre granules	Children’s acetaminophen and chlorpheniramine granules	5 days	1) Total symptom severity 2) Symptom improvement time (fever, cough, rhinorrhea) 3) TER	NR
Qi (2016)	1–2days	TG: 4.7 ± 3.3 CG: 4.3 ± 3.2	TG: 75 CG: 75	Xiao’er Chaigui Tuire granules	Children’s acetaminophen and chlorpheniramine granules	3 days	1) Symptom score (fever) 2) TER 3) Adverse events	None
Qian (2012)	TG: 3.1 ± 0.8 days CG: 3.3 ± 0.days	TG: 4.1 ± 1.2 CG: 4.3 ± 1.0	TG: 38 CG: 38	Runfei Xiaoshi Huatan herbal medicine	Ribavirin	3 days	1) Symptom improvement time (fever) 2) TER 3) Adverse events	TG: 0 CG: Sweat (9 case); gastrointestinal trouble (11 case)
Shen (2013)	24 h	TG: 2–12 CG: 3–13	TG: 18 CG: 18	Xiao Chai Hu tang	Conventional biomedicine such as acyclovir, amantadine, amoxicillin, mefenamic acid oral solution, and procaterol	1 week	TER	NR
Shen and Wu (2021)	NR	TG: 2.8 ± 1.3 CG: 2.6 ± 1.2	TG: 36 CG: 35	Lianhua Qingwen granules + control	Ribavirin	7 days	1) Total symptom improvement time 2) Symptom improvement time (fever) 3) TER 4) Adverse events	TG: Diarrhea (1 case), vomiting (2 cases), rash (1 case), CG: Diarrhea (1 case), vomiting (3 cases), rash (1 case)
Shi et al. (2015)	NR	TG: 3–13 CG: 3–14	TG: 72 CG: 48	Xiao Chai Hu tang (M)	Amoxicillin, ketotifen tablets, procaterol tablets, ibuprofen	3 days	Adverse events	TG: The condition becomes more serious (4 cases) CG: Vomiting (8 cases)
Shiet al. (2015)	NR	TG: 3 ± 1.4 CG: 3 ± 1.6	TG: 75 CG: 75	Xiao’er Jiebiao oral liquid	Cefaclor granules	7 days	1) Total symptom improvement time 2) Symptom improvement time (fever) 3) TER	NR
Shi (2021)	NR	TG: 6.61 ± 1.83 CG: 7.18 ± 2.55	TG: 30 CG: 30	Xiao Chai Hu tang (M)	Ribavirin	7 days	1) Symptom improvement time (cough, fever, nasal congestion, rhinorrhea) 2) TER 3) Adverse events	TG: none CG: Anemia (2 cases), fatigue (1 case), loss of appetite (4 cases)
Song 2017	NR	TG: 13.5 ± 7.8 (5–20) CG: 12.9 ± 8.3 (5–19)	TG: 38 CG: 38	Huoxiang Zhengqi capsule + control	Conventional biomedicine (fluid rehydration, antiviral drugs, metoclopramide)	14 days	TER Adverse events	TG: Mental fatigue and listlessness (2 cases) CG: Mental fatigue and listlessness (3 cases), persistent vomiting (2 cases), severe headache (2 cases), neck stiffness (1 case)
Wang (2011)	24 h	2–12 years	TG: 30 CG: 30	Xiao’er Ganmao granules	Ribavirin	3 days	1) Total symptom severity 2) Symptom score (fever, sore throat, nasal congestion, rhinorrhea, cough) 3) Symptom improvement time (fever) 4) TER 5) Adverse events	NR
Wang and Dong (2017)	TG: 1.83 ± 0.64 days CG: 1.76 ± 0.59 days	TG: 4.26 ± 1.66 CG: 4.43 ± 1.61	TG: 30 CG: 30	Ganmao Qingre granules	Pediatric paracetamol, atificial cow-bezoar and chlorphenamine maleate granules	5 days	1) Total symptom severity 2) Symptom improvement time (fever, nasal congestion and rhinorrhea, cough)	NR
Wen (2017)	NR	TG: 3.5 ± 1.5 CG: 3.2 ± 1.2	TG: 40 CG: 40	Xiao Chai Hu tang (M)	Amoxicillin-clavulanate potassium dispersible tablets, ibuprofen suspension, ketotifen tablets, procaterol tablets	3 days	1) Symptom improvement time (fever, cough) 2) TER	NR
Wu (2010)	24 h	2–12 years	TG: 32 CG: 28	Xiao’er Ganmao granules	Ribavirin granules	3 days	1) Total symptom severity 2) Symptom improvement time (fever) 3) TER 4) Adverse events	None
Wu (2023)	NR	TG: 5.69 ± 0.42 CG: 5.24 ± 0.36	TG: 60 CG: 60	Children’s Chiqiao Qingre granules	Ribavirin granules	3 days	TER	NR
Xu (2011)	NR	TG: 4.3 (8 months–7 years) CG: 4.1 (6 months–6.5 years)	TG: 39 CG: 20	Xiao Chai Hu tang (M)	Amoxicillin and clavulanate potassium dispersible tablets, ketotifen tablets, ibuprofen suspension, and procaterol tablets	3 days	TER	NR
Xu (2014)	NR	TG: 6.44 ± 2.16 CG: 6.45 ± 2.09	TG: 23 CG: 23	Xiao Chai Hu tang	Amoxicillin, acyclovir, mefenamic acid and pseudoephedrine oral solution, amantadine, and procaterol	6 days	TER	NR
Yan et al. (2021)	TG: 16.14 ± 5.04 h CG: 15.73 ± 4.93 h	TG: 8.21 ± 1.98 CG: 8.14 ± 2.06	TG: 60 CG: 60	Chaiyin oral liquid	Ribavirin granules	3 days	1) Symptom improvement time (fever, cough, nasal congestion and rhinorrhea, chills) 2) TER 3) Adverse events	TG: Leukopenia (1 case), nausea and vomiting (1 case) CG: Leukopenia (2 cases), nausea and vomiting (1 case), abdominal pain (1 case)
Yang (2009)	TG: 15.0 ± 8.0 h CG: 16.0 ± 6.0 h	TG: 25 ± 2 months CG: 26 ± 3 months	TG: 40 CG: 40	Xingfang Qingre san	Oral or intramuscular administration of ribavirin	3 days	1) Symptom improvement time (fever) 2) TER 3) Adverse events	TG: GI reactions (3 cases), sweating (4 cases) CG: GI reactions (10 cases), sweating (11 cases)
Yang (2010)	NR	TG: 3–13 CG: 3–14	TG: 30 CG: 27	Xiao Chai Hu tang (M)	Amoxicillin clavulanate potassium dispersible tablets, ketotifen tablets, ibuprofen suspension, and procaterol tablets	6 days	1) TER 2) Adverse events	TG: Worsening (1 case) CG: Vomiting (2 cases)
Yang et al. (2015)	TG: 1–2 days CG: 1–2 days	TG: 6 (7 months–12 years) CG: 6 (7 months–12 years)	TG: 37 CG: 37	Ganmao Qingre granules	Pediatric paracetamol, atificial cow-bezoar and chlorphenamine maleate granules	6 days	TER	NR
Yang (2016)	<24 h	6 months–5 years	TG: 50 CG: 50	Xiao’er Ganmao granules	Pediatric paracetamol, atificial cow-bezoar and chlorphenamine maleate granules	5 days	1) Total symptom improvement time 2) Symptom improvement time (fever, nasal congestion and rhinorrhea, sore throat) 3) TER	NR
Yang (2017)	NR	TG: 5.0 ± 1.8 CG: 4.5 ± 1.6	TG: 36 CG: 36	Xiaoer Feire Kechuan oral liquid + control	Ambroxol oral solution	5 days	1) Total symptom severity 2) TER 3) Adverse events	None
Yang et al. (2023)	3.1 ± 0.3 days	4.43 ± 0.7	TG: 34 CG: 34	Shang Feng san (M)	Naphazoline hydrochloride nasal drops	7 days	1) Symptom score (nasal congestion, rhinorrhea, sneezing) 2) TER 3) Adverse events	None
Ye (2016)	NR	TG: 5.2 ± 3.16 CG: 5.80 ± 3.34	TG: 55 CG: 55	Lanqin oral liquid	Rimantadine hydrochloride tablets, and pseudoephedrine hydrochloride, chlorphenamine maleate and dextromethorphan hydrobromide solution	3 days	1) Total symptom severity 2) Symptom improvement time (fever) 3) TER	NR
Zhang et al. (2013)	TG: 1–3 days CG: 1–3 days	TG: 6 months–12 years CG: 6 months–12 years	TG: 30 CG: 30	Xingfang Baidu san (M)	Ribavirin granules	7 days	TER	NR
Zhang (2017)	2 days (1–3 days)	NR	TG: 30 CG: 30	Weisu granules + control	Paracetamol, caffein, atificial, cow-bezoar and chlorphenamine maleate capsules	3 days	TER	NR
Zhang (2018a)	NR	TG: 7.42 ± 2.73 CG: 7.56 ± 2.98	TG: 50 CG: 50	Xiao Chai Hu tang (M)	Ribavirin tablets	1 week	1) Symptom improvement time (cough, rhinorrhea, nasal congestion, fever) 2) Adverse events	TG: none CG: Anemia (1 case), fatigue (2 cases), loss of appetite (3 cases)
Zhang (2018b)	NR	TG: 5.23 ± 3.14 CG: 5.35 ± 3.09	TG: 80 CG: 80	Xingfang Qingre san	Oral or intramuscular administration of ribavirin	3 days	1) Symptom improvement time (fever) 2) TER 3) Adverse events	TG: GI reactions (5 cases), sweating (7 cases) CG: GI reactions (23 cases), sweating (24 cases)
Zhang and Qiu (2022)	TG: 27.58 ± 4.3 h CG: 28.66 ± 5.24 h	TG: 7.34 ± 2.76 CG: 7.45 ± 2.87	TG: 20 CG: 18	Xiao Chai Hu tang + control	Ibuprofen suspension, ketotifen, amoxicillin, procaterol tablets	6 days	1) Total symptom improvement time 2) Symptom improvement time (cough, rhinorrhea, nasal congestion, fever) 3) TER	NR
Zhou (2016)	TG: 1.81 ± 0.6 days CG: 1.80 ± 0.62 days	TG: 5.6 ± 1.4 CG: 5.5 ± 1.6	TG: 64 CG: 64	Chimonanthus leaf granules + control	Ribavirin Injection	7 days	1) Symptom improvement time (fever, cough, sore throat, rhinorrhea and nasal congestion) 2) TER 3) Adverse events	TG: Anemia (1 case), Rash (1 case), diarrhea (2 case) CG: Anemia (4 case), Rash (3 case), diarrhea (4 case), other (1 case)
Zhu (2018)	NR	5.9 ± 3.8	TG: 40 CG: 40	Yinlai tang	Routine biomedicine treatment	6 days	TER	NR
Zhu (2021)	NR	TG: 5.56 ± 1.37 CG: 5.75 ± 1.23	TG: 60 CG: 60	Xiao Chai Hu tang	Amoxicillin, acyclovir, amantadine, Percarbazine	5 days	TER	NR
Zhu and Wang (2023)	TG: 3.10 ± 0.10 days CG: 3.08 ± 0.12 days	TG: 5.33 ± 0.14 CG: 5.14 ± 0.98	TG: 50 CG: 50	Xiao Chai Hu granules + control	Children’s acetaminophen and Pseudoephedrine Dispersible tablets	7 days	1) Symptom improvement time (cough, rhinorrhea, nasal congestion, fever) 2) TER 3) Adverse events	TG: Headache (1 case), nausea (1 case), dry mouth (1 case) CG: Nausea (1 case), body aches (1 case), dry mouth (2 case)

TG, treatment group; CG, control group; TER, total effective rate; M, modified; NR, not reported.

### 3.3 Risk of bias of included studies

All studies were rated as “some concerns” for bias arising from the randomization process due to insufficient information on allocation concealment. Bias due to deviations from intended interventions was also rated as “some concerns” because participants and personnel were aware of the interventions and no information on deviations was reported. Bias due to missing outcome data was judged as ‘low risk,’ while bias in selection of the reported result was rated as “some concerns” owing to the absence of registered protocols. Bias in measurement of the outcome was rated as “some concerns” for TER, total symptom severity, and individual symptom severity, but “low risk” for total symptom improvement time, individual symptom improvement time, and adverse events. Overall, all studies were judged as having “some concerns” of bias (Supplementary Figure S1).

### 3.4 Outcomes

#### 3.4.1 Total symptom severity

**FIGURE 2:**
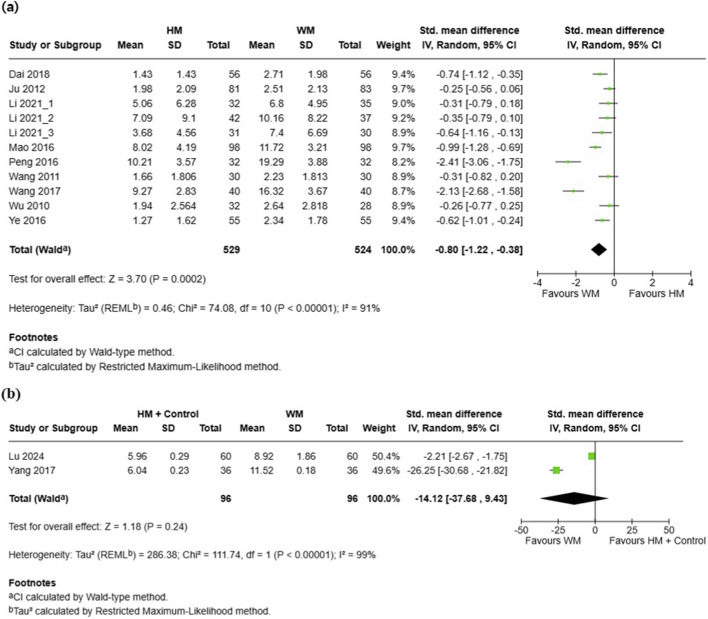
Meta-analysis of total symptom severity in pediatric common cold: **(a)** HM vs. Conventional medicine; **(b)** HM + Conventional medicine vs. Conventional medicine. HM, herbal medicine; WM, Western medicine; SD, standard deviation; SMD, standardized mean difference; CI, confidence interval.

A total of 11 RCTs involving 1,053 participants reported total symptom severity (Dai et al., 2018; Ju, 2012; Li, 2021; Mao, 2016; Peng and Wang, 2016; Wang, 2011; Wang and Dong, 2017; Wu, 2010; Ye, 2016). Meta-analysis demonstrated that the HM group had significantly lower symptom severity scores compared with the conventional medicine group (SMD = −0.80, 95% CI [–1.22, −0.38], p = 0.0002, I^2^ = 91%) (Figure 2a). The funnel plot showed noticeable asymmetry, suggesting a potential publication bias (Supplementary Figure S2a). Additionally, 2 RCTs involving 192 participants compared combination therapy with conventional medicine alone (Lu et al., 2024; Yang, 2017). The pooled results showed no significant difference in symptom severity scores between the HM plus control group and the conventional medicine group (SMD = −14.12, 95% CI [–37.68, 9.43], p = 0.24, I^2^ = 99%) (Figure 2b).

Subgroup analyses based on treatment duration demonstrated variability in both effect size and heterogeneity between the HM and conventional medicine groups. Significant benefits of HM were observed for 3 days of treatment (8 RCTs, n = 909; SMD = −0.52, 95% CI [–0.72, −0.32], I^2^ = 51%, p < 0.00001) and 5 days of treatment (2 RCTs, n = 144; SMD = −2.25, 95% CI [–2.67, −1.82], I^2^ = 0%, p < 0.00001). The test for subgroup differences was significant (Chi^2^ = 52.81, df = 1, p < 0.00001, I^2^ = 98.1%), indicating that treatment duration may contribute to heterogeneity (Supplementary Figure S3).

#### 3.4.2 Total symptom improvement time

**FIGURE 3:**
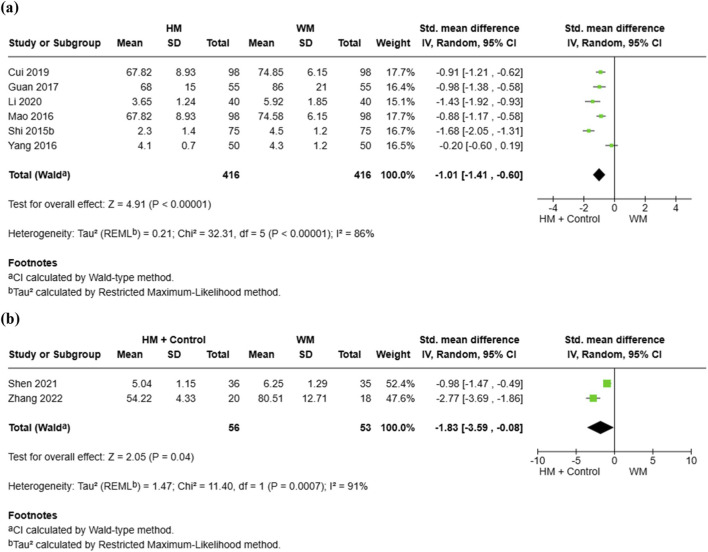
Meta-analysis of total symptom improvement time in pediatric common cold: **(a)** HM vs. Conventional medicine; **(b)** HM + Conventional medicine vs. Conventional medicine alone. HM, herbal medicine; WM, Western medicine; SD, standard deviation; SMD, standardized mean difference; CI, confidence interval.

A total of six RCTs involving 832 participants reported the time required for overall symptom improvement (Cui and Cui, 2019; Guan and Qiu, 2017; Li, 2020; Mao, 2016; Shi et al., 2015; Yang, 2016). Meta-analysis demonstrated that the HM group achieved a significantly shorter total symptom improvement time compared with the conventional medicine group (SMD = −1.01, 95% CI [–1.41, −0.60], p < 0.00001, I^2^ = 86%) (Figure 3a). Additionally, two RCTs with 109 participants compared combination therapy with conventional medicine alone (Shen and Wu, 2021; Zhang and Qin, 2022). The pooled results showed that the HM plus control treatment group achieved significantly shorter symptom improvement time than the conventional medicine group (SMD = −1.83, 95% CI [–3.59, −0.08], p = 0.04, I^2^ = 91%) (Figure 3b).

Subgroup analyses of the HM group and the conventional medicine group based on treatment duration revealed variability in effect size and heterogeneity. In studies with a treatment duration of 3 days, HM significantly shortened the total symptom improvement time compared with conventional medicine (SMD = −0.90, 95% CI [–1.10, −0.69], I^2^ = 0%, p < 0.00001). In contrast, studies with a duration of 5 days did not demonstrate a statistically significant difference (SMD = −0.59, 95% CI [–1.35, 0.17], I^2^ = 87%, p = 0.13). However, significant benefits were observed in studies with a treatment duration of 7 days (SMD = −1.59, 95% CI [–1.89, −1.29], I^2^ = 0%, p < 0.00001). The test for subgroup differences was significant (Chi^2^ = 15.71, df = 2, p = 0.0004, I^2^ = 87.3%), suggesting that treatment duration may contribute to heterogeneity (Supplementary Figure S4).

#### 3.4.3 Individual symptom severity

A total of four RCTs evaluated the effect of HM compared with conventional medicine on individual symptom severity. Two RCTs (n = 224) assessed cough severity, showing no significant difference between groups (SMD = 0.04, 95% CI [–0.28, 0.36], I^2^ = 23%, p = 0.81) (Ju, 2012; Wang, 2011). Three RCTs (n = 374) evaluated fever severity, with no statistical difference (SMD = −0.40, 95% CI [–1.27, 0.48], I^2^ = 94%, p = 0.37) (Wang, 2011; Ju, 2012; Qi, 2016). Three RCTs (n = 292) reported nasal congestion severity, but results showed no evidence of HM superiority (SMD = −1.53, 95% CI [–4.23, 1.16], I^2^ = 99%, p = 0.26) (Ju, 2012; Wang, 2011; Yang et al., 2023). Similarly, three RCTs (n = 292) assessed rhinorrhea severity, showing no significant benefit of HM compared with conventional medicine (SMD = −2.36, 95% CI [–6.36, 1.64], I^2^ = 99%, p = 0.25) (Yang et al., 2023; Ju, 2012; Wang, 2011). Two RCTs (n = 224) investigated sore throat severity, and no significant difference was observed (SMD = −0.41, 95% CI [–1.26, 0.45], I^2^ = 87%, p = 0.35) (Wang, 2011; Ju, 2012) (Table 2).

**TABLE 2:** Individual Symptom Severity (HM vs. Conventional medicine).

Symptoms	Studies	Participants	Std. mean difference IV, random, 95% CI	P value	Study ID references
Cough	2	224	SMD 0.04, 95% CI [-0.28, 0.36], I^2^ = 23%	P = 0.81	Ju 2012, Wang 2011
Fever	3	374	SMD -0.40, 95% CI [-1.27, 0.48], I^2^ = 94%	P = 0.37	Ju 2012, Qi 2016, Wang 2011
Nasal congestion	3	292	SMD -1.53, 95% CI [-4.23, 1.16], I^2^ = 99%	P = 0.26	Ju 2012, Wang 2011, Yang et al., 2023
Rhinorrhea	3	292	SMD -2.36, 95% CI [-6.36, 1.64], I^2^ = 99%	P = 0.25	Ju 2012, Wang 2011, Yang et al., 2023
Sore throat	2	224	SMD -0.41, 95% CI [-1.26, 0.45], I^2^ = 87%	P = 0.35	Ju 2012, Wang 2011

HM, herbal medicine; SMD, standardized mean difference; CI, confidence interval; IV, inverse variance.

#### 3.4.4 Individual symptom improvement time

A total of 11 RCTs involving 1,020 participants reported cough improvement time, showing that the HM group achieved significantly shorter duration compared with the conventional medicine group (SMD = −1.93, 95% CI [–2.86, −1.00], I^2^ = 98%, p < 0.00001) (Cai, 2020; Dan, 2021; Gu, 2018; Hu and Lei, 2020; Li, 2019; Peng and Wang, 2016; Shi, 2021; Wang and Dong, 2017; Wen, 2017; Yan et al., 2021; Zhang, 2018b). Eighteen RCTs involving 1,706 participants assessed fever improvement time, demonstrating a significantly shorter duration in the HM group (SMD = −1.46, 95% CI [–2.56, −0.36], I^2^ = 99%, p = 0.01) (Cai, 2020; Chen et al., 2015; Dan, 2021; Ju, 2012; Peng and Wang, 2016; Qian, 2012; Shi et al., 2015; Shi, 2021; Wang, 2011; Wang and Dong, 2017; Wen, 2017; Wu, 2010; Yan et al., 2021; Yang, 2009; Yang, 2016; Ye, 2016; Zhang, 2018b; Zhang, 2018a). Six RCTs with 546 participants evaluated nasal congestion improvement time, showing a significant reduction in the HM group (SMD = −2.67, 95% CI [–5.31, −0.04], I^2^ = 99%, p = 0.05) (Hu and Lei, 2020; Shi, 2021; Wang and Dong, 2017; Yan et al., 2021; Yang, 2016; Zhang, 2018b). Eight RCTs with 652 participants reported rhinorrhea improvement time, and the HM group showed significantly shorter duration (SMD = −1.94, 95% CI [–3.26, −0.61], I^2^ = 98%, p = 0.004) (Dan, 2021; Hu and Lei, 2020; Peng and Wang, 2016; Shi, 2021; Wang and Dong, 2017; Yan et al., 2021; Yang, 2016; Zhang, 2018b). Five RCTs involving 530 participants analyzed sore throat improvement time, but there was no statistically significant difference between groups (SMD = −0.65, 95% CI [–1.70, 0.40], I^2^ = 97%, p = 0.22) (Cai, 2020; Dan, 2021; Gu, 2018; Li, 2019; Yang, 2016) (Table 3).

**TABLE 3:** Individual Symptom Improvement Time (HM vs. Conventional medicine).

Symptoms	Studies	Participants	Std. mean difference IV, random, 95% CI	P value	Study ID references
Cough	11	1,020	SMD -1.93, 95% CI [-2.86, −1.00], I^2^ = 98%	^***^P < 0.00001	Cai 2020, Dan 2021, Gu 2018, Hu and Lei, 2020, Li 2019, Peng and Wang, 2016, Shi 2021, Wang and Dong, 2017, Wen 2017, Yan et al., 2021, Zhang 2018a
Fever	18	1,706	SMD -1.46, 95% CI [-2.56, −0.36], I^2^ = 99%	^**^P = 0.01	Cai 2020, Chen et al., 2015, Dan 2021, Ju 2012, Peng and Wang, 2016, Qian 2012, Shi, 2015, Shi 2021, Wang 2011, Wang and Dong, 2017, Wen 2017, Wu 2010, Yan et al., 2021, Yang 2009, Yang 2016, Ye 2016, Zhang 2018a, Zhang 2018b
Nasal congestion	6	546	SMD -2.67, 95% CI [-5.31, −0.04], I^2^ = 99%	^*^P = 0.05	Hu and Lei, 2020, Shi 2021, Wang and Dong, 2017, Yan et al., 2021, Yang 2016, Zhang 2018a
Rhinorrhea	8	652	SMD -1.94, 95% CI [-3.26, −0.61], I^2^ = 98%	^**^P = 0.004	Dan 2021, Hu and Lei, 2020, Peng and Wang, 2016, Shi 2021, Wang and Dong, 2017, Yan et al., 2021, Yang 2016, Zhang 2018a
Sore throat	5	530	SMD -0.65, 95% CI [-1.70, 0.40], I^2^ = 97%	P < 0.22	Cai 2020, Dan 2021, Gu 2018, Li 2019, Yang 2016

HM, herbal medicine; SMD, standardized mean difference; CI, confidence interval; IV, inverse variance. *P < 0.05, **P < 0.01, ***P < 0.001.

A total of seven RCTs compared the time required for individual symptom improvement between the combination group and the conventional medicine group. Five RCTs (n = 536) reported cough improvement time, and meta-analysis showed that combination treatment significantly shortened cough recovery compared with conventional medicine (SMD = −3.32, 95% CI [–5.88, −0.76], I^2^ = 99%, p = 0.01) (Bai and Hou, 2019; Liu and Lin, 2019; Zhang and Qin, 2022; Zhou, 2016; Zhu and Wang, 2023). Six RCTs (n = 604) assessed fever improvement time, and combination group showed significantly superior effects (SMD = −4.14, 95% CI [–6.86, −1.43], I^2^ = 99%, p = 0.003) (Bai and Hou, 2019; Liu and Lin, 2019; Shen and Wu, 2021; Zhang and Qin, 2022; Zhou, 2016; Zhu and Wang, 2023). Four RCTs (n = 386) reported nasal congestion improvement, and results favored combination treatment (SMD = −3.93, 95% CI [–7.40, −0.46], I^2^ = 99%, p = 0.03) (Liu and Lin, 2019; Zhang and Qin, 2022; Zhou, 2016; Zhu and Wang, 2023). Five RCTs (n = 526) examined rhinorrhea recovery, showing significant benefit in the combination group (SMD = −3.60, 95% CI [–6.86, −0.33], I^2^ = 100%, p = 0.03) (Zhu and Wang, 2023; Li, 2016b; Liu and Lin, 2019; Zhang and Qin, 2022; Zhou, 2016). Two RCTs (n = 268) investigated sore throat, indicating a significant effect of combination treatment (SMD = −1.02, 95% CI [–1.32, −0.72], I^2^ = 25%, p < 0.00001) (Li, 2016b; Zhou, 2016) (Table 4).

**TABLE 4:** Individual Symptom Improvement Time (HM + Control vs. Conventional medicine).

Symptoms	Studies	Participants	Std. mean difference IV, random, 95% CI	P value	Study ID references
Cough	5	536	SMD -3.32, 95% CI [-5.88, −0.76], I^2^ = 99%	^**^P = 0.01	Bai and Hou, 2019, Liu and Lin, 2019, Zhang and Qin, 2022, Zhou 2016, Zhu and Wang, 2023
Fever	6	604	SMD -4.14, 95% CI [-6.86, −1.43], I^2^ = 99%	^**^P = 0.003	Bai and Hou, 2019, Liu and Lin, 2019, Shen and Wu, 2021, Zhang and Qin, 2022, Zhou 2016, Zhu and Wang, 2023
Nasal congestion	4	386	SMD -3.93, 95% CI [-7.40, −0.46], I^2^ = 99%	^*^P = 0.03	Liu and Lin, 2019, Zhang and Qin, 2022, Zhou 2016, Zhu and Wang, 2023
Rhinorrhea	5	526	SMD -3.60, 95% CI [-6.86, −0.33], I^2^ = 100%	^*^P = 0.03	Li and Qian, 2016, Liu and Lin, 2019, Zhang and Qin, 2022, Zhou 2016, Zhu and Wang, 2023
Sore throat	2	268	SMD -1.02, 95% CI [-1.32, 0.72], I^2^ = 25%	^***^P < 0.00001	Li and Qian, 2016, Zhou 2016

HM, herbal medicine; SMD, standardized mean difference; CI, confidence interval; IV, inverse variance. *P < 0.05, **P < 0.01, ***P < 0.001.

#### 3.4.5 Total effective rate

**FIGURE 4:**
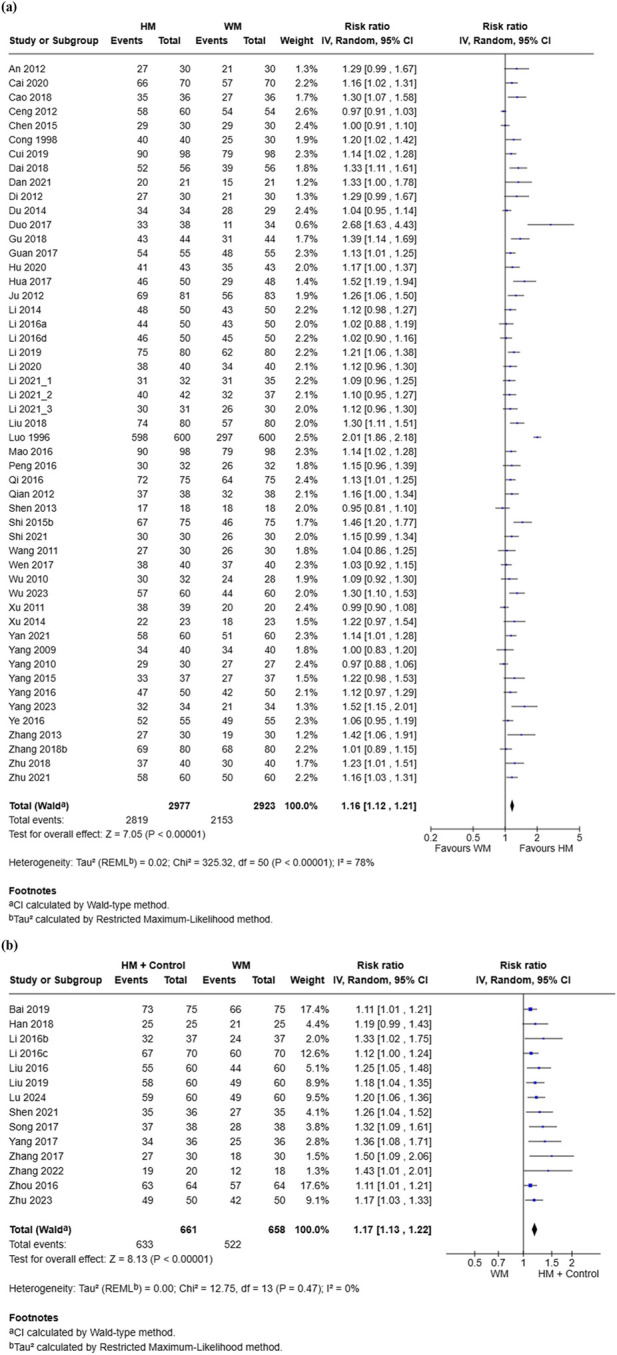
Meta-analysis of total effective rate in pediatric common cold: **(a)** HM vs. Conventional medicine; **(b)** HM + Conventional medicine vs. Conventional medicine alone. HM, herbal medicine; WM, Western medicine; SD, standard deviation; CI, confidence interval.

A total of 51 RCTs involving 5,900 participants included in the analysis comparing the TER between the HM group and the conventional medicine group. Overall, the HM group demonstrated a significantly higher TER (RR = 1.16, 95% CI [1.12, 1.21], p < 0.00001) than the conventional medicine group, with high initial heterogeneity (I^2^ = 78%) (An, 2012; Cai, 2020; Cao, 2018; Ceng, 2012; Chen et al., 2015; Cong, 1998; Cui and Cui, 2019; Dai et al., 2018; Dan, 2021; Di, 2012; Du, 2014; Duo, 2017; Gu, 2018; Guan and Qiu, 2017; Hu and Lei, 2020; Hua and Chen, 2017; Ju, 2012; Li, 2019; Li et al., 2016; Li, 2020; Li, 2016a; Li et al., 2014; Liu, 2018; Luo, 1996; Mao, 2016; Peng and Wang, 2016; Qi, 2016; Qian, 2012; Shen, 2013; Shi, 2021; Shi et al., 2015; Wang, 2011; Wen, 2017; Wu, 2023; Wu, 2010; Xu, 2011; Xu, 2014; Yan et al., 2021; Yang, 2009; Yang, 2010; Yang et al., 2015; Yang et al., 2023; Yang, 2016; Ye, 2016; Zhang et al., 2013; Zhang, 2018a; Zhu, 2021; Zhu, 2018; Li, 2021) (Figure 4a). Sensitivity analysis excluding one study (Luo, 1996), which showed an extreme effect size and unusually large sample size, reduced heterogeneity to a moderate level (I^2^ = 55%) while maintaining statistical significance, indicating that the robustness of the pooled result was not materially affected by this exclusion. The funnel plot suggested possible publication bias (Supplementary Figure S2b). Additionally, 14 RCTs involving 1,319 participants assessed the effect of combining HM with control treatment (Bai and Hou, 2019; Han, 2018; Li and Qian, 2016; Li, 2016b; Liu and Lin, 2019; Liu, 2016; Lu et al., 2024; Shen and Wu, 2021; Song, 2017; Yang, 2017; Zhang, 2017; Zhang and Qin, 2022; Zhou, 2016; Zhu and Wang, 2023). The pooled results indicated a significant benefit of the combination over conventional medicine alone (RR = 1.17, 95% CI [1.13, 1.22], p < 0.00001), with low heterogeneity (I^2^ = 0%, p = 0.47) (Figure 4b). Evidence of publication bias was also indicated by the funnel plot (Supplementary Figure S2c).

Subgroup analyses of TER based on intervention type demonstrated variability in both effect size and heterogeneity. Significant benefits of HM were observed for Chinese polyherbal preparation (CCPP) (RR = 1.18, 95% CI [1.13, 1.22], I^2^ = 25%), Xingfang Baidu San (RR = 1.09, 95% CI [1.01, 1.17], I^2^ = 8%), other HM (RR = 1.19, 95% CI [1.10, 1.29], I^2^ = 0%), Ge Gen Tang (RR = 1.13, 95% CI [1.06, 1.22], I^2^ = 0%), and mixed prescriptions (RR = 2.68, 95% CI [1.63, 4.43]) (p < 0.05). In contrast, Xiao Chai Hu Tang (RR = 1.05, 95% CI [0.99, 1.11], I^2^ = 60%) and Yin Qiao San (RR = 1.03, 95% CI [0.96, 1.10], I^2^ = 0%) did not show statistically significant differences (p > 0.05). The test for subgroup differences was significant (Chi^2^ = 32.63, df = 6, p < 0.00001, I^2^ = 81.6%), suggesting that the type of HM may contribute to heterogeneity (Supplementary Figure S3).

#### 3.4.6 Adverse events

**FIGURE 5:**
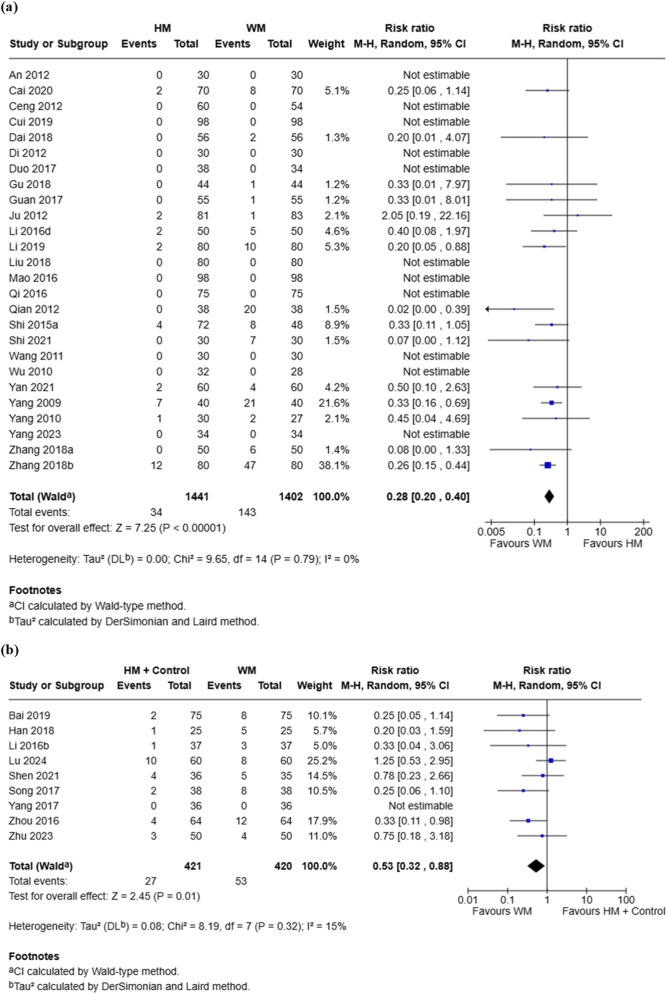
Meta-analysis of adverse events in pediatric common cold: **(a)** HM vs. Conventional medicine; **(b)** HM + Conventional medicine vs. Conventional medicine alone. HM, herbal medicine; WM, Western medicine; SD, standard deviation; CI, confidence interval.

A total of 26 RCTs involving 2,843 participants compared the incidence of AEs between the HM group and the conventional medicine group (An, 2012; Cai, 2020; Ceng, 2012; Cui and Cui, 2019; Dai et al., 2018; Di, 2012; Duo, 2017; Gu, 2018; Guan and Qiu, 2017; Ju, 2012; Li, 2019; Li, 2016a; Liu, 2018; Mao, 2016; Qi, 2016; Qian, 2012; Shi, 2021; Shi, 2015; Wang, 2011; Wu, 2010; Yan et al., 2021; Yang, 2009; Yang, 2010; Yang et al., 2023; Zhang, 2018a; Zhang, 2018b). Meta-analysis showed a significantly lower incidence of AEs in the HM group compared with the conventional medicine group (RR = 0.28, 95% CI [0.20, 0.40], I^2^ = 0%, p < 0.00001) (Figure 5a). Reported AEs in the HM group were mainly gastrointestinal symptoms such as abdominal distension, diarrhea, nausea, vomiting, indigestion, abdominal pain, and constipation (Table 1). Funnel plot analysis indicated a symmetrical distribution, suggesting a low risk of publication bias (Supplementary Figure S2d).

Eight RCTs involving 841 participants compared the incidence of AEs between the combination group and the conventional medicine group (Bai and Hou, 2019; Han, 2018; Li and Qian, 2016; Lu et al., 2024; Shen and Wu, 2021, Song, 2017; Yang, 2017; Zhou, 2016; Zhu and Wang, 2023). Meta-analysis showed a significantly lower incidence of AEs in the combination group compared with the conventional medicine group (RR = 0.53, 95% CI [0.32, 0.88], I^2^ = 15%, p = 0.01) (Figure 5b). The most common AEs in the combination group were gastrointestinal reactions, including diarrhea, vomiting, abdominal distension, abdominal pain, constipation, and nausea (Table 1).

### 3.5 Quality of evidence based on GRADE assessment

The certainty of evidence was predominantly moderate, with only one outcome rated as low. For comparisons between HM and conventional medicine, the certainty was moderate across all outcomes, including total effective rate, total symptom improvement time, total symptom severity, and adverse events. For combination therapy compared with conventional medicine, the certainty was moderate for total effective rate, total symptom improvement time, and adverse events, while low for total symptom severity. The detailed GRADE assessments are presented in Table 5.

**TABLE 5:** Summary of findings and certainty of evidence.

Comparison	Outcome and follow-up	Patients (studies), N	Relative effect (95% CI)	Absolute effects (95% CI)			Certainty
				[Comparison]	[Intervention]	Difference	
HM compared with conventional medicine	Total symptom severity	1053 (11 RCTs)	-	-	-	SMD -0.8 (−1.22 to −0.38)	⊕⊕⊕○ Moderate^a^
	Total symptom improvement time	832 (6 RCTs)	-	-	-	SMD -1.01 (−1.41 to −0.6)	⊕⊕⊕○ Moderate^a^
	Total effective rate	5900 (51 RCTs)	RR = 1.16 (1.12–1.21)	737 per 1,000	854 per 1,000 (825–891)	118 more per 1,000 (from 88 more to 155 more)	⊕⊕⊕○ Moderate^a^
	Adverse events	2843 (15 RCTs)	RR = 0.28 (0.20–0.40)	102 per 1,000	29 per 1,000 (20–41)	73 fewer per 1,000 (from 82 fewer to 61 fewer)	⊕⊕⊕○ Moderate^a^
Combination compared with conventional medicine	Total symptom severity	192 (2 RCTs)	-	-	-	SMD -14.12 (−37.68 to 9.43)	⊕⊕○○ Low^a^ ^,^ ^b^
	Total symptom improvement time	109 (2 RCTs)	-	-	-	SMD -1.83 (−3.59 to −0.08)	⊕⊕⊕○ Moderate^a^
	Total effective rate	1319 (14 RCTs)	RR = 1.17 (1.13–1.22)	793 per 1,000	928 per 1,000 (896–968)	135 more per 1,000 (from 103 more to 175 more)	⊕⊕⊕○ Moderate^a^
	Adverse events	841 (8 RCTs)	RR = 0.53 (0.32–0.88)	126 per 1,000	67 per 1,000 (40–111)	59 fewer per 1,000 (from 86 fewer to 15 fewer)	⊕⊕⊕○ Moderate^a^

HM, herbal medicine; CI, confidence interval; RR, risk ratio; SMD, standardised mean difference; RCT, randomized clinical trial.

^a^ Some concerns regarding the randomization process and blinding were identified.

^b^ Imprecision due to relatively small number of trials or wide confidence intervals.

### 3.6 Herbal frequency analysis

A total of 60 individual botanical drugs prescribed in at least two trials were identified across the included studies. The most frequently used botanical drugs were *Glycyrrhiza uralensis* (47.1%) and *Bupleurum chinense* (45.6%), followed by *Scutellaria baicalensis* (30.9%), *Schizonepeta tenuifolia* (25.0%), and *Forsythia suspensa* (23.5%). Detailed frequencies are presented in Supplementary Table S2.

## 4 Discussion

This systematic review and meta-analysis were conducted to comprehensively evaluate the efficacy and safety of HM for pediatric patients with the common cold. The findings demonstrated that HM alone was associated with significant improvements in TER, total symptom improvement time, and overall symptom severity compared with conventional medicine. Moreover, when HM was combined with conventional medicine, most outcomes showed superior effects compared with conventional medicine alone. Importantly, both HM monotherapy and combination therapy exhibited a lower incidence of AEs than conventional medicine, highlighting a favorable safety profile in children. These findings provide the first synthesis focused exclusively on pediatric populations, addressing critical evidence gap previously dominated by adult studies (Kim et al., 2021; Chen et al., 2014; Li et al., 2015), and highlight the clinical relevance of HM as a potential treatment option for the common cold in children.

In our meta-analysis, HM demonstrated a significantly higher TER compared with conventional medicine (RR = 1.16, p < 0.00001). HM combined with conventional medicine also improved TER compared with conventional care alone (RR = 1.17, p < 0.00001), highlighting the added value of integrative approaches in pediatric treatment (Anheyer et al., 2018). The heterogeneity observed across studies is likely explained by prescription diversity. Subgroup analysis indicated significant benefits for CCPP (p < 0.00001), Xingfang Baidu San (p = 0.03), other HM (p < 0.0001), Ge Gen Tang (p = 0.0005), and mixed prescriptions (p = 0.0001). These findings are consistent with previous reviews reporting superior response rates for herbal interventions in pediatric respiratory infections (Anheyer et al., 2018; Lee et al., 2023).

In contrast, Xiao Chai Hu Tang (p = 0.08) and Yinqiao-san (p = 0.43) did not demonstrate superiority. The limited effect of Xiao Chai Hu Tang may be due to most included studies did not adequately consider its traditional shaoyang-stage indications, such as alternating fever and chills, chest or hypochondriac discomfort, and poor appetite (Kwon et al., 2020). In addition, many of the analyzed prescriptions were modified versions of Xiao Chai Hu Tang with added or substituted botanical drugs rather than the classical formula, which may have further contributed to variability and reduced comparability across trials. Rigorous placebo-controlled trials that evaluate the original formula in children remain scarce (Tran et al., 2023). For Yinqiao-san, the therapeutic rationale is most evident in early wind-heat presentations, but its effectiveness diminishes as symptoms advance. Inconsistent timing of administration, broad inclusion criteria, and methodological variability across trials likely explain the mixed results observed in our analysis (Wong et al., 2012; Lee et al., 2020; Lam et al., 2009). The variability in effectiveness across formulas underscores the need for treatment guidelines to align specific herbal prescriptions with the distinct pathophysiological stages and symptom patterns of the common cold.

Our findings indicate that HM significantly reduced overall symptom severity compared with conventional medicine (SMD = −0.80, p = 0.0002), and also shortened overall symptom improvement time (SMD = −1.01), and showed an even larger effect when combined with conventional care (SMD = −1.83). These benefits were consistent across several key symptoms, including cough, fever, nasal congestion, and rhinorrhea, suggesting that HM may provide both greater symptomatic relief and faster recovery in pediatric patients. These improvements are clinically meaningful, particularly in light of parental concern regarding prolonged illness and the associated social and economic burdens, including school absenteeism and caregiver work loss (Allan and Arroll, 2014; Kardos and Malek, 2017). Faster symptom resolution may therefore help mitigate these burdens, which are particularly significant in early childhood when colds are frequent. Even modest improvements in recovery time could accumulate to produce meaningful long-term benefits for child wellbeing and family functioning.

Subgroup analyses further indicated that treatment duration was an important modifier of outcomes, with longer courses associated with greater benefits not only in symptom improvement but also in reductions in overall severity. Three-day regimens showed moderate effects on both symptom improvement (SMD = −0.90, 95% CI [–1.10, −0.69]) and severity reduction (SMD = −0.52, 95% CI [–0.72, −0.32]), whereas 7-day regimens demonstrated stronger benefits (SMD = −1.59, 95% CI [–1.89, −1.29] for improvement time; SMD = −2.25, 95% CI [–2.67, −1.82] for severity). Nevertheless, the presence of high heterogeneity and evidence of publication bias highlight methodological limitations, while individual symptom severity outcomes (cough, fever, nasal congestion, rhinorrhea, sore throat) did not show consistent advantages of HM over conventional medicine. These findings underscore the need for standardized pediatric outcome measures, such as a core outcome set, to improve comparability across future trials and to provide more reliable evidence for clinical practice (Lei et al., 2022; Seylanova et al., 2024).

Both HM monotherapy and combination therapy demonstrated a markedly lower incidence of AEs compared with conventional medicine (RR = 0.28 and RR = 0.53, respectively), with low heterogeneity (I^2^ = 0–15%) and symmetrical funnel plots supporting the robustness of the findings. Reported AEs were predominantly mild gastrointestinal symptoms, such as diarrhea, nausea, and abdominal discomfort, and no serious events were identified. These results are consistent with previous systematic reviews on herbal treatments for pediatric respiratory infections, which also reported favorable tolerability and no significant increase in adverse reactions compared with placebo (Anheyer et al., 2018). By contrast, conventional over-the-counter medications such as decongestants and cough suppressants have been linked to significant safety concerns in children, including oversedation, arrhythmias, and seizures (Diantini et al., 2024). Taken together, the evidence suggests that HM not only provides therapeutic benefits but also offers a safer profile for pediatric patients with the common cold, supporting its consideration in future clinical guidelines. Nevertheless, herb–drug interactions were not systematically evaluated in the included studies, and long-term safety outcomes were unavailable, which warrants cautious interpretation. This safety advantage is particularly important in pediatrics, where children are more vulnerable to adverse drug reactions due to immature metabolic and excretory systems, and where tolerability often determines caregiver acceptance and adherence.

The therapeutic effects of HM for pediatric common cold can be better understood by examining the bioactive metabolites of the most frequently prescribed botanical drugs and their known biological activities. Among these, *G. uralensis* is particularly notable because its major triterpenoid saponins and flavonoids, including glycyrrhizin and liquiritigenin, exhibit antiviral, anti-inflammatory, and immunomodulatory properties through inhibition of viral replication and suppression of pro-inflammatory cytokines such as IL-6 and TNF-α (Jiang et al., 2021). Likewise, *S. baicalensis* contains abundant flavones such as baicalin and wogonin, which exert broad-spectrum antiviral effects and modulate NF-κB and MAPK signaling pathways, thereby attenuating airway inflammation (Zhao et al., 2016). *Forsythia suspensa* is rich in phenylethanoid glycosides, including forsythiaside and phillyrin, which show antibacterial and antiviral activity as well as antioxidant and anti-inflammatory effects (Wang et al., 2018).

Other frequently used botanical drugs appear to complement these mechanisms. *Bupleurum chinense*, which contains saikosaponins, plays a prominent role in fever reduction and immune regulation via hypothalamic–pituitary–adrenal axis modulation and cytokine control (Ran et al., 2024). *Schizonepeta tenuifolia* and *Saposhnikovia divaricata* contain essential oils (e.g., pulegone, methyleugenol) and chromones such as prim-O-glucosylcimifugin, which contribute antipyretic, analgesic, and immunoregulatory actions, supporting symptomatic relief in febrile respiratory illnesses (Li et al., 2024). In addition, *Prunus armeniaca*, containing amygdalin, and *Platycodon grandiflorus*, rich in platycosides, exhibit antitussive, bronchodilatory, and expectorant effects, directly addressing predominant respiratory symptoms such as cough and airway obstruction (Ji et al., 2020).

Furthermore, supportive botanical drugs such as *Ziziphus jujuba* and *Mentha canadensis* enhance therapeutic outcomes by providing antioxidant, nutritional, and decongestant benefits through bioactive metabolites including jujubosides and menthol derivatives (Zhu et al., 2024; Saqib et al., 2022). Collectively, these findings suggest that the clinical utility of pediatric herbal prescriptions lies not merely in the number of botanical drugs employed, but in the rational combination of botanicals with complementary phytochemical profiles. By simultaneously targeting viral pathogens, inflammatory cascades, immune dysregulation, and symptom-specific pathways, these multi-metabolite prescriptions achieve a systems-level therapeutic effect that differs fundamentally from the single-target paradigm of conventional pharmacotherapy. This multi-layered pharmacological architecture provides a plausible mechanistic basis for the observed clinical effectiveness of herbal medicine in the management of pediatric common cold.

This study has several limitations that warrant consideration. First, the majority of the included trials were conducted in China, which may limit the generalizability of the findings to other cultural and healthcare contexts. Second, methodological concerns were present in many studies, including insufficient reporting of randomization processes, lack of blinding, and absence of trial registration, all contributing to a high or unclear risk of bias. These limitations increase the likelihood of performance and detection bias and reduce confidence in the internal validity of the pooled estimates. Third, there was considerable heterogeneity in treatment protocols and outcome measurement tools, and most studies did not employ validated symptom assessment instruments such as the Wisconsin Upper Respiratory Symptom Survey (WURSS), reducing comparability across studies. In addition, most trials had short follow-up periods and did not evaluate long-term outcomes such as recurrence or delayed adverse events. Furthermore, mechanistic studies linking pharmacological pathways to clinical endpoints in pediatric populations remain scarce. Collectively, these methodological weaknesses preclude firm conclusions regarding treatment efficacy, and the current findings should be interpreted as suggestive rather than confirmatory.

Despite these limitations, this review has several notable strengths. It is the first to synthesize evidence exclusively in children with the common cold, quantitatively evaluate both efficacy and safety outcomes, and incorporate subgroup and mechanistic analyses that provide insights into clinical benefits and pharmacological plausibility. In addition, most outcomes were supported by moderate-certainty evidence according to the GRADE assessment, which further reinforces the robustness and clinical relevance of our findings. Future research should prioritize large-scale, multicenter randomized trials with rigorous methodology, standardized pediatric outcome measures, and extended follow-up to strengthen the evidence base. In particular, validated patient-reported outcome instruments such as the WURSS should be adopted to improve comparability and reliability across studies. Moreover, biomarker-based outcomes should be integrated to elucidate mechanistic pathways and further validate clinical effects.

## 5 Conclusion

HM demonstrates clinically meaningful benefits in children with the common cold by improving symptoms, shortening recovery time, and reducing adverse events compared with conventional care. These findings, supported by moderate-certainty evidence from the GRADE assessment, underscore the potential role of HM as a safe and effective metabolite of integrative pediatric practice and support its inclusion in future clinical guidelines. Importantly, the observed clinical benefits are pharmacologically plausible, as key herbal metabolites exert complementary antiviral, anti-inflammatory, and immunomodulatory effects that align with the multifactorial pathophysiology of the common cold. Nevertheless, because most included trials were conducted in China and exhibited methodological heterogeneity, high-quality, multicenter RCTs using standardized and validated outcome measures are needed. Future research addressing these gaps will be essential to confirm these benefits and enhance their generalizability in pediatric care.

## Data Availability

Data supporting the findings of this study are available upon reasonable request to the corresponding authors.

## References

[B1] Allan G. M. Arroll B. (2014). Prevention and treatment of the common cold: making sense of the evidence. Cmaj 186, 190–199. 10.1503/cmaj.121442 24468694 PMC3928210

[B2] An C. X. (2012). Observation on the efficacy of jinyu chai Hu tang in 30 cases of pediatric wind-heat type common cold. J. Community Med. 10, 85.

[B3] Anheyer D. Cramer H. Lauche R. Saha F. J. Dobos G. (2018). Herbal medicine in children with respiratory tract infection: systematic review and meta-analysis. Acad. Pediatr. 18, 8–19. 10.1016/j.acap.2017.06.006 28610802

[B4] Bai C. Hou S. Q. (2019). Observation on the effect of xiaokuihualu brand honeysuckle dew combined with pediatric feire kechuan oral liquid in treating pediatric cold with cough. Famous Doctor, 10, 220–222.

[B5] Cai J. X. (2020). Clinical observation on yunshi ganmao mixture combined with ganmao jiedu granules in treating pediatric wind-cold type common cold. World Latest Med. Inf. Abstr. Electron. Contin. Ed. 20, 111–112.

[B6] Canadian Paediatric Society Infectious Diseases and Immunization Committee (2005). Colds in children. Paediatr. Child. Health 10, 493–495. 10.1093/pch/10.8.493 19668664 PMC2722603

[B7] Cao N. (2018). Clinical analysis of 72 cases of pediatric common cold treated with xiao chai Hu tang. Chin. Foreign Med. Care 37, 23–25.

[B8] Ceng L. B. (2012). Observation on the clinical efficacy of modified xiao chai Hu tang in treating pediatric common cold. Psychol. (Second Half) 223.

[B9] Chen W. Liu B. Wang L. Q. Ren J. Liu J. P. (2014). Chinese patent medicines for the treatment of the common cold: a systematic review of randomized clinical trials. BMC Complement. Altern. Med. 14, 273. 10.1186/1472-6882-14-273 25074623 PMC4129119

[B10] Chen L. X. Su H. Zhong X. H. (2015). Clinical comparative study of integrated Chinese and Western medicine regimens for pediatric wind-heat type common cold. Chin. Mod. Drug Appl. 9, 157–158.

[B11] Choi Y. Kim M. H. Jung D. H. Yang W. M. (2021). Anti-inflammatory effects of sosiho-tang, a traditional herbal formula, on acute lung injury in LPS-sensitized mice and -Raw 264.7 cells. Evid. Based Complement. Altern. Med. 2021, 6641689. 10.1155/2021/6641689 33628305 PMC7886507

[B12] Cong J. (1998). Treatment of 40 cases of pediatric common cold with Da Huang Ma Xing Gan Cao Tang – with 30 cases of Western medicine control observation. Zhejiang J. Traditional Chin. Med. 356.

[B13] Cotton M. Innes S. Jaspan H. Madide A. Rabie H. (2008). Management of upper respiratory tract infections in children. S Afr. Fam. Pract. 50, 6–12. 10.1080/20786204.2008.10873685 21603094 PMC3098742

[B14] Cui J. Cui Q. (2019). Observation on the efficacy of Ge gen tang granules in treating pediatric wind-cold type common cold. Electron. J. Integr. Traditional West. Cardiovasc. Med. 7, 168.

[B15] Dai W. Wan L. P. Wang S. J. (2018). Clinical observation of fugankeling oral liquid in treating pediatric gastrointestinal-type common cold. Shanghai Med. 39, 33–36.

[B16] Dan J. X. (2021). Study on the therapeutic effect of pudilan xiaoyan oral liquid in pediatric wind-heat type common cold. China Health Care Nutr. 31, 183.

[B17] Degeorge K. C. Ring D. J. Dalrymple S. N. (2019). Treatment of the common cold. Am. Fam. Physician 100, 281–289. 31478634

[B18] DI J. H. (2012). Observation on the efficacy of ganmao Qingre granules in treating 30 cases of pediatric wind-cold type common cold. Chin. J. Integr. Traditional West. Pediatr. 4, 277–278.

[B19] Diantini A. Alfaqeeh M. Permatasari L. I. Nurfitriani M. Durotulailah L. Wulandari W. (2024). Clinical toxicology of OTC cough and cold pediatric medications: a narrative review. Pediatr. Health Med. Ther. 15, 243–255. 10.2147/PHMT.S468314 39011322 PMC11249067

[B20] Du H. P. (2014). Clinical observation of 34 cases of pediatric common cold treated with xiao chai Hu tang. Chin. Ethn. Folk Med. 23, 50.

[B21] Duo J. Z. X. (2017). Clinical observation on 38 cases of pediatric common cold treated with Tibetan medicine sanchenshan combined with qizhentangsan. J. Chin. Ethn. Med. 23, 32–33.

[B22] Goldet G. Howick J. (2013). Understanding GRADE: an introduction. J. Evid. Based Med. 6, 50–54. 10.1111/jebm.12018 23557528

[B23] Gu Y. (2018). Clinical analysis of pediatric chai gui antipyretic granules in the treatment of pediatric wind-cold type common cold. Med. Inf. 31, 150–151.

[B24] Guan Z. H. Qiu W. J. (2017). Clinical observation of pediatric chai gui tui re granules in treating pediatric wind-cold type common cold. China Pract. Med. 12, 142–143.

[B25] Han D. R. (2018). Study on the impact of Chinese medicine intervention on the rational use of medication in children with the common cold. China Urban Rural Enterp. Hyg. 33, 107–108.

[B26] Heikkinen T. Järvinen A. (2003). The common cold. Lancet 361, 51–59. 10.1016/S0140-6736(03)12162-9 12517470 PMC7112468

[B27] Heinrich M. Jalil B. Abdel-Tawab M. Echeverria J. Kulić Ž. Mcgaw L. J. (2022). Best practice in the chemical characterisation of extracts used in pharmacological and toxicological research—The ConPhyMP—Guidelines12. Front. Pharmacol., 13–2022. 10.3389/fphar.2022.953205 PMC9514875 36176427

[B28] Hu H. Y. Lei W. (2020). Effect of jing Fang granules on serum inflammatory factor levels in children with wind-cold type common cold. Electron. J. Mod. Med. Health Res. 4, 125–126.

[B29] Hua P. J. Chen H. (2017). Clinical observation on Jian’er qingjie oral liquid combined with montmorillonite powder in treating gastrointestinal type common cold in children. Jiangsu Med. 43, 1434–1435.

[B30] Ji M. Y. Bo A. Yang M. Xu J. F. Jiang L. L. Zhou B. C. (2020). The pharmacological effects and health benefits of platycodon grandiflorus-A medicine food homology species. Foods 9, 142. 10.3390/foods9020142 32023858 PMC7073691

[B31] Jiang L. Akram W. Luo B. Hu S. Faruque M. O. Ahmad S. (2021). Metabolomic and pharmacologic insights of aerial and underground parts of Glycyrrhiza uralensis fisch. Ex DC. for maximum utilization of medicinal resources. Front. Pharmacol. 12, 658670. 10.3389/fphar.2021.658670 34140890 PMC8204184

[B32] Ju L. (2012). Evaluation study on syndrome-differentiated use of Chinese patent medicines in treating pediatric acute upper respiratory tract infection. Nanjing, China: Nanjing University of Chinese Medicine. Master’s Thesis.

[B33] Kardos P. Malek F. A. (2017). Common cold - an umbrella term for acute infections of nose, throat, larynx and bronchi. Pneumologie 71, 221–226. 10.1055/s-0042-116112 27912214 PMC7117077

[B34] Keshvari N. Yousefi N. Peiravian F. Sharif Z. (2023). Exploring health seeking behaviors for common cold management. Explor Res. Clin. Soc. Pharm. 11, 100301. 10.1016/j.rcsop.2023.100301 37533759 PMC10392600

[B35] Kim H. Choi J. Y. Hong M. Suh H. S. (2021). Traditional medicine for the treatment of common cold in Korean adults: a nationwide population-based study. Integr. Med. Res. 10, 100458. 10.1016/j.imr.2020.100458 32913704 PMC7473881

[B36] Kim K. I. Hong M. Park Y. C. Lee B. J. Kim K. Kang B. K. (2023). Effects of herbal medicines (Eunkyosan/Yin qiao san and Samsoeum/Shen su yin) for treating the common cold: a randomized, placebo-controlled, multicenter clinical trial. Integr. Med. Res. 12, 101005. 10.1016/j.imr.2023.101005 38033649 PMC10682673

[B37] Kwon S. Lee W. Jin C. Jang I. Jung W. S. Moon S. K. (2020). Could herbal medicine (soshihotang) be a new treatment option for COVID-19? a narrative review. Integr. Med. Res. 9, 100480. 10.1016/j.imr.2020.100480 32742920 PMC7366961

[B38] Lam C. L. Wong W. Fong D. Y. (2009). Chinese herbal medicine in the treatment of acute upper respiratory tract infection: a randomised, double blind, placebo-controlled clinical trial. Hong Kong Med. J. 15 (Suppl. 6), 30–34. 19801715

[B39] Lee H. Kang B. Hong M. Lee H. L. Choi J. Y. Lee J. A. (2020). Eunkyosan for the common cold: a PRISMA-Compliment systematic review of randomised, controlled trials. Med. Baltim. 99, e21415. 10.1097/MD.0000000000021415 32756141 PMC7402720

[B40] Lee B. Kwon C. Y. Suh H. W. Kim Y. J. Kim K. I. Lee B. J. (2023). Herbal medicine for the treatment of chronic cough: a systematic review and meta-analysis. Front. Pharmacol. 14, 1230604. 10.3389/fphar.2023.1230604 37920213 PMC10619915

[B41] Lei R. Shen Q. Yang B. Hou T. Liu H. Luo X. (2022). Core outcome sets in child health: a systematic review. JAMA Pediatr. 176, 1131–1141. 10.1001/jamapediatrics.2022.3181 36094597

[B42] Li Y. (2016a). Comparison of different Chinese and Western medicine treatment regimens for pediatric wind-heat type common cold. Inn. Mong. J. Chin. Med. 35, 49.

[B43] Li Z. Y. (2016b). Clinical observation on the efficacy of pediatric chai gui tui re granules combined with pediatric paracetamol and amantadine granules in treating children with common cold fever. China Pract. Med. 11, 207–208.

[B44] Li C. X. (2019). Clinical observation of miao medicine yunshi ganmao mixture combined with pediatric chai gui antipyretic granules in treating pediatric wind-cold type common cold. Health Must-Read 60.

[B45] Li H. J. (2020). Analysis of the therapeutic effect of Ge gen tang in clinical treatment of pediatric wind-cold type common cold. Health Preserv. Guide 33.

[B46] Li Y. (2021). Study on the application of the method of relieving the exterior and unblocking the bowels in pediatric common cold. Jinan, China: Shandong University of Traditional Chinese Medicine. Master’s Thesis.

[B47] Li S. H. Qian D. (2016). Efficacy of huangqi tiaoying tang in treating post-cold recurrent cough in children and its effect on immune indices IgA and IgE. Sichuan J. Traditional Chin. Med. 34, 75–77.

[B48] Li Z. W. Zhang Y. Gu G. X. (2014). Observation of 50 cases of pediatric acute upper respiratory infection with wind-heat syndrome treated by tui re oral liquid. J. Pract. Chin. Med. 30, 930–931.

[B49] Li G. Cai L. Jiang H. Dong S. Fan T. Liu W. (2015). Compound formulas of traditional Chinese medicine for the common cold: systematic review of randomized, placebo-controlled trials. Altern. Ther. Health Med. 21, 48–57. 26567449

[B50] Li G. Y. Liu Y. Q. Xie H. T. (2016). Therapeutic effect of pediatric dingchuan oral liquid on pediatric common cold. China Health Care Nutr. 26, 252.

[B51] Li H. Liang J. Li P. Li X. Liu Q. Yang S. (2024). Schizonepeta tenuifolia briq-saposhnikovia divaricata decoction alleviates atopic dermatitis *via* downregulating macrophage TRPV1. Front. Pharmacol. 15, 1413513. 10.3389/fphar.2024.1413513 39257398 PMC11383762

[B52] Liu Y. J. (2016). Clinical observation of huoxiang zhengqi capsules in the treatment of gastrointestinal-type common cold. Chin. Foreign Med. Care 35, 134–135.

[B53] Liu C. (2018). Observation on the efficacy of ganmao qingre granules in treating 160 cases of pediatric wind-cold type common cold. Electron. J. Mod. Med. Health Res. 2, 175.

[B54] Liu J. Lin H. J. (2019). Application of xiaokuihualu brand honeysuckle Dew combined with pediatric compound paracetamol and chlorphenamine granules in the treatment of pediatric common cold. Fam. Med. 180.

[B55] Llor C. Bjerrum L. (2014). Antimicrobial resistance: risk associated with antibiotic overuse and initiatives to reduce the problem. Ther. Adv. Drug Saf. 5, 229–241. 10.1177/2042098614554919 25436105 PMC4232501

[B56] Lu Q. Tang Z. H. Yang H. Huang S. L. Qian J. Yang F. (2024). Observation on clinical effect of self-made Ma gui chai Ge tang in treating pediatric common cold with fever. Chin. Foreign Med. Res. 3, 78–80.

[B57] Lucas S. Kumar S. Leach M. J. Phillips A. (2019). Parent use of complementary medicine remedies and services for the management of respiratory tract infection in children: a qualitative study. J. Multidiscip. Healthc. 12, 749–766. 10.2147/JMDH.S216687 31571893 PMC6750007

[B58] Luo G. D. (1996). Antiviral formula for the treatment of pediatric cold with fever. Hubei J. Traditional Chin. Med. 26.

[B59] Mao L. H. (2016). Observation on efficacy of Ge gen tang granules in treating pediatric wind-cold type common cold. People’s Mil. Surg. 59, 832–833.

[B60] Nahas R. Balla A. (2011). Complementary and alternative medicine for prevention and treatment of the common cold. Can. Fam. Physician 57, 31–36. 21322286 PMC3024156

[B61] Pachter L. M. Sumner T. Fontan A. Sneed M. Bernstein B. A. (1998). Home-based therapies for the common cold among European American and ethnic minority families: the interface between alternative/complementary and folk medicine. Archives Pediatr. and Adolesc. Med. 152, 1083–1088. 10.1001/archpedi.152.11.1083 9811285

[B62] Page M. J. Mckenzie J. E. Bossuyt P. M. Boutron I. Hoffmann T. C. Mulrow C. D. (2021). The PRISMA 2020 statement: an updated guideline for reporting systematic reviews. Bmj 372, n71. 10.1136/bmj.n71 33782057 PMC8005924

[B63] Peng D. H. Wang L. Y. (2016). Analysis of efficacy of ganmao qingre granules in the treatment of pediatric wind-cold type common cold. Mod. Distance Educ. Chin. Med. 14, 95–96.

[B64] Picca M. Carrozzo R. Milani G. P. Corsello A. Macchi M. Buzzetti R. (2023). Leading reasons for antibiotic prescriptions in pediatric respiratory infections: influence of fever in a primary care setting. Italian J. Pediatr. 49, 131. 10.1186/s13052-023-01533-5 37775784 PMC10541709

[B65] Qi W. B. (2016). Clinical observation on pediatric chai gui antipyretic granules combined with pediatric compound paracetamol and chlorphenamine granules in the treatment of pediatric cold with fever. Chin. Mod. Drug Appl. 10, 136–137.

[B66] Qian D. (2012). Efficacy analysis of lung-moistening, digestion-promoting, and phlegm-resolving Chinese medicine in treating pediatric cold with cough. Pract. J. Chin. Med. 26, 16–17.

[B67] Ran S. Peng R. Guo Q. Cui J. Chen G. Wang Z. (2024). Bupleurum in treatment of depression disorder: a comprehensive review. Pharm. (Basel) 17, 512. 10.3390/ph17040512 38675471 PMC11054835

[B68] Rivera D. Allkin R. ObóN C. Alcaraz F. Verpoorte R. Heinrich M. (2014). What is in a name? The need for accurate scientific nomenclature for plants. J. Ethnopharmacol. 152, 393–402. 10.1016/j.jep.2013.12.022 24374235

[B69] Saqib S. Ullah F. Naeem M. Younas M. Ayaz A. Ali S. (2022). Mentha: nutritional and health attributes to treat various ailments including cardiovascular diseases. Molecules 27, 6728. 10.3390/molecules27196728 36235263 PMC9572119

[B70] Seylanova N. Chernyavskaya A. Degtyareva N. Mursalova A. Ajam A. Xiao L. (2024). Core outcome measurement set for research and clinical practice in post-COVID-19 condition (long COVID) in children and young people: an international Delphi consensus study PC-COS children. Eur. Respir. J. 63, 2301761. 10.1183/13993003.01761-2023 38359962 PMC10938351

[B71] Shen Y. Z. (2013). Xiao chai Hu Tang in the treatment of 18 cases of pediatric common cold. Mod. Distance Educ. Chin. Med. 11, 103–104.

[B72] Shen N. N. Wu W. G. (2021). Observation on the efficacy of lianhua qingwen granules combined with ribavirin in 36 cases of phlegm-heat obstructing lung type viral common cold. Drug Eval. 18, 477–479.

[B73] Shi J. W. (2015). Clinical study on the treatment of 120 cases of pediatric common cold. Mother and Infant World. 2, 119.

[B74] Shi G. F. (2021). Clinical efficacy of modified xiao chai Hu tang in the treatment of pediatric common cold. Med. Aesthet. Cosmetol. 30, 135.

[B75] Shi L. T. Huang Y. F. Li Q. H. (2015). Clinical efficacy analysis of pediatric jiebiao oral liquid in 150 cases of pediatric wind-heat type common cold. North. Pharm. 12, 141–142.

[B76] Shin S. M. Shin J.-Y. Kim M. H. Lee S. H. Choi S. Park B.-J. (2015). Prevalence of antibiotic use for pediatric acute upper respiratory tract infections in Korea. J. Korean Med. Sci. 30, 617–624. 10.3346/jkms.2015.30.5.617 25931794 PMC4414647

[B77] Song B. (2017). Efficacy and safety of huoxiang zhengqi capsules in treating gastrointestinal type common cold. Inn. Mong. J. Chin. Med. 36, 22–23.

[B78] Sterne J. A. C. Savović J. Page M. J. Elbers R. G. Blencowe N. S. Boutron I. (2019). RoB 2: a revised tool for assessing risk of bias in randomised trials. BMJ 366, l4898. 10.1136/bmj.l4898 31462531

[B79] Summerlin J. Eiland L. S. (2025). The use and safety of cough and cold medications in the pediatric population. J. Pediatr. Pharmacol. Ther. 30, 17–26. 10.5863/1551-6776-30.1.17 39935563 PMC11809541

[B80] The Society of Internal Korean Medicine (2021). Korean medicine clinical practice guideline for common cold. Available online at: https://nikom.or.kr/board/boardFile/download/38/16521/21128.do (Accessed August 28, 2025).

[B81] Tran N. K. S. Lee J. H. Lee M. J. Park J. Y. Kang K. S. (2023). Multitargeted herbal prescription So shiho tang: a scoping review on biomarkers for the evaluation of therapeutic effects. Pharmaceuticals 16, 1371. 10.3390/ph16101371 37895842 PMC10610176

[B82] Turyasiima M. Kiconco G. Egesa W. I. Twesigemukama S. Nduwimana M. (2024). Prevalence and outpatient clinical diagnostic approaches for common acute respiratory tract infections in children under five years of age: a cross-sectional study. Pediatr. Health Med. Ther. 15, 49–57. 10.2147/PHMT.S445908 38268971 PMC10807262

[B83] Wang T. L. (2011). Clinical study on Chinese patent medicine regimen for pediatric wind-heat type common cold. *Master’s thesis* . Nanjing, China: Nanjing University of Chinese Medicine.

[B84] Wang L. Dong X. (2017). Effect of ganmao qingre granules on TCM syndrome scores in pediatric wind-cold type common cold. China Med. Equip. 32, 85.

[B85] Wang Z. Xia Q. Liu X. Liu W. Huang W. Mei X. (2018). Phytochemistry, pharmacology, quality control and future research of Forsythia suspensa (Thunb.) vahl: a review. J. Ethnopharmacol. 210, 318–339. 10.1016/j.jep.2017.08.040 28887216

[B86] Wen B. (2017). Clinical analysis of Modified Xiao Chai Hu Tang in the treatment of pediatric common cold. Everyone’s Health Late Ed. 11, 40.

[B87] Wong W. Lam C. L. Fong D. Y. (2012). Treatment effectiveness of two Chinese herbal medicine formulae in upper respiratory tract infections--a randomized double-blind placebo-controlled trial. Fam. Pract. 29, 643–652. 10.1093/fampra/cms027 22490614

[B88] Worrall G. (2011). Common cold. Can. Fam. Physician 57, 1289–1290. 22084460 PMC3215607

[B89] Wu Y. Y. (2010). Clinical comparative study of integrated Chinese and Western medicine treatment regimens for pediatric wind-heat type common cold. Master’s thesis. Nanjing, China: Nanjing University of Chinese Medicine.

[B90] Wu S. S. (2023). Clinical efficacy of Pediatric Chiqiao qingre granules in treating wind-heat type common cold with stagnation syndrome in children. Friend Health, 254–256.

[B91] Wu T. Zhang J. Qiu Y. Xie L. Liu G. J. (2007). Chinese medicinal herbs for the common cold. Cochrane Database Syst. Rev. 2007, CD004782. Cd004782. 10.1002/14651858.CD004782.pub2 17253524 PMC12547859

[B92] Xu H. Y. (2011). Clinical observation of 39 cases of pediatric common cold treated with xiao chai Hu tang. North. Pharm. 8, 94.

[B93] Xu Y. S. (2014). Clinical exploration of xiao chai Hu tang in the treatment of pediatric common cold. Med. Inf., 438.

[B94] Yan F. Q. Xu S. R. Shao M. G. Mou Y. F. (2021). Clinical evaluation of chai yin oral liquid in treating pediatric wind-heat type common cold. World Chin. Med. 16, 3653–3656.

[B95] Yang C. X. (2009). Clinical observation of 40 cases of pediatric common cold treated with jing Fang qingre san. Int. J. Traditional Chin. Med. 31, 427.

[B96] Yang H. (2010). Clinical observation of 30 cases of pediatric common cold treated with modified xiao chai Hu tang. Chin. J. Emerg. Traditional Chin. Med. 19, 207+213.

[B97] Yang Y. Y. (2016). Clinical observation on the efficacy of self-prepared pediatric Ganmao granules for pediatric wind-heat type common cold. Today’s Health 15, 314.

[B98] Yang S. (2017). Analysis of efficacy of Pediatric Feire Kechuan oral liquid as adjunctive therapy for pediatric wind-heat type common cold. Psychologist 23, 135–136.

[B99] Yang M. J. Wei Y. L. Hou Y. Y. Zhao J. H. (2015). Observation on the efficacy of Ganmao qingre granules in treating 37 cases of pediatric wind-cold type common cold. China Med. Guide 13, 192–193.

[B100] Yang Y. G. Wang X. Ma W. H. Zhang T. Wu W. G. Gao J. (2023). Clinical analysis of 68 cases of pediatric acute rhinitis treated with Ma Wenhong’s self-formulated shangfeng san with modifications. Inn. Mong. J. Chin. Med. 42, 8–10.

[B101] Ye W. W. (2016). Evaluative study on syndrome-differentiation application of Chinese patent medicine in treating pediatric wind-heat type common cold. Guoyi Forum 31, 26–28.

[B102] Zhang J. (2017). Clinical observation of 30 cases of pediatric gastrointestinal-type cold treated with weisukeli combined with pediatric compound aminopyrine and chlorphenamine granules. Fam. Med., 11–12.

[B103] Zhang Y. F. (2018a). Clinical study on jing Fang qing re san in treating pediatric common cold. Baojian Wenhui 268, 271.

[B104] Zhang Y. W. (2018b). Evaluation of the effect of modified xiao chai Hu tang in the treatment of pediatric common cold. Contemp. Med. Forum 16, 195–196.

[B105] Zhang Y. Qin S. (2022). Effect of Xiao Chai Hu Tang in treating pediatric common cold and relieving clinical symptoms. Women’s Health Research. 17, 80–81.

[B106] Zhang C. C. An C. X. Yang J. P. (2013). Clinical observation of 30 cases of pediatric wind-cold type common cold treated with Jing Fang Bai Du San with modifications. J. Community Med. 11, 38+43.

[B107] Zhang T. Wu Y. Guo Y. Yan B. Wei J. Zhang H. (2022). Risk of illness-related school absenteeism for elementary students with exposure to PM2.5 and O3. Sci. Total Environ. 842, 156824. 10.1016/j.scitotenv.2022.156824 35738367

[B108] Zhao Q. Chen X. Y. Martin C. (2016). Scutellaria baicalensis, the golden herb from the garden of Chinese medicinal plants. Sci. Bull. (Beijing) 61, 1391–1398. 10.1007/s11434-016-1136-5 27730005 PMC5031759

[B109] Zhou B. W. (2016). Clinical study of shanlameiye granules combined with ribavirin in treating children with acute upper respiratory tract infection. Mod. Drugs Clin. 31, 435–439.

[B110] Zhu Y. X. (2018). Study on YINLAI decoction with modifications in treating pediatric lung-stomach excess heat type common cold. Med. Front. 8, 319.

[B111] Zhu X. (2021). Clinical efficacy of xiao chai Hu tang in treating pediatric common cold. Inn. Mong. J. Traditional Chin. Med. 40, 69–70.

[B112] Zhu W. Wang L. J. (2023). Efficacy of xiao chai Hu san combined with pediatric Paracetamol Pseudoephedrine tablets in treating gastrointestinal type common cold in children and its effect on serum inflammatory factors. Chin. Health Preserv. 41, 28–31.

[B113] Zhu J. Lu Y. He Q. (2024). Recent advances on bioactive compounds, health benefits, and potential applications of jujube (Ziziphus jujuba mill.): a perspective of by-products valorization. Trends Food Sci. and Technol. 145, 104368. 10.1016/j.tifs.2024.104368

