# Supplementary material for: Pediatric efficacy and safety in common cold treated with herbal medicine (PEACH): a systematic review and meta-analysis
Source: Front Pharmacol. 2026 Jan 14;16:1703997. doi: 10.3389/fphar.2025.1703997 (PMC12847374; doi:10.3389/fphar.2025.1703997)
Supplement: Supplementary file 1 [file Table1.docx]

**Supplementary Table S1.** Search terms used in each database and the results of the search

| **PubMed** | **Search strategy** | **Results** |
| --- | --- | --- |
| **#1** | “Common Cold”[mh] OR Rhinovirus[mh] OR Rhinitis[mh] OR “Respiratory Tract Infections”[mh] OR nasopharyngitis[mh] OR "common cold*"[tiab] OR rhinovirus*[tiab] OR rhinitis[tiab] OR nasopharyngitis[tiab] OR rhinopharyngitis[tiab] OR coryza[tiab] OR catarrh[tiab] OR "upper respiratory infection*"[tiab] OR "upper respiratory tract infection*"[tiab] OR uri[tiab] OR urti[tiab] OR "upper airway infection*"[tiab] OR "cold virus*"[tiab] OR colds[tiab] | 719734 |
| **#2** | “Plants, Medicinal”[mh] OR “Drugs, Chinese Herbal”[mh] OR “Medicine, Chinese Traditional”[mh] OR “Medicine, Kampo”[mh] OR “Medicine, Korean Traditional”[mh] OR “Herbal Medicine”[mh] OR “Korean medicine”[tiab] OR “Chinese medicine”[tiab] OR TCM[tiab] OR “oriental medicine”[tiab] OR “traditional medicine”[tiab] OR “Kampo medicine”[tiab] OR “medicinal plants”[tiab] OR herb*[tiab] OR decoction[tiab] OR tang[tiab] OR capsule[tiab] OR powder[tiab] OR botanic*[tiab] | 460552 |
| **#3** | "randomized controlled trial"[pt] OR "controlled clinical trial"[pt] OR randomized[tiab] OR placebo[tiab] OR "clinical trials as topic"[mesh:noexp] OR randomly[tiab] OR trial[ti] | 1647571 |
| **#4** | animals[mh] NOT humans[mh] | 5265579 |
| **#5** | #1 AND #2 AND #3 NOT #4 | **1215** |
|  |  |  |
| **EMBASE** |  |  |
| **#1** | ('common cold'/exp OR ‘rhinovirus infection’/exp OR rhinitis/de OR ‘upper respiratory tract infection’/exp OR rhinopharyngitis/exp OR ‘common cold*’:ab,ti,kw OR rhinovirus*:ab,ti,kw OR rhinitis:ab,ti,kw OR nasopharyngitis:ab,ti,kw OR rhinopharyngitis:ab,ti,kw OR coryza:ab,ti,kw OR catarrh:ab,ti,kw OR ‘upper respiratory infection*’:ab,ti,kw OR ‘upper respiratory tract infection*’:ab,ti,kw OR uri:ab,ti,kw OR urti:ab,ti,kw OR ‘upper airway infection*’:ab,ti,kw OR ‘cold virus*’:ab,ti,kw OR colds:ab,ti,kw) | 192059 |
| **#2** | (‘medicinal plant’/exp OR ‘medicinal plant’:ab,ti,kw OR 'plant extract'/exp OR 'plant extract':ab,ti,kw OR ‘herbaceous agent’/exp OR ‘herbaceous agent’:ab,ti,kw OR ‘chinese medicine’/exp OR ‘chinese medicine’:ab,ti,kw OR TCM:ab,ti,kw OR ‘kampo medicine’/exp OR ‘kampo medicine’:ab,ti,kw OR ‘kampo medicine (drug)’/exp OR ‘korean medicine’/exp OR ‘korean medicine’:ab,ti,kw OR ‘herbal medicine’/exp OR 'traditional medicine'/exp OR 'traditional medicine':ab,ti,kw OR ‘oriental medicine’/exp OR ‘oriental medicine’:ab,ti,kw OR herb/exp OR herb*:ab,ti,kw OR decoction:ab,ti,kw OR tang:ab,ti,kw OR capsule:ab,ti,kw OR powder:ab,ti,kw OR botanic*:ab,ti,kw) | 949966 |
| **#3** | ('crossover procedure':de OR 'double-blind procedure':de OR 'randomized controlled trial':de OR 'single-blind procedure':de OR (random* OR factorial* OR crossover* OR (cross NEXT/1 over*) OR placebo* OR (doubl* NEAR/1 blind*) OR (singl* NEAR/1 blind*) OR assign* OR allocat* OR volunteer*):de,ab,ti) | 3412355 |
| **#4** | [animals]/lim NOT [humans]/lim | 6580838 |
| **#5** | #1 AND #2 AND #3 NOT #4 | **3030** |
|  |  |  |
| **CENTRAL** |  |  |
| **#1** | MeSH descriptor: [Common Cold] explode all trees | 663 |
| **#2** | MeSH descriptor: [Rhinovirus] explode all trees | 192 |
| **#3** | MeSH descriptor: [Rhinitis] explode all trees | 5145 |
| **#4** | MeSH descriptor: [Respiratory Tract Infections] explode all trees | 27936 |
| **#5** | MeSH descriptor: [Nasopharyngitis] in all MeSH products | 112 |
| **#6** | ("common cold" OR rhinovirus* OR rhinitis OR nasopharyngitis OR rhinopharyngitis OR coryza OR catarrh OR "upper respiratory infection" OR "upper respiratory tract infection" OR uri OR urti OR "upper airway infection" OR "cold virus" OR colds):ti,ab,kw | 21788 |
| **#7** | #1 OR #2 OR #3 OR #4 OR #5 OR #6 | 46411 |
| **#8** | MeSH descriptor: [Plants, Medicinal] explode all trees | 1149 |
| **#9** | MeSH descriptor: [Drugs, Chinese Herbal] explode all trees | 4696 |
| **#10** | MeSH descriptor: [Medicine, Chinese Traditional] explode all trees | 1806 |
| **#11** | MeSH descriptor: [Medicine, Kampo] explode all trees | 67 |
| **#12** | MeSH descriptor: [Medicine, Korean Traditional] explode all trees | 43 |
| **#13** | MeSH descriptor: [Herbal Medicine] in all MeSH products | 100 |
| **#14** | (“Korean medicine” OR “Chinese medicine” OR TCM OR “oriental medicine” OR “traditional medicine” OR “Kampo medicine” OR “medicinal plants” OR herb* OR decoction OR tang OR capsule OR powder OR botanic):ti,ab,kw | 72241 |
| **#15** | #8 OR #9 OR #10 OR #11 OR #12 OR #13 OR #14 | 73443 |
| **#16** | #7 AND #15 in Trials | **1929** |
|  |  |  |
| **CNKI** |  |  |
| #1 | ((TKA=‘感冒‘+‘伤风‘+‘普通感冒‘+‘外感发热‘+‘外感风寒‘+‘外感热病‘+‘外感高热‘+‘外感风热‘+‘急性鼻咽炎‘+‘急性上呼吸道感染‘) NOT (TI=‘流行性‘+‘流感‘+‘新冠肺炎‘+‘肺炎‘)) AND (TKA=‘草药’+’中医药’+’中医’+’中药’+’中成药’+’方剂’+’方药’+’本草’+’汤’+’丸’+’散’+’饮’+’颗粒’+’胶囊’+’口服液’) AND (TKA=‘随机‘+‘对照‘+‘随意‘+‘试验‘+‘安慰‘) NOT (TI=‘鼠‘+‘动物‘+‘meta‘+‘系统评价‘+‘回顾性‘+‘经验‘+‘近况‘+‘1则‘+‘一则‘+‘2则‘+‘总结‘+‘进展‘+‘发展‘+‘情况‘+‘概况‘+‘信息学‘+‘药效学‘+‘共识‘+‘指南‘) | 4222 |
| #2 | in Academic Journals, theses/Dissertations or Conferences | **3874** |
|  |  |  |
| **Wanfang** |  |  |
| #1 | ((((题名或关键词:(感冒 OR 伤风 OR 普通感冒 OR 外感发热 OR 外感风寒 OR 外感热病 OR 外感高热 OR 外感风热 OR 急性鼻咽炎 OR 急性上呼吸道感染) NOT 题名:(流行性 OR 流感 OR 新冠肺炎 OR 肺炎)) AND 题名或关键词:(草药 OR 中医药 OR 中医 OR 中药 OR 中成药 OR 方剂 OR 方药 OR 本草 OR 汤 OR 丸 OR 散 OR 饮 OR 颗粒 OR 胶囊 OR 口服液)) AND 全部:(随机 OR 对照 OR 随意 OR 试验 OR 安慰)) NOT 题名:(鼠 OR 动物 OR meta OR 系统评价 OR 回顾性 OR 经验 OR 近况 OR 1则 OR 一则 OR 2则 OR 总结 OR 进展 OR 发展 OR 情况 OR 概况 OR 信息学 OR 药效学 OR 共识 OR 指南)) | **2760** |
|  |  |  |
| **Cinii** |  |  |
| #1 | (風邪 OR 感冒 OR 急性鼻咽頭炎 OR 急性上気道感染症) AND (漢方薬 OR ハーブ OR 伝統医学 OR 漢方医学 OR 韓国医学 OR 東洋医学 OR 代替医療 OR 補完医学 OR 製剤 OR 剤 OR 中药 OR 湯 OR 丸 OR 散 OR 方 OR 顆粒) AND (ランダム化比較試験 OR 対照臨床試験 OR ランダム OR 無作為 OR 対照 OR 試験 OR 偽薬) | **117** |
|  |  |  |
| **OASIS** |  |  |
| #1 | (감기\|비인두염\|감모\|상기도감염) (한약\|약초\|본초\|방제\|탕\|환\|산) | **11** |
|  |  |  |
| **RISS** |  |  |
| #1 | 논문명:(감기\|비인두염\|감모\|상기도감염) AND 초록:(한약\|약초\|본초\|방제\|탕\|환\|산) | **61** |
|  |  |  |
| **ScienceOn** |  |  |
| #1 | 논문명:(감기\|비인두염\|감모\|상기도감염) AND 초록:(한약\|약초\|본초\|방제\|탕\|환\|산) | **3** |
