# Supplementary material for: Pediatric efficacy and safety in common cold treated with herbal medicine (PEACH): a systematic review and meta-analysis
Source: Front Pharmacol. 2026 Jan 14;16:1703997. doi: 10.3389/fphar.2025.1703997 (PMC12847374; doi:10.3389/fphar.2025.1703997)
Supplement: Supplementary file 2 [file Table2.docx]

**Supplementary Table S2**. Frequency of Individual Herbal Medicines Used in the Included Studies

| **No.** | **Herb (Scientific name)** | **Frequency n (%)** | **No.** | **Herb (Scientific name)** | **Frequency n (%)** |
| --- | --- | --- | --- | --- | --- |
| 1 | *Glycyrrhiza uralensis* (Licorice root) | 32 (47.1) | 31 | *Cicada slough* (*Cicadae Periostracum*) | 6 (8.8) |
| 2 | *Bupleurum chinense* (Chaihu) | 31 (45.6) | 32 | *Pogostemon cablin* (Patchouli) | 6 (8.8) |
| 3 | *Scutellaria baicalensis* (Baical skullcap root) | 21 (30.9) | 33 | *Paeonia lactiflora* (White peony root) | 6 (8.8) |
| 4 | *Schizonepeta tenuifolia* (Japanese catnip) | 17 (25.0) | 34 | *Peucedanum praeruptorum* (Qianhu root) | 5 (7.4) |
| 5 | *Forsythia suspensa* (Forsythia fruit) | 16 (23.5) | 35 | *Chrysanthemum morifolium* (Chrysanthemum flower) | 5 (7.4) |
| 6 | *Saposhnikovia divaricata* (Fangfeng root) | 15 (22.1) | 36 | *Spirodela polyrhiza* (Duckweed) | 5 (7.4) |
| 7 | *Prunus armeniaca* (Apricot seed) | 15 (22.1) | 37 | *Viola philippica* (Chinese violet) | 5 (7.4) |
| 8 | *Ziziphus jujuba* (Jujube fruit) | 14 (20.6) | 38 | *Magnolia officinalis* (Magnolia bark) | 4 (5.9) |
| 9 | *Mentha canadensis* (Field mint) | 14 (20.6) | 39 | *Arctium lappa* (Burdock fruit) | 4 (5.9) |
| 10 | *Platycodon grandiflorus* (Balloon flower root) | 14 (20.6) | 40 | *Lophatherum gracile* (Bamboo leaf) | 4 (5.9) |
| 11 | *Pueraria lobata* (Kudzu root) | 13 (19.1) | 41 | *Notopterygium incisum* (Qianghuo) | 3 (4.4) |
| 12 | *Citrus aurantium* (Bitter orange) | 12 (17.6) | 42 | *Ophiopogon japonicus* (Mai Dong) | 3 (4.4) |
| 13 | *Zingiber officinale* (Dried ginger, Ganjiang) | 12 (17.6) | 43 | *Angelica pubescens* (Duhuo) | 3 (4.4) |
| 14 | *Glycine max* (Fermented soybean, Semen Sojae Praeparatum) | 12 (17.6) | 44 | *Ligusticum chuanxiong* (Chuanxiong) | 3 (4.4) |
| 15 | *Pinellia ternata* (Pinellia rhizome) | 12 (17.6) | 45 | *Citrus reticulata* (Tangerine peel) | 3 (4.4) |
| 16 | *Lonicera japonica* (Honeysuckle flower) | 11 (16.2) | 46 | *Rheum palmatum* (Rhubarb root) | 3 (4.4) |
| 17 | *Artemisia annua* (Sweet wormwood) | 10 (14.7) | 47 | *Astragalus membranaceus* (Astragalus root) | 3 (4.4) |
| 18 | *Zingiber officinale* (Fresh ginger) | 9 (13.2) | 48 | *Areca catechu* (Betel nut) | 2 (2.9) |
| 19 | *Codonopsis pilosula* (Dangshen) | 9 (13.2) | 49 | *Lycium barbarum* (Goji berry root bark) | 2 (2.9) |
| 20 | *Isatis tinctoria* (Isatis root) | 8 (11.8) | 50 | *Crataegus pinnatifida* (Hawthorn fruit) | 2 (2.9) |
| 21 | *Quercus variabilis* (Gall, Galla Rhois) | 8 (11.8) | 51 | *Rehmannia glutinosa* (Rehmannia root) | 2 (2.9) |
| 22 | *Cinnamomum cassia* (Cinnamon twig) | 8 (11.8) | 52 | *Isatis tinctoria* (Isatis leaf) | 2 (2.9) |
| 23 | *Houttuynia cordata* (Houttuynia) | 8 (11.8) | 53 | *Cynanchum atratum* (Baiwei) | 2 (2.9) |
| 24 | *Phragmites communis* (Reed rhizome) | 7 (10.3) | 54 | *Gardenia jasminoides* (Gardenia fruit) | 2 (2.9) |
| 25 | *Poria cocos* (Poria) | 7 (10.3) | 55 | *Dryopteris crassirhizoma* (Male fern rhizome) | 2 (2.9) |
| 26 | *Ephedra sinica* (Ephedra herb) | 7 (10.3) | 56 | *Uncaria rhynchophylla* | 2 (2.9) |
| 27 | *Perilla frutescens* (Perilla leaf) | 7 (10.3) | 57 | *Bombyx mori* (Silkworm) | 2 (2.9) |
| 28 | *Gypsum fibrosum* (Gypsum) | 7 (10.3) | 58 | *Alisma plantago-aquatica* (Alisma) | 2 (2.9) |
| 29 | *Angelica dahurica* (Angelica root) | 6 (8.8) | 59 | *Morus alba* (Mulberry leaf) | 2 (2.9) |
| 30 | *Panax ginseng (Ginseng root)* | 6 (8.8) | 60 | *Pseudostellaria heterophylla* | 2 (2.9) |
