# Supplementary material for: Pediatric efficacy and safety in common cold treated with herbal medicine (PEACH): a systematic review and meta-analysis
Source: Front Pharmacol. 2026 Jan 14;16:1703997. doi: 10.3389/fphar.2025.1703997 (PMC12847374; doi:10.3389/fphar.2025.1703997)
Supplement: Supplementary file 3 [file DataSheet2.docx]

**Appendix 1**. Composition of Herbal Prescriptions

| **Study** | **Description** | **Composition** |
| --- | --- | --- |
| An 2012 | Jin Yu Chai Hu Tang | *Lonicera japonica* Thunb. [Caprifoliaceae; *Lonicerae flos*], *Houttuynia cordata* Thunb. [Saururaceae; *Houttuyniae herba*], *Bupleurum chinense* DC. [Apiaceae; *Bupleuri radix*], *Scutellaria baicalensis* Georgi [Lamiaceae; *Scutellariae radix*], *Gypsum fibrosum* [Mineral], *Codonopsis pilosula* (Franch.) Nannf. [Campanulaceae; *Codonopsis radix*], *Astragalus membranaceus* (Fisch.) Bunge [Fabaceae; *Astragali radix*], *Pinellia ternata* (Thunb.) Makino [Araceae; *Pinelliae tuber*], *Zingiber officinale* Roscoe [Zingiberaceae; *Zingiberis rhizoma*], *Ziziphus jujuba* Mill. [Rhamnaceae; *Ziziphi fructus*] |
| Bai 2019 | Jinyinhua Lu | *Lonicera japonica* Thunb. [Caprifoliaceae; *Lonicerae flos*] |
| Cai 2020 | Yunshi Ganmao Heji + Ganmao Jiedu granules | *Zingiber officinale* Roscoe [Zingiberaceae; *Zingiberis rhizoma*], *Caesalpinia decapetala* (Roth) Alston [Fabaceae], *Verbena officinalis* L. [Verbenaceae], *Clerodendrum cyrtophyllum* Turcz. [Lamiaceae] |
| Cao 2018 | Xiao Chai Hu Tang | NR |
| Ceng 2012 | Xiao Chai Hu Tang (M) | *Glycyrrhiza uralensis* Fisch. ex DC. [Fabaceae; *Glycyrrhizae radix*], *Scutellaria baicalensis* Georgi [Lamiaceae; *Scutellariae radix*], *Zingiber officinale* Roscoe [Zingiberaceae; *Zingiberis rhizoma*], *Bupleurum chinense* DC. [Apiaceae; *Bupleuri radix*], *Panax ginseng* C.A.Mey. [Araliaceae; *Ginseng radix*], *Ziziphus jujuba* Mill. [Rhamnaceae; *Ziziphi fructus*], *Pinellia ternata* (Thunb.) Makino [Araceae; *Pinelliae tuber*] |
| Chen 2015 | Yin Qiao San (M) | *Bupleurum chinense* DC. [Apiaceae; *Bupleuri radix*], *Glycine max* (L.) Merr. (fermented) [Fabaceae; *Sojae semen praeparatum*], *Codonopsis pilosula* (Franch.) Nannf. [Campanulaceae; *Codonopsis radix*], *Glycyrrhiza uralensis* Fisch. ex DC. [Fabaceae; *Glycyrrhizae radix*], *Zingiber officinale* Roscoe [Zingiberaceae; *Zingiberis rhizoma*], *Ziziphus jujuba* Mill. [Rhamnaceae; *Ziziphi fructus*], *Artemisia annua* L. [Asteraceae; *Artemisiae herba*], *Gallus gallus domesticus* [Phasianidae; Endothelium corneum], *Citrus aurantium* L. [Rutaceae; *Aurantii fructus*] |
| Cong 1998 | Da Huang Ma Xing Gan Cao Tang | *Glycyrrhiza uralensis* Fisch. ex DC. [Fabaceae; *Glycyrrhizae radix*], *Houttuynia cordata* Thunb. [Saururaceae; *Houttuyniae herba*], *Morus alba* L. (leaf) [Moraceae; *Mori folium*], *Saposhnikovia divaricata* (Turcz.) Schischk. [Apiaceae; *Saposhnikoviae radix*], *Prunus armeniaca* L. (seed) [Rosaceae; *Armeniacae semen*], *Phragmites communis* Trin. [Poaceae; *Phragmitis rhizoma*], *Platycodon grandiflorus* (Jacq.) A.DC. [Campanulaceae; *Platycodonis radix*], *Schizonepeta tenuifolia* Briq. [Lamiaceae; *Schizonepetae herba*], *Lophatherum gracile* Brongn. [Poaceae; *Lophatheri herba*], *Glycine max* (L.) Merr. (fermented) [Fabaceae; *Sojae semen praeparatum*], *Mentha canadensis* L. [Lamiaceae; *Menthae herba*], *Arctium lappa* L. [Asteraceae; *Arctii fructus*], *Forsythia suspensa* (Thunb.) Vahl [Oleaceae; *Forsythiae fructus*], *Lonicera japonica* Thunb. [Caprifoliaceae; *Lonicerae flos*] |
| Cui 2019 | Ge Gen Tang granules | *Rheum palmatum* L. [Polygonaceae; *Rhei radix et rhizoma*], *Ephedra sinica* Stapf [Ephedraceae; *Ephedrae herba*], *Glycyrrhiza uralensis* Fisch. ex DC. [Fabaceae; *Glycyrrhizae radix*], *Prunus armeniaca* L. (seed) [Rosaceae; *Armeniacae semen*] |
| Dai 2018 | Fuganlin oral liquid | *Pueraria lobata* (Willd.) Ohwi [Fabaceae; *Puerariae radix*], *Ephedra sinica* Stapf [Ephedraceae; *Ephedrae herba*], *Cinnamomum cassia* (L.) J.Presl [Lauraceae; *Cinnamomi cortex*], *Paeonia lactiflora* Pall. [Paeoniaceae; *Paeoniae radix alba*], *Zingiber officinale* Roscoe [Zingiberaceae; *Zingiberis rhizoma*], *Ziziphus jujuba* Mill. [Rhamnaceae; *Ziziphi fructus*], *Glycyrrhiza uralensis* Fisch. ex DC. [Fabaceae; *Glycyrrhizae radix*] |
| Dan 2021 | Pudilan Xiaoyan oral liquid | *Bidens pilosa* L. [Asteraceae; *Bidens herba*], *Chrysanthemum indicum* L. [Asteraceae; *Chrysanthemi indici flos*], *Astragalus membranaceus* (Fisch.) Bunge [Fabaceae; *Astragali radix*], *Panax quinquefolius* L. [Araliaceae; *Panacis quinquefolii radix*], *Isatis tinctoria* L. (root) [Brassicaceae; *Isatidis radix*], *Pogostemon cablin* (Blanco) Benth. [Lamiaceae; *Pogostemonis herba*], *Fritillaria thunbergii* Miq. [Liliaceae; *Fritillariae bulbus*], *Ephedra sinica* Stapf [Ephedraceae; *Ephedrae herba*], *Peucedanum praeruptorum* Dunn [Apiaceae; *Peucedani radix*], *Glycyrrhiza uralensis* Fisch. ex DC. [Fabaceae; *Glycyrrhizae radix*] |
| Di 2012 | Ganmao Qingre granules | *Taraxacum mongolicum* Hand.-Mazz. [Asteraceae; *Taraxaci herba*], *Viola philippica* Cav. [Violaceae; *Violae herba*], *Isatis tinctoria* L. (root) [Brassicaceae; *Isatidis radix*], *Scutellaria baicalensis* Georgi [Lamiaceae; *Scutellariae radix*] |
| Du 2014 | Xiao Chai Hu Tang | *Schizonepeta tenuifolia* Briq. [Lamiaceae; *Schizonepetae herba*], *Mentha canadensis* L. [Lamiaceae; *Menthae herba*], *Saposhnikovia divaricata* (Turcz.) Schischk. [Apiaceae; *Saposhnikoviae radix*], *Bupleurum chinense* DC. [Apiaceae; *Bupleuri radix*], *Perilla frutescens* (L.) Britton (leaf) [Lamiaceae; *Perillae folium*], *Pueraria lobata* (Willd.) Ohwi [Fabaceae; *Puerariae radix*], *Platycodon grandiflorus* (Jacq.) A.DC. [Campanulaceae; *Platycodonis radix*], *Prunus armeniaca* L. (seed) [Rosaceae; *Armeniacae semen*], *Angelica dahurica* (Hoffm.) Benth. & Hook.f. ex Franch. & Sav. [Apiaceae; *Angelicae dahuricae radix*], *Viola philippica* Cav. [Violaceae; *Violae herba*], *Phragmites communis* Trin. [Poaceae; *Phragmitis rhizoma*] |
| Duo 2017 | Qizhen Tangsan + Sanchen San | *Scutellaria baicalensis* Georgi [Lamiaceae; *Scutellariae radix*], *Panax ginseng* C.A.Mey. [Araliaceae; *Ginseng radix*], *Bupleurum chinense* DC. [Apiaceae; *Bupleuri radix*], *Glycyrrhiza uralensis* Fisch. ex DC. [Fabaceae; *Glycyrrhizae radix*], *Zingiber officinale* Roscoe [Zingiberaceae; *Zingiberis rhizoma*], *Pinellia ternata* (Thunb.) Makino [Araceae; *Pinelliae tuber*], *Ziziphus jujuba* Mill. [Rhamnaceae; *Ziziphi fructus*] |
| Gu 2018 | Xiao'er Chaigui Tuire granules | *Dalbergia odorifera* T.Chen [Fabaceae; *Dalbergiae lignum*], *Aquilaria sinensis* (Lour.) Gilg [Thymelaeaceae; *Aquilariae lignum resinatum*], *Sargentodoxa cuneata* (Oliv.) Rehder & E.H.Wilson [Lardizabalaceae; *Sargentodoxae caulis*], *Zingiber officinale* Roscoe [Zingiberaceae; *Zingiberis rhizoma*], *Terminalia chebula* Retz. [Combretaceae; *Chebulae fructus*], *Terminalia bellirica* (Gaertn.) Roxb. [Combretaceae; *Belliricae fructus*], *Phyllanthus emblica* L. [Phyllanthaceae; *Emblicae fructus*] |
| Guan 2017 | Xiao'er Chaigui Tuire granules | *Bovis calculus artifactus* [Animal], *Carthamus tinctorius* L. [Asteraceae; *Carthami flos*], *Citrus medica* L. var. *sarcodactylis* [Rutaceae; *Citri medicae sarcodactylis fructus*] |
| Han 2018 | Herbal Medicine | NR |
| Hu 2020 | Xingfang granules | *Schizonepeta tenuifolia* Briq. [Lamiaceae; *Schizonepetae herba*], *Saposhnikovia divaricata* (Turcz.) Schischk. [Apiaceae; *Saposhnikoviae radix*], *Notopterygium incisum* Ting ex H.T.Chang [Apiaceae; *Notopterygii rhizoma et radix*], *Angelica pubescens* Maxim.f. [Apiaceae; *Angelicae pubescentis radix*], *Bupleurum chinense* DC. [Apiaceae; *Bupleuri radix*], *Peucedanum praeruptorum* Dunn [Apiaceae; *Peucedani radix*], *Ligusticum chuanxiong* Hort. [Apiaceae; *Chuanxiong rhizoma*], *Citrus aurantium* L. [Rutaceae; *Aurantii fructus*], *Poria cocos* (Schw.) Wolf [Polyporaceae; *Poria*], *Platycodon grandiflorus* (Jacq.) A.DC. [Campanulaceae; *Platycodonis radix*], *Glycyrrhiza uralensis* Fisch. ex DC. [Fabaceae; *Glycyrrhizae radix*] |
| Hua 2017 | Jian'er Qingjie oral liquid | *Chrysanthemum morifolium* Ramat. [Asteraceae; *Chrysanthemi flos*], *Lonicera japonica* Thunb. [Caprifoliaceae; *Lonicerae flos*], *Forsythia suspensa* (Thunb.) Vahl [Oleaceae; *Forsythiae fructus*], *Prunus armeniaca* L. (seed) [Rosaceae; *Armeniacae semen*], *Citrus reticulata* Blanco (pericarp) [Rutaceae; *Citri reticulatae pericarpium*], *Crataegus pinnatifida* Bunge [Rosaceae; *Crataegi fructus*] |
| Ju 2012 | Wushicha granules OR Xiao'er Ganmao granules OR Xiao'er Ganmao Shu granules | NR |
| Li 2014 | Tuire oral liquid | *Indigo naturalis* [Mineralized indigo; *Indigo naturalis*], *Mentha canadensis* L. [Lamiaceae; *Menthae herba*], *Prunus armeniaca* L. (seed) [Rosaceae; *Armeniacae semen*], *Polygonum cuspidatum* Siebold & Zucc. [Polygonaceae; *Polygoni cuspidati rhizoma et radix*], *Artemisia annua* L. [Asteraceae; *Artemisiae herba*], *Gypsum fibrosum* [Mineral], *Forsythia suspensa* (Thunb.) Vahl [Oleaceae; *Forsythiae fructus*], *Uncaria rhynchophylla* (Miq.) Miq. ex Havil. [Rubiaceae; *Uncariae ramulus cum uncis*], *Eclipta prostrata* (L.) L. [Asteraceae; *Ecliptae herba*], *Dryopteris crassirhizoma* Nakai [Dryopteridaceae; *Dryopteridis rhizoma*], *Crataegus pinnatifida* Bunge [Rosaceae; *Crataegi fructus*], *Massa medicata fermentata* [Fermented product; *Massa medicata fermentata*] |
| Li 2016a | Xiao'er Dingchuan oral liquid | Composition not reported in original study |
| Li 2016b | Huangqi Diaoying Tang | *Astragalus membranaceus* (Fisch.) Bunge [Fabaceae; *Astragali radix*], *Pseudostellaria heterophylla* (Miq.) Pax [Caryophyllaceae; *Pseudostellariae radix*], *Salvia miltiorrhiza* Bunge [Lamiaceae; *Salviae miltiorrhizae radix et rhizoma*], *Poria cocos* (Schw.) Wolf [Polyporaceae; *Poria*], *Paeonia lactiflora* Pall. [Paeoniaceae; *Paeoniae radix alba*], *Scrophularia ningpoensis* Hemsl. [Scrophulariaceae; *Scrophulariae radix*], *Houttuynia cordata* Thunb. [Saururaceae; *Houttuyniae herba*], *Saposhnikovia divaricata* (Turcz.) Schischk. [Apiaceae; *Saposhnikoviae radix*], *Glycyrrhiza uralensis* Fisch. ex DC. [Fabaceae; *Glycyrrhizae radix*] |
| Li 2016c | Xiao'er Chaigui Tuire granules | *Bupleurum chinense* DC. [Apiaceae; *Bupleuri radix*], *Pueraria lobata* (Willd.) Ohwi [Fabaceae; *Puerariae radix*], *Cinnamomum cassia* (L.) J.Presl [Lauraceae; *Cinnamomi cortex*], *Spirodela polyrhiza* (L.) Schleid. [Araceae; *Spirodelae herba*], *Scutellaria baicalensis* Georgi [Lamiaceae; *Scutellariae radix*], *Paeonia lactiflora* Pall. [Paeoniaceae; *Paeoniae radix alba*], *Cryptotympana pustulata* Fabricius (slough) [Cicadidae; *Periostracum cicadae*] |
| Li 2016d | Yin Qiao San (M) | *Forsythia suspensa* (Thunb.) Vahl [Oleaceae; *Forsythiae fructus*], *Lonicera japonica* Thunb. [Caprifoliaceae; *Lonicerae flos*], *Prunus armeniaca* L. (seed) [Rosaceae; *Armeniacae semen*], *Houttuynia cordata* Thunb. [Saururaceae; *Houttuyniae herba*], *Saposhnikovia divaricata* (Turcz.) Schischk. [Apiaceae; *Saposhnikoviae radix*], *Mentha canadensis* L. [Lamiaceae; *Menthae herba*], *Arctium lappa* L. [Asteraceae; *Arctii fructus*], *Schizonepeta tenuifolia* Briq. [Lamiaceae; *Schizonepetae herba*], *Platycodon grandiflorus* (Jacq.) A.DC. [Campanulaceae; *Platycodonis radix*], *Lophatherum gracile* Brongn. [Poaceae; *Lophatheri herba*], *Glycine max* (L.) Merr. (fermented) [Fabaceae; *Sojae semen praeparatum*], *Morus alba* L. (leaf) [Moraceae; *Mori folium*], *Phragmites communis* Trin. [Poaceae; *Phragmitis rhizoma*], *Glycyrrhiza uralensis* Fisch. ex DC. [Fabaceae; *Glycyrrhizae radix*] |
| Li 2019 | Yunshi Ganmao Heji + Xiao'er Chaigui Tuire granules | *Caesalpinia decapetala* (Roth) Alston [Fabaceae], *Verbena officinalis* L. [Verbenaceae], *Clerodendrum cyrtophyllum* Turcz. [Lamiaceae], *Zingiber officinale* Roscoe [Zingiberaceae; *Zingiberis rhizoma*] |
| Li 2020 | Ge Gen Tang granules | *Cinnamomum cassia* (L.) J.Presl [Lauraceae; *Cinnamomi cortex*], *Pueraria lobata* (Willd.) Ohwi [Fabaceae; *Puerariae radix*], *Spirodela polyrhiza* (L.) Schleid. [Araceae; *Spirodelae herba*], *Scutellaria baicalensis* Georgi [Lamiaceae; *Scutellariae radix*] |
| Li 2021_1 | Xingfang Baidu San | Composition not reported in original study |
| Li 2021_2 | Yinqiao San | *Saposhnikovia divaricata* (Turcz.) Schischk. [Apiaceae; *Saposhnikoviae radix*], *Schizonepeta tenuifolia* Briq. [Lamiaceae; *Schizonepetae herba*], *Notopterygium incisum* Ting ex H.T.Chang [Apiaceae; *Notopterygii rhizoma et radix*], *Bupleurum chinense* DC. [Apiaceae; *Bupleuri radix*], *Poria cocos* (Schw.) Wolf [Polyporaceae; *Poria*], *Peucedanum praeruptorum* Dunn [Apiaceae; *Peucedani radix*], *Angelica pubescens* Maxim.f. [Apiaceae; *Angelicae pubescentis radix*], *Citrus aurantium* L. [Rutaceae; *Aurantii fructus*], *Ligusticum chuanxiong* Hort. [Apiaceae; *Chuanxiong rhizoma*], *Platycodon grandiflorus* (Jacq.) A.DC. [Campanulaceae; *Platycodonis radix*], *Glycyrrhiza uralensis* Fisch. ex DC. [Fabaceae; *Glycyrrhizae radix*] |
| Li 2021_3 | Sanren Tang | *Forsythia suspensa* (Thunb.) Vahl [Oleaceae; *Forsythiae fructus*], *Lonicera japonica* Thunb. [Caprifoliaceae; *Lonicerae flos*], *Schizonepeta tenuifolia* Briq. [Lamiaceae; *Schizonepetae herba*], *Lophatherum gracile* Brongn. [Poaceae; *Lophatheri herba*], *Platycodon grandiflorus* (Jacq.) A.DC. [Campanulaceae; *Platycodonis radix*], *Arctium lappa* L. [Asteraceae; *Arctii fructus*], *Glycine max* (L.) Merr. (fermented) [Fabaceae; *Sojae semen praeparatum*], *Mentha canadensis* L. [Lamiaceae; *Menthae herba*], *Phragmites communis* Trin. [Poaceae; *Phragmitis rhizoma*], *Glycyrrhiza uralensis* Fisch. ex DC. [Fabaceae; *Glycyrrhizae radix*] |
| Liu 2016 | Huoxiang Zhengqi capsule | *Prunus armeniaca* L. (seed) [Rosaceae; *Armeniacae semen*], *Coix lacryma-jobi* L. [Poaceae; *Coicis semen*], *Amygdalus communis* L. [Rosaceae; *Amygdalae semen*], *Bambusa tuldoides* Munro (leaf) [Poaceae; *Bambusae folium*], *Tetrapanax papyrifer* (Hook.) K.Koch (pith) [Araliaceae; *Medulla tetrapanacis*], *Magnolia officinalis* Rehder & E.H.Wilson [Magnoliaceae; *Magnoliae officinalis cortex*], *Pinellia ternata* (Thunb.) Makino [Araceae; *Pinelliae tuber*], *Talcum* [Mineral] |
| Liu 2018 | Ganmao Qingre granules | *Schizonepeta tenuifolia* Briq. [Lamiaceae; *Schizonepetae herba*], *Mentha canadensis* L. [Lamiaceae; *Menthae herba*], *Saposhnikovia divaricata* (Turcz.) Schischk. [Apiaceae; *Saposhnikoviae radix*], *Bupleurum chinense* DC. [Apiaceae; *Bupleuri radix*], *Perilla frutescens* (L.) Britton (leaf) [Lamiaceae; *Perillae folium*], *Pueraria lobata* (Willd.) Ohwi [Fabaceae; *Puerariae radix*], *Platycodon grandiflorus* (Jacq.) A.DC. [Campanulaceae; *Platycodonis radix*], *Prunus armeniaca* L. (seed) [Rosaceae; *Armeniacae semen*], *Angelica dahurica* (Hoffm.) Benth. & Hook.f. ex Franch. & Sav. [Apiaceae; *Angelicae dahuricae radix*], *Isatis indigotica* Fortune ex Lindl. (root) [Brassicaceae; *Isatidis radix*], *Phragmites communis* Trin. [Poaceae; *Phragmitis rhizoma*] |
| Liu 2019 | Jinyinhua Lu | *Lonicera japonica* Thunb. [Caprifoliaceae; *Lonicerae flos*] |
| Lu 2024 | Self-formulated Ma Gui Chai Ge Tang | *Ephedra sinica* Stapf [Ephedraceae; *Ephedrae herba*], *Cinnamomum cassia* (L.) J.Presl [Lauraceae; *Cinnamomi cortex*], *Platycodon grandiflorus* (Jacq.) A.DC. [Campanulaceae; *Platycodonis radix*], *Dryopteris crassirhizoma* Nakai [Dryopteridaceae; *Dryopteridis rhizoma*], *Paeonia lactiflora* Pall. [Paeoniaceae; *Paeoniae radix alba*], *Eupatorium fortunei* Turcz. [Asteraceae; *Eupatorii herba*], *Poria cocos* (Schw.) Wolf [Polyporaceae; *Poria*], *Alisma plantago-aquatica* L. [Alismataceae; *Alismatis rhizoma*], *Pueraria lobata* (Willd.) Ohwi [Fabaceae; *Puerariae radix*], *Zingiber officinale* Roscoe [Zingiberaceae; *Zingiberis rhizoma*], *Bupleurum chinense* DC. [Apiaceae; *Bupleuri radix*], *Glycyrrhiza uralensis* Fisch. ex DC. [Fabaceae; *Glycyrrhizae radix*], *Gypsum fibrosum* [Mineral], *Scutellaria baicalensis* Georgi [Lamiaceae; *Scutellariae radix*], *Pinellia ternata* (Thunb.) Makino [Araceae; *Pinelliae tuber*], *Citrus aurantium* L. [Rutaceae; *Aurantii fructus*], *Mentha canadensis* L. [Lamiaceae; *Menthae herba*] |
| Luo 1996 | Kangbingdu Fang | *Cinnamomum cassia* (L.) J.Presl [Lauraceae; *Cinnamomi cortex*], *Paeonia lactiflora* Pall. [Paeoniaceae; *Paeoniae radix alba*], *Bupleurum chinense* DC. [Apiaceae; *Bupleuri radix*], *Pueraria lobata* (Willd.) Ohwi [Fabaceae; *Puerariae radix*], *Schizonepeta tenuifolia* Briq. [Lamiaceae; *Schizonepetae herba*], *Saposhnikovia divaricata* (Turcz.) Schischk. [Apiaceae; *Saposhnikoviae radix*], *Perilla frutescens* (L.) Britton (leaf) [Lamiaceae; *Perillae folium*], *Glycyrrhiza uralensis* Fisch. ex DC. [Fabaceae; *Glycyrrhizae radix*] |
| Mao 2016 | Ge Gen Tang granules | *Pueraria lobata* (Willd.) Ohwi [Fabaceae; *Puerariae radix*], *Ephedra sinica* Stapf [Ephedraceae; *Ephedrae herba*], *Cinnamomum cassia* (L.) J.Presl [Lauraceae; *Cinnamomi cortex*], *Paeonia lactiflora* Pall. [Paeoniaceae; *Paeoniae radix alba*], *Zingiber officinale* Roscoe [Zingiberaceae; *Zingiberis rhizoma*], *Ziziphus jujuba* Mill. [Rhamnaceae; *Ziziphi fructus*], *Glycyrrhiza uralensis* Fisch. ex DC. [Fabaceae; *Glycyrrhizae radix*] |
| Peng 2016 | Ganmao Qingre granules | *Schizonepeta tenuifolia* Briq. [Lamiaceae; *Schizonepetae herba*], *Saposhnikovia divaricata* (Turcz.) Schischk. [Apiaceae; *Saposhnikoviae radix*], *Mentha canadensis* L. [Lamiaceae; *Menthae herba*], *Pueraria lobata* (Willd.) Ohwi [Fabaceae; *Puerariae radix*], *Bupleurum chinense* DC. [Apiaceae; *Bupleuri radix*], *Platycodon grandiflorus* (Jacq.) A.DC. [Campanulaceae; *Platycodonis radix*], *Angelica dahurica* (Hoffm.) Benth. & Hook.f. ex Franch. & Sav. [Apiaceae; *Angelicae dahuricae radix*], *Phragmites communis* Trin. [Poaceae; *Phragmitis rhizoma*], *Perilla frutescens* (L.) Britton (leaf) [Lamiaceae; *Perillae folium*], *Prunus armeniaca* L. (seed) [Rosaceae; *Armeniacae semen*], *Viola philippica* Cav. [Violaceae; *Violae herba*] |
| Qi 2016 | Xiao'er Chaigui Tuire granules | *Bupleurum chinense* DC. [Apiaceae; *Bupleuri radix*], *Cinnamomum cassia* (L.) J.Presl [Lauraceae; *Cinnamomi cortex*], *Pueraria lobata* (Willd.) Ohwi [Fabaceae; *Puerariae radix*], *Spirodela polyrhiza* (L.) Schleid. [Araceae; *Spirodelae herba*], *Scutellaria baicalensis* Georgi [Lamiaceae; *Scutellariae radix*], *Paeonia lactiflora* Pall. [Paeoniaceae; *Paeoniae radix alba*], *Cryptotympana pustulata* Fabricius (slough) [Cicadidae; *Periostracum cicadae*] |
| Qian 2012 | Runfei Xiaoshi Huatan | *Glycyrrhiza uralensis* Fisch. ex DC. [Fabaceae; *Glycyrrhizae radix*], *Hordeum vulgare* L. [Poaceae; *Hordei fructus germinatus*], *Oryza sativa* L. (fermented) [Poaceae; *Oryzae fructus germinatus*], *Prunus armeniaca* L. (seed) [Rosaceae; *Armeniacae semen*], *Trichosanthes kirilowii* Maxim. [Cucurbitaceae; *Trichosanthis fructus*], *Aster tataricus* L.f. [Asteraceae; *Asteris radix et rhizoma*], *Stemona japonica* (Blume) Miq. [Stemonaceae; *Stemonae radix*], *Ephedra sinica* Stapf [Ephedraceae; *Ephedrae herba*], *Tussilago farfara* L. [Asteraceae; *Farfarae flos*] |
| Shen 2013 | Xiao Chai Hu Tang | *Bupleurum chinense* DC. [Apiaceae; *Bupleuri radix*], *Scutellaria baicalensis* Georgi [Lamiaceae; *Scutellariae radix*], *Panax ginseng* C.A.Mey. [Araliaceae; *Ginseng radix*], *Glycyrrhiza uralensis* Fisch. ex DC. [Fabaceae; *Glycyrrhizae radix*], *Pinellia ternata* (Thunb.) Makino [Araceae; *Pinelliae tuber*], *Zingiber officinale* Roscoe [Zingiberaceae; *Zingiberis rhizoma*], *Ziziphus jujuba* Mill. [Rhamnaceae; *Ziziphi fructus*] |
| Shen 2021 | Lianhua Qingwen granules | *Isatis tinctoria* L. (root) [Brassicaceae; *Isatidis radix*], *Lonicera japonica* Thunb. [Caprifoliaceae; *Lonicerae flos*], *Forsythia suspensa* (Thunb.) Vahl [Oleaceae; *Forsythiae fructus*], *Houttuynia cordata* Thunb. [Saururaceae; *Houttuyniae herba*], *Dryopteris crassirhizoma* Nakai [Dryopteridaceae; *Dryopteridis rhizoma*], *Gypsum fibrosum* [Mineral], *Rhodiola crenulata* (Hook.f. & Thomson) H.Ohba [Crassulaceae; *Rhodiolae crenulatae radix et rhizoma*], *Pogostemon cablin* (Blanco) Benth. [Lamiaceae; *Pogostemonis herba*], *Ephedra sinica* Stapf [Ephedraceae; *Ephedrae herba*], *Glycyrrhiza uralensis* Fisch. ex DC. [Fabaceae; *Glycyrrhizae radix*], *Prunus armeniaca* L. (seed) [Rosaceae; *Armeniacae semen*], *Rheum palmatum* L. [Polygonaceae; *Rhei radix et rhizoma*], Menthol [Monoterpenoid; excipient] |
| Shi 2015a | Xiao Chai Hu Tang (M) | *Bupleurum chinense* DC. [Apiaceae; *Bupleuri radix*], *Glycine max* (L.) Merr. (fermented) [Fabaceae; *Sojae semen praeparatum*], *Codonopsis pilosula* (Franch.) Nannf. [Campanulaceae; *Codonopsis radix*], *Glycyrrhiza uralensis* Fisch. ex DC. [Fabaceae; *Glycyrrhizae radix*], *Zingiber officinale* Roscoe [Zingiberaceae; *Zingiberis rhizoma*], *Artemisia annua* L. [Asteraceae; *Artemisiae herba*], *Gallus gallus domesticus* (endothelium corneum) [Phasianidae; *Endothelium corneum gigeriae galli*], *Citrus aurantium* L. [Rutaceae; *Aurantii fructus*] |
| Shi 2015b | Xiao'er Jiebiao oral liquid | *Bos taurus* Linnaeus (bovis calculus artifactus) [Bovidae; *Bovis calculus artifactus*], *Lonicera japonica* Thunb. [Caprifoliaceae; *Lonicerae flos*], *Pueraria lobata* (Willd.) Ohwi [Fabaceae; *Puerariae radix*], *Forsythia suspensa* (Thunb.) Vahl [Oleaceae; *Forsythiae fructus*], *Scutellaria baicalensis* Georgi [Lamiaceae; *Scutellariae radix*], *Perilla frutescens* (L.) Britton (leaf) [Lamiaceae; *Perillae folium*], *Arctium lappa* L. [Asteraceae; *Arctii fructus*], *Saposhnikovia divaricata* (Turcz.) Schischk. [Apiaceae; *Saposhnikoviae radix*], *Schizonepeta tenuifolia* Briq. [Lamiaceae; *Schizonepetae herba*] |
| Shi 2021 | Xiao Chai Hu Tang (M) | *Bupleurum chinense* DC. [Apiaceae; *Bupleuri radix*], *Glycine max* (L.) Merr. (fermented) [Fabaceae; *Sojae semen praeparatum*], *Codonopsis pilosula* (Franch.) Nannf. [Campanulaceae; *Codonopsis radix*], *Glycyrrhiza uralensis* Fisch. ex DC. [Fabaceae; *Glycyrrhizae radix*], *Zingiber officinale* Roscoe [Zingiberaceae; *Zingiberis rhizoma*], *Artemisia annua* L. [Asteraceae; *Artemisiae herba*], *Citrus aurantium* L. [Rutaceae; *Aurantii fructus*], *Gallus gallus domesticus* (endothelium corneum) [Phasianidae; *Endothelium corneum gigeriae galli*] |
| Song 2017 | Huoxiang Zhengqi capsule | *Angelica dahurica* (Hoffm.) Benth. & Hook.f. ex Franch. & Sav. [Apiaceae; *Angelicae dahuricae radix*], *Atractylodes lancea* (Thunb.) DC. [Asteraceae; *Atractylodis rhizoma*], *Citrus reticulata* Blanco [Rutaceae; *Citri reticulatae pericarpium*], *Magnolia officinalis* Rehder & E.H.Wilson [Magnoliaceae; *Magnoliae officinalis cortex*], *Pinellia ternata* (Thunb.) Makino [Araceae; *Pinelliae tuber*], *Perilla frutescens* (L.) Britton (oil) [Lamiaceae; *Perillae oleum*], *Glycyrrhiza uralensis* Fisch. ex DC. [Fabaceae; *Glycyrrhizae radix*], *Areca catechu* L. [Arecaceae; *Arecae semen*], *Pogostemon cablin* (Blanco) Benth. [Lamiaceae; *Pogostemonis herba*], *Poria cocos* (Schw.) Wolf [Polyporaceae; *Poria*] |
| Wang 2011 | Xiao'er Ganmao granules | *Pogostemon cablin* (Blanco) Benth. [Lamiaceae; *Pogostemonis herba*], *Chrysanthemum morifolium* Ramat. [Asteraceae; *Chrysanthemi flos*], *Forsythia suspensa* (Thunb.) Vahl [Oleaceae; *Forsythiae fructus*], *Isatis tinctoria* L. (leaf) [Brassicaceae; *Isatidis folium*], *Isatis tinctoria* L. (root) [Brassicaceae; *Isatidis radix*], *Rehmannia glutinosa* (Gaertn.) DC. [Orobanchaceae; *Rehmanniae radix*], *Lycium barbarum* L. (root bark) [Solanaceae; *Lycii radicis cortex*], *Cynanchum atratum* Bunge [Apocynaceae; *Cynanchi atrati radix et rhizoma*], *Mentha canadensis* L. [Lamiaceae; *Menthae herba*], *Gypsum fibrosum* [Mineral] |
| Wang 2017 | Ganmao Qingre granules | *Schizonepeta tenuifolia* Briq. [Lamiaceae; *Schizonepetae herba*], *Bupleurum chinense* DC. [Apiaceae; *Bupleuri radix*], *Saposhnikovia divaricata* (Turcz.) Schischk. [Apiaceae; *Saposhnikoviae radix*], *Perilla frutescens* (L.) Britton (leaf) [Lamiaceae; *Perillae folium*], *Mentha canadensis* L. [Lamiaceae; *Menthae herba*], *Platycodon grandiflorus* (Jacq.) A.DC. [Campanulaceae; *Platycodonis radix*], *Pueraria lobata* (Willd.) Ohwi [Fabaceae; *Puerariae radix*], *Angelica dahurica* (Hoffm.) Benth. & Hook.f. ex Franch. & Sav. [Apiaceae; *Angelicae dahuricae radix*], *Phragmites communis* Trin. [Poaceae; *Phragmitis rhizoma*], *Prunus armeniaca* L. (seed) [Rosaceae; *Armeniacae semen*], *Viola philippica* Cav. [Violaceae; *Violae herba*] |
| Wen 2017 | Xiao Chai Hu Tang (M) | *Glycine max* (L.) Merr. (fermented) [Fabaceae; *Sojae semen praeparatum*], *Glycyrrhiza uralensis* Fisch. ex DC. [Fabaceae; *Glycyrrhizae radix*], *Zingiber officinale* Roscoe [Zingiberaceae; *Zingiberis rhizoma*], *Citrus aurantium* L. [Rutaceae; *Aurantii fructus*], *Bupleurum chinense* DC. [Apiaceae; *Bupleuri radix*], *Codonopsis pilosula* (Franch.) Nannf. [Campanulaceae; *Codonopsis radix*], *Ziziphus jujuba* Mill. [Rhamnaceae; *Ziziphi fructus*], *Artemisia annua* L. [Asteraceae; *Artemisiae herba*], *Gallus gallus domesticus* (endothelium corneum) [Phasianidae; *Endothelium corneum gigeriae galli*] |
| Wu 2010 | Xiao'er Ganmao granules | *Pogostemon cablin* (Blanco) Benth. [Lamiaceae; *Pogostemonis herba*], *Chrysanthemum morifolium* Ramat. [Asteraceae; *Chrysanthemi flos*], *Forsythia suspensa* (Thunb.) Vahl [Oleaceae; *Forsythiae fructus*], *Isatis tinctoria* L. (leaf) [Brassicaceae; *Isatidis folium*], *Isatis tinctoria* L. (root) [Brassicaceae; *Isatidis radix*], *Rehmannia glutinosa* (Gaertn.) DC. [Orobanchaceae; *Rehmanniae radix*], *Lycium barbarum* L. (root bark) [Solanaceae; *Lycii radicis cortex*], *Cynanchum atratum* Bunge [Apocynaceae; *Cynanchi atrati radix et rhizoma*], *Mentha canadensis* L. [Lamiaceae; *Menthae herba*], *Gypsum fibrosum* [Mineral] |
| Wu 2023 | Children's Chiqiao Qingre granules | *Mentha canadensis* L. [Lamiaceae; *Menthae herba*], *Forsythia suspensa* (Thunb.) Vahl [Oleaceae; *Forsythiae fructus*], *Schizonepeta tenuifolia* Briq. [Lamiaceae; *Schizonepetae herba*], *Rheum palmatum* L. [Polygonaceae; *Rhei radix et rhizoma*], *Glycine max* (L.) Merr. (fermented) [Fabaceae; *Sojae semen praeparatum*], *Artemisia annua* L. [Asteraceae; *Artemisiae herba*], *Gardenia jasminoides* J.Ellis [Rubiaceae; *Gardeniae fructus*], *Areca catechu* L. [Arecaceae; *Arecae semen*], *Paeonia lactiflora* Pall. (red) [Paeoniaceae; *Paeoniae radix rubra*], *Magnolia officinalis* Rehder & E.H.Wilson [Magnoliaceae; *Magnoliae officinalis cortex*], *Pinellia ternata* (Thunb.) Makino [Araceae; *Pinelliae tuber*], *Scutellaria baicalensis* Georgi [Lamiaceae; *Scutellariae radix*], *Glycyrrhiza uralensis* Fisch. ex DC. [Fabaceae; *Glycyrrhizae radix*], *Bupleurum chinense* DC. [Apiaceae; *Bupleuri radix*] |
| Xu 2011 | Xiao Chai Hu Tang (M) | *Bupleurum chinense* DC. [Apiaceae; *Bupleuri radix*], *Glycine max* (L.) Merr. (fermented) [Fabaceae; *Sojae semen praeparatum*], *Codonopsis pilosula* (Franch.) Nannf. [Campanulaceae; *Codonopsis radix*], *Glycyrrhiza uralensis* Fisch. ex DC. [Fabaceae; *Glycyrrhizae radix*], *Ziziphus jujuba* Mill. [Rhamnaceae; *Ziziphi fructus*], *Zingiber officinale* Roscoe [Zingiberaceae; *Zingiberis rhizoma*], *Artemisia annua* L. [Asteraceae; *Artemisiae herba*], *Citrus aurantium* L. [Rutaceae; *Aurantii fructus*], *Gallus gallus domesticus* (endothelium corneum) [Phasianidae; *Endothelium corneum gigeriae galli*] |
| Xu 2014 | Xiao Chai Hu Tang | *Ziziphus jujuba* Mill. [Rhamnaceae; *Ziziphi fructus*], *Pinellia ternata* (Thunb.) Makino [Araceae; *Pinelliae tuber*], *Panax ginseng* C.A.Mey. [Araliaceae; *Ginseng radix*], *Zingiber officinale* Roscoe [Zingiberaceae; *Zingiberis rhizoma*], *Scutellaria baicalensis* Georgi [Lamiaceae; *Scutellariae radix*], *Glycyrrhiza uralensis* Fisch. ex DC. [Fabaceae; *Glycyrrhizae radix*], *Bupleurum chinense* DC. [Apiaceae; *Bupleuri radix*] |
| Yan 2021 | Chaiyin oral liquid | *Bupleurum chinense* DC. [Apiaceae; *Bupleuri radix*], *Lonicera japonica* Thunb. [Caprifoliaceae; *Lonicerae flos*], *Scutellaria baicalensis* Georgi [Lamiaceae; *Scutellariae radix*], *Pueraria lobata* (Willd.) Ohwi [Fabaceae; *Puerariae radix*], *Artemisia annua* L. [Asteraceae; *Artemisiae herba*], *Schizonepeta tenuifolia* Briq. [Lamiaceae; *Schizonepetae herba*], *Forsythia suspensa* (Thunb.) Vahl [Oleaceae; *Forsythiae fructus*], *Platycodon grandiflorus* (Jacq.) A.DC. [Campanulaceae; *Platycodonis radix*], *Prunus armeniaca* L. (seed) [Rosaceae; *Armeniacae semen*], *Mentha canadensis* L. [Lamiaceae; *Menthae herba*], *Houttuynia cordata* Thunb. [Saururaceae; *Houttuyniae herba*] |
| Yang 2009 | Xingfang Qingre San | *Schizonepeta tenuifolia* Briq. [Lamiaceae; *Schizonepetae herba*], *Saposhnikovia divaricata* (Turcz.) Schischk. [Apiaceae; *Saposhnikoviae radix*], *Forsythia suspensa* (Thunb.) Vahl [Oleaceae; *Forsythiae fructus*], *Cryptotympana pustulata* Fabricius (slough) [Cicadidae; *Periostracum cicadae*], *Uncaria rhynchophylla* (Miq.) Miq. ex Havil. [Rubiaceae; *Uncariae ramulus cum uncis*], *Ophiopogon japonicus* (L.f.) Ker Gawl. [Asparagaceae; *Ophiopogonis radix*], *Bombyx mori* L. (stiff silkworm) [Bombycidae; *Bombyx batryticatus*], *Prunus armeniaca* L. (seed) [Rosaceae; *Armeniacae semen*], *Platycodon grandiflorus* (Jacq.) A.DC. [Campanulaceae; *Platycodonis radix*] |
| Yang 2010 | Xiao Chai Hu Tang (M) | *Bupleurum chinense* DC. [Apiaceae; *Bupleuri radix*], *Glycine max* (L.) Merr. (fermented) [Fabaceae; *Sojae semen praeparatum*], *Codonopsis pilosula* (Franch.) Nannf. [Campanulaceae; *Codonopsis radix*], *Glycyrrhiza uralensis* Fisch. ex DC. [Fabaceae; *Glycyrrhizae radix*], *Ziziphus jujuba* Mill. [Rhamnaceae; *Ziziphi fructus*], *Zingiber officinale* Roscoe [Zingiberaceae; *Zingiberis rhizoma*], *Artemisia annua* L. [Asteraceae; *Artemisiae herba*], *Citrus aurantium* L. [Rutaceae; *Aurantii fructus*], *Gallus gallus domesticus* (endothelium corneum) [Phasianidae; *Endothelium corneum gigeriae galli*] |
| Yang 2015 | Ganmao Qingre granules | *Schizonepeta tenuifolia* Briq. [Lamiaceae; *Schizonepetae herba*], *Mentha canadensis* L. [Lamiaceae; *Menthae herba*], *Saposhnikovia divaricata* (Turcz.) Schischk. [Apiaceae; *Saposhnikoviae radix*], *Bupleurum chinense* DC. [Apiaceae; *Bupleuri radix*], *Perilla frutescens* (L.) Britton (leaf) [Lamiaceae; *Perillae folium*], *Pueraria lobata* (Willd.) Ohwi [Fabaceae; *Puerariae radix*], *Platycodon grandiflorus* (Jacq.) A.DC. [Campanulaceae; *Platycodonis radix*], *Prunus armeniaca* L. (seed) [Rosaceae; *Armeniacae semen*], *Angelica dahurica* (Hoffm.) Benth. & Hook.f. ex Franch. & Sav. [Apiaceae; *Angelicae dahuricae radix*], *Viola philippica* Cav. [Violaceae; *Violae herba*], *Phragmites communis* Trin. [Poaceae; *Phragmitis rhizoma*] |
| Yang 2016 | Xiao'er Ganmao granules | *Chrysanthemum morifolium* Ramat. [Asteraceae; *Chrysanthemi flos*], *Forsythia suspensa* (Thunb.) Vahl [Oleaceae; *Forsythiae fructus*], *Isatis tinctoria* L. [Brassicaceae; *Isatidis folium / radix*], *Gypsum fibrosum* [Mineral], *Pogostemon cablin* (Blanco) Benth. [Lamiaceae; *Pogostemonis herba*] |
| Yang 2017 | Xiaoer Feire Kechuan oral liquid | *Prunus armeniaca* L. (seed) [Rosaceae; *Armeniacae semen*], *Ephedra sinica* Stapf [Ephedraceae; *Ephedrae herba*], *Gypsum fibrosum* [Mineral], *Lonicera japonica* Thunb. [Caprifoliaceae; *Lonicerae flos*], *Scutellaria baicalensis* Georgi [Lamiaceae; *Scutellariae radix*], *Glycyrrhiza uralensis* Fisch. ex DC. [Fabaceae; *Glycyrrhizae radix*], *Houttuynia cordata* Thunb. [Saururaceae; *Houttuyniae herba*], *Ophiopogon japonicus* (L.f.) Ker Gawl. [Asparagaceae; *Ophiopogonis radix*], *Anemarrhena asphodeloides* Bunge [Asparagaceae; *Anemarrhenae rhizoma*], *Isatis tinctoria* L. [Brassicaceae; *Isatidis folium / radix*], *Forsythia suspensa* (Thunb.) Vahl [Oleaceae; *Forsythiae fructus*] |
| Yang 2023 | Shang Feng San (M) | *Glycine max* (L.) Merr. (fermented) [Fabaceae; *Sojae semen praeparatum*], *Schizonepeta tenuifolia* Briq. [Lamiaceae; *Schizonepetae herba*], *Saposhnikovia divaricata* (Turcz.) Schischk. [Apiaceae; *Saposhnikoviae radix*], *Magnolia liliflora* Desr. (bud) [Magnoliaceae; *Magnoliae liliflorae flos*], *Xanthium strumarium* L. [Asteraceae; *Xanthii fructus*], *Angelica dahurica* (Hoffm.) Benth. & Hook.f. ex Franch. & Sav. [Apiaceae; *Angelicae dahuricae radix*], *Euphorbia humifusa* Willd. [Euphorbiaceae; *Euphorbiae humifusae herba*], *Prunus armeniaca* L. (seed) [Rosaceae; *Armeniacae semen*], *Platycodon grandiflorus* (Jacq.) A.DC. [Campanulaceae; *Platycodonis radix*] |
| Ye 2016 | Lanqin oral liquid | *Isatis tinctoria* L. [Brassicaceae; *Isatidis folium / radix*], *Gardenia jasminoides* J.Ellis [Rubiaceae; *Gardeniae fructus*], *Scutellaria baicalensis* Georgi [Lamiaceae; *Scutellariae radix*], *Phellodendron amurense* Rupr. [Rutaceae; *Phellodendri amurensis cortex*], *Sterculia lychnophora* Hance [Sterculiaceae; *Sterculiae lychnophorae semen*] |
| Zhang 2013 | Xingfang Baidu San (M) | *Schizonepeta tenuifolia* Briq. [Lamiaceae; *Schizonepetae herba*], *Saposhnikovia divaricata* (Turcz.) Schischk. [Apiaceae; *Saposhnikoviae radix*], *Notopterygium incisum* Ting ex H.T.Chang [Apiaceae; *Notopterygii rhizoma et radix*], *Angelica pubescens* Maxim.f. [Apiaceae; *Angelicae pubescentis radix*], *Ligusticum chuanxiong* Hort. [Apiaceae; *Chuanxiong rhizoma*], *Bupleurum chinense* DC. [Apiaceae; *Bupleuri radix*], *Citrus aurantium* L. [Rutaceae; *Aurantii fructus*], *Platycodon grandiflorus* (Jacq.) A.DC. [Campanulaceae; *Platycodonis radix*], *Peucedanum praeruptorum* Dunn [Apiaceae; *Peucedani radix*], *Poria cocos* (Schw.) Wolf [Polyporaceae; *Poria*], *Panax ginseng* C.A.Mey. [Araliaceae; *Ginseng radix*], *Mentha canadensis* L. [Lamiaceae; *Menthae herba*], *Glycyrrhiza uralensis* Fisch. ex DC. [Fabaceae; *Glycyrrhizae radix*], *Zingiber officinale* Roscoe [Zingiberaceae; *Zingiberis rhizoma*] |
| Zhang 2017 | Weisu granules | *Cyperus rotundus* L. [Cyperaceae; *Cyperi rhizoma*], *Perilla frutescens* (L.) Britton (stem) [Lamiaceae; *Perillae caulis*], *Citrus reticulata* Blanco [Rutaceae; *Citri reticulatae pericarpium*], *Citrus medica* L. var. *sarcodactylis* [Rutaceae; *Citri medicae sarcodactylis fructus*], *Citrus aurantium* L. (fruit) [Rutaceae; *Aurantii fructus*], *Areca catechu* L. [Arecaceae; *Arecae semen*], *Gallus gallus domesticus* (endothelium corneum) [Phasianidae; *Endothelium corneum gigeriae galli*] |
| Zhang 2018a | Xiao Chai Hu Tang (M) | *Bupleurum chinense* DC. [Apiaceae; *Bupleuri radix*], *Citrus aurantium* L. [Rutaceae; *Aurantii fructus*], *Artemisia annua* L. [Asteraceae; *Artemisiae herba*], *Codonopsis pilosula* (Franch.) Nannf. [Campanulaceae; *Codonopsis radix*], *Glycine max* (L.) Merr. (fermented) [Fabaceae; *Sojae semen praeparatum*], *Gallus gallus domesticus* (endothelium corneum) [Phasianidae; *Endothelium corneum gigeriae galli*], *Zingiber officinale* Roscoe [Zingiberaceae; *Zingiberis rhizoma*], *Ziziphus jujuba* Mill. [Rhamnaceae; *Ziziphi fructus*], *Glycyrrhiza uralensis* Fisch. ex DC. [Fabaceae; *Glycyrrhizae radix*] |
| Zhang 2018b | Xingfang Qingre San | *Schizonepeta tenuifolia* Briq. [Lamiaceae; *Schizonepetae herba*], *Saposhnikovia divaricata* (Turcz.) Schischk. [Apiaceae; *Saposhnikoviae radix*], *Forsythia suspensa* (Thunb.) Vahl [Oleaceae; *Forsythiae fructus*], *Cryptotympana pustulata* Fabricius (slough) [Cicadidae; *Periostracum cicadae*], *Uncaria rhynchophylla* (Miq.) Miq. ex Havil. [Rubiaceae; *Uncariae ramulus cum uncis*], *Ophiopogon japonicus* (L.f.) Ker Gawl. [Asparagaceae; *Ophiopogonis radix*], *Bombyx mori* L. (stiff silkworm) [Bombycidae; *Bombyx batryticatus*], *Prunus armeniaca* L. (seed) [Rosaceae; *Armeniacae semen*], *Platycodon grandiflorus* (Jacq.) A.DC. [Campanulaceae; *Platycodonis radix*] |
| Zhang 2022 | Xiao Chai Hu Tang | *Bupleurum chinense* DC. [Apiaceae; *Bupleuri radix*], *Ziziphus jujuba* Mill. [Rhamnaceae; *Ziziphi fructus*], *Pseudostellaria heterophylla* (Miq.) Pax [Caryophyllaceae; *Pseudostellariae radix*], *Zingiber officinale* Roscoe [Zingiberaceae; *Zingiberis rhizoma*], *Alisma plantago-aquatica* L. [Alismataceae; *Alismatis rhizoma*], *Glycyrrhiza uralensis* Fisch. ex DC. [Fabaceae; *Glycyrrhizae radix*], *Scutellaria baicalensis* Georgi [Lamiaceae; *Scutellariae radix*] |
| Zhou 2016 | Shanlameiye granules | *Chimonanthus praecox* (L.) Link (leaf) [Calycanthaceae; Chimonanthus leaf (*Chimonanthi folium*; not pharmacopeial-standard)] |
| Zhu 2018 | Yinlai Tang | *Lonicera japonica* Thunb. [Caprifoliaceae; *Lonicerae flos*], *Raphanus sativus* L. [Brassicaceae; *Raphani semen*], *Forsythia suspensa* (Thunb.) Vahl [Oleaceae; *Forsythiae fructus*], *Scutellaria baicalensis* Georgi [Lamiaceae; *Scutellariae radix*], *Peucedanum praeruptorum* Dunn [Apiaceae; *Peucedani radix*], *Trichosanthes kirilowii* Maxim. [Cucurbitaceae; *Trichosanthis fructus*], *Houttuynia cordata* Thunb. [Saururaceae; *Houttuyniae herba*] |
| Zhu 2021 | Xiao Chai Hu Tang | *Bupleurum chinense* DC. [Apiaceae; *Bupleuri radix*], *Scutellaria baicalensis* Georgi [Lamiaceae; *Scutellariae radix*], *Zingiber officinale* Roscoe [Zingiberaceae; *Zingiberis rhizoma*], *Panax ginseng* C.A.Mey. [Araliaceae; *Ginseng radix*], *Glycyrrhiza uralensis* Fisch. ex DC. [Fabaceae; *Glycyrrhizae radix*], *Ziziphus jujuba* Mill. [Rhamnaceae; *Ziziphi fructus*], *Pinellia ternata* (Thunb.) Makino [Araceae; *Pinelliae tuber*] |
| Zhu 2023 | Xiao Chai Hu granules | *Codonopsis pilosula* (Franch.) Nannf. [Campanulaceae; *Codonopsis radix*], *Zingiber officinale* Roscoe [Zingiberaceae; *Zingiberis rhizoma*], *Bupleurum chinense* DC. [Apiaceae; *Bupleuri radix*], *Scutellaria baicalensis* Georgi [Lamiaceae; *Scutellariae radix*], *Pinellia ternata* (Thunb.) Makino [Araceae; *Pinelliae tuber*], *Glycyrrhiza uralensis* Fisch. ex DC. [Fabaceae; *Glycyrrhizae radix*], *Ziziphus jujuba* Mill. [Rhamnaceae; *Ziziphi fructus*], *Saccharum officinarum* L. (sugarcane; sweetening agent) [Poaceae; *Sacchari granulum*] |

M, modified prescription; NR, not reported
