# Supplementary material for: Pediatric efficacy and safety in common cold treated with herbal medicine (PEACH): a systematic review and meta-analysis
Source: Front Pharmacol. 2026 Jan 14;16:1703997. doi: 10.3389/fphar.2025.1703997 (PMC12847374; doi:10.3389/fphar.2025.1703997)
Supplement: Supplementary file 4 [file DataSheet1.DOCX]

**Supplementary Figures**


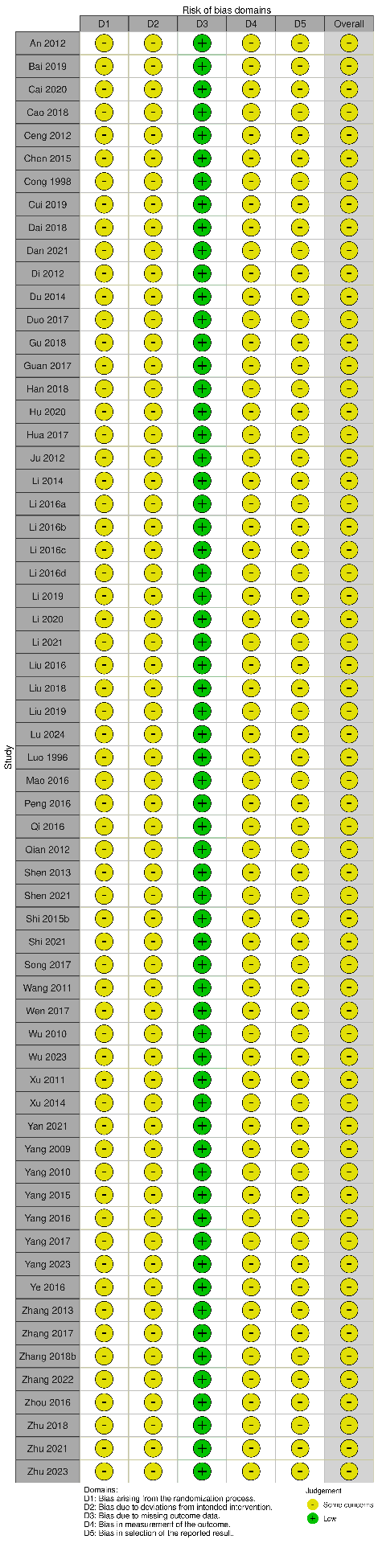


**Supplementary Figure S1a.** Risk of bias assessment for total effect rate


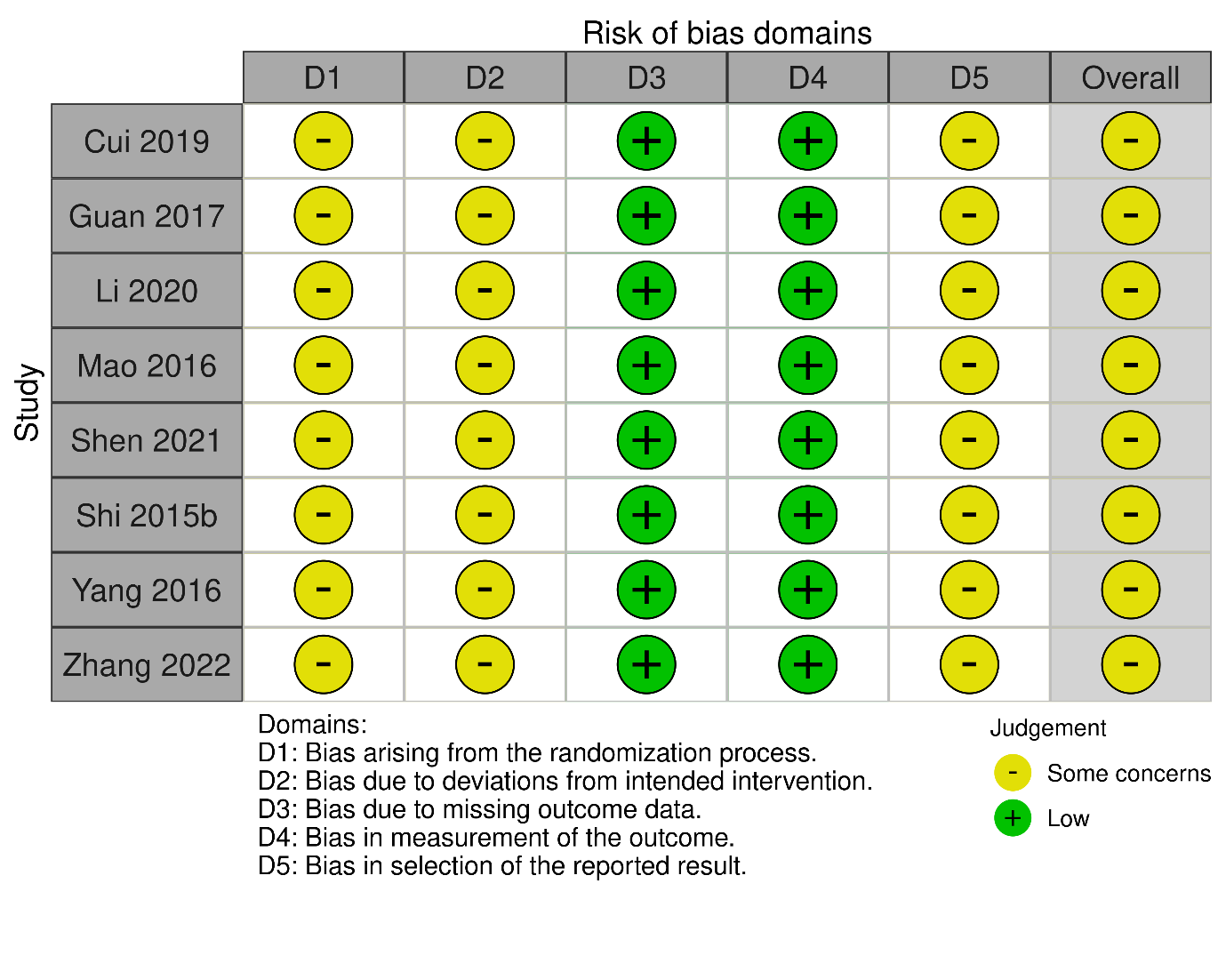


**Supplementary Figure S1b.** Risk of bias assessment for total symptom improvement time.


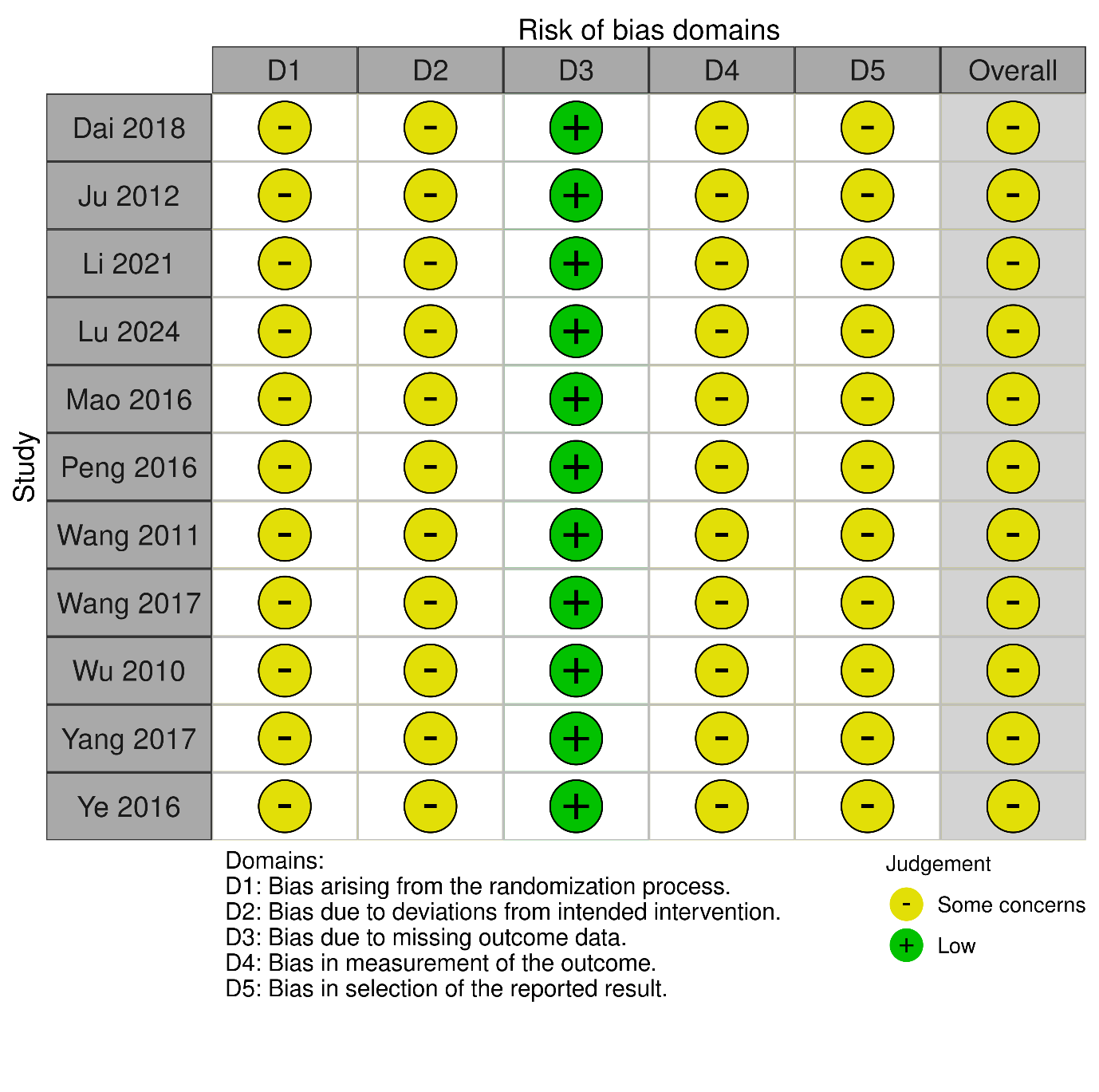


**Supplementary Figure S1c.** Risk of bias assessment for total symptom severity


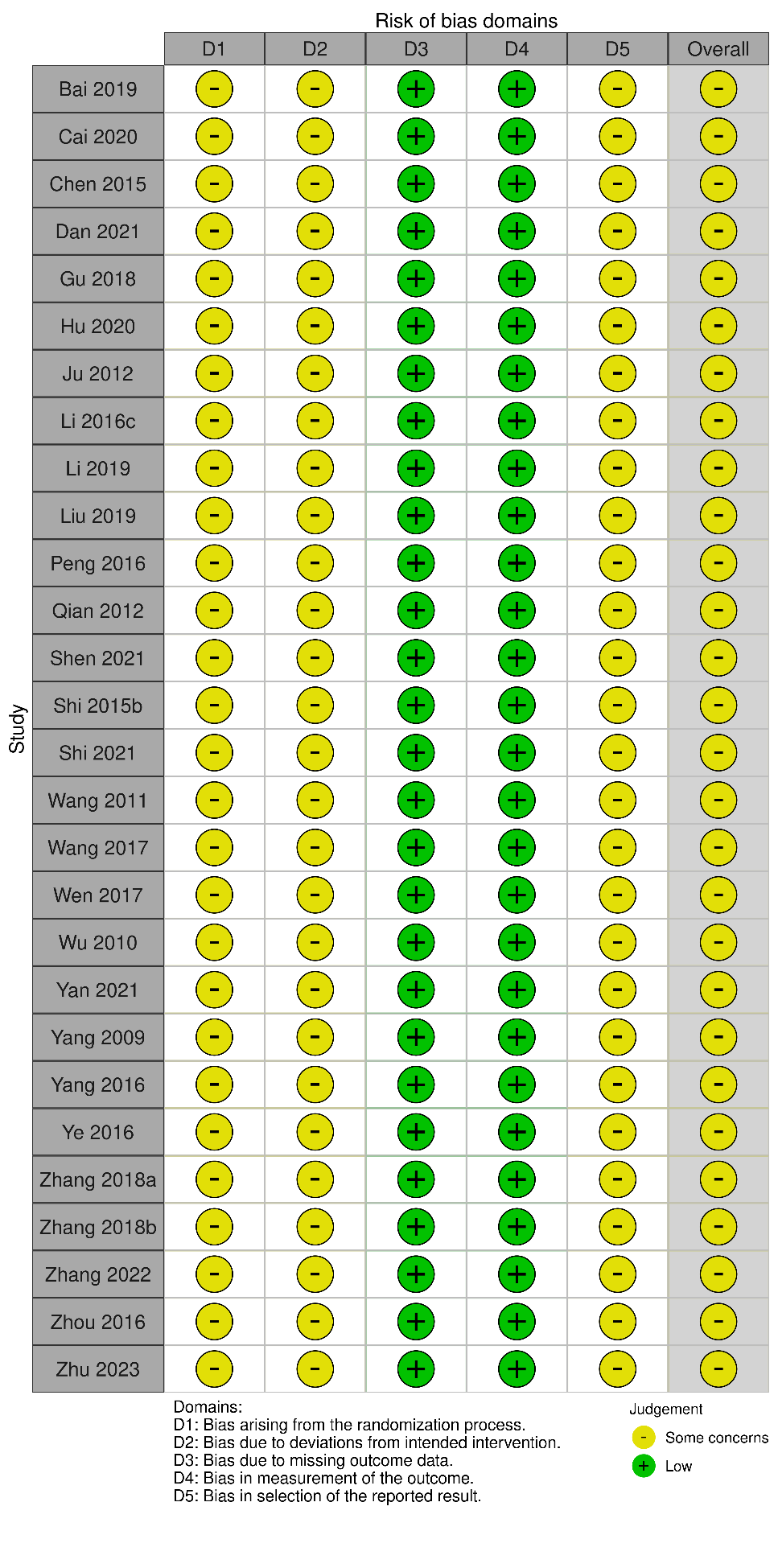


**Supplementary Figure S1d.** Risk of bias assessment for individual symptom improvement time

**
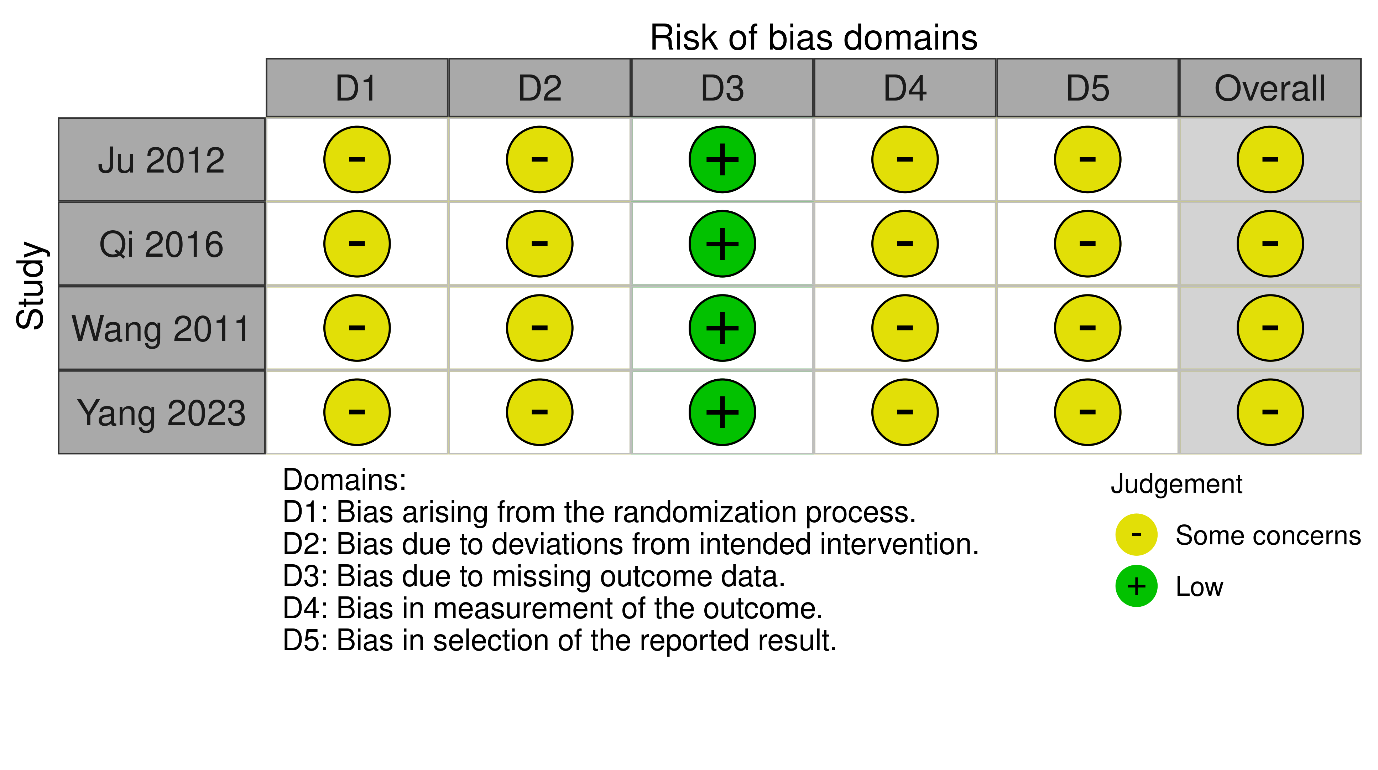
**

**Supplementary Figure S1e.** Risk of bias assessment for individual symptom severity


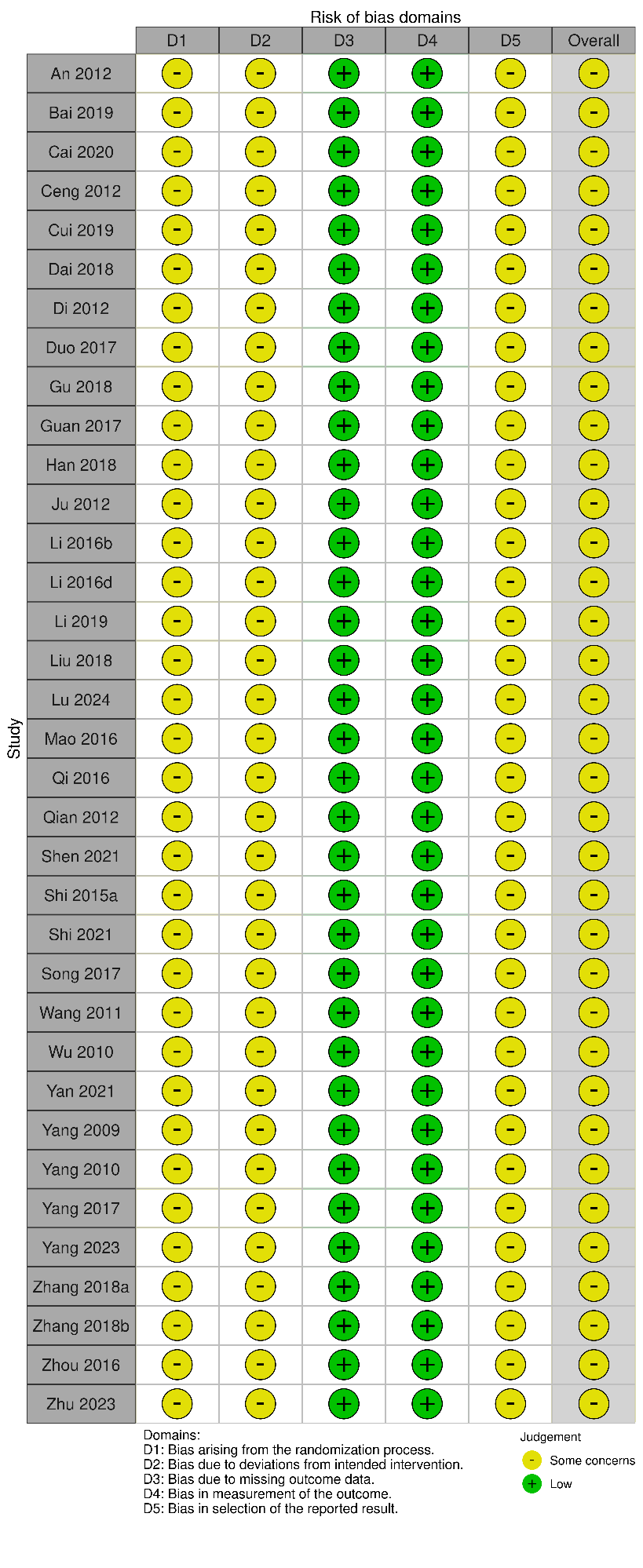


**Supplementary Figure S1f.** Risk of bias assessment for adverse events


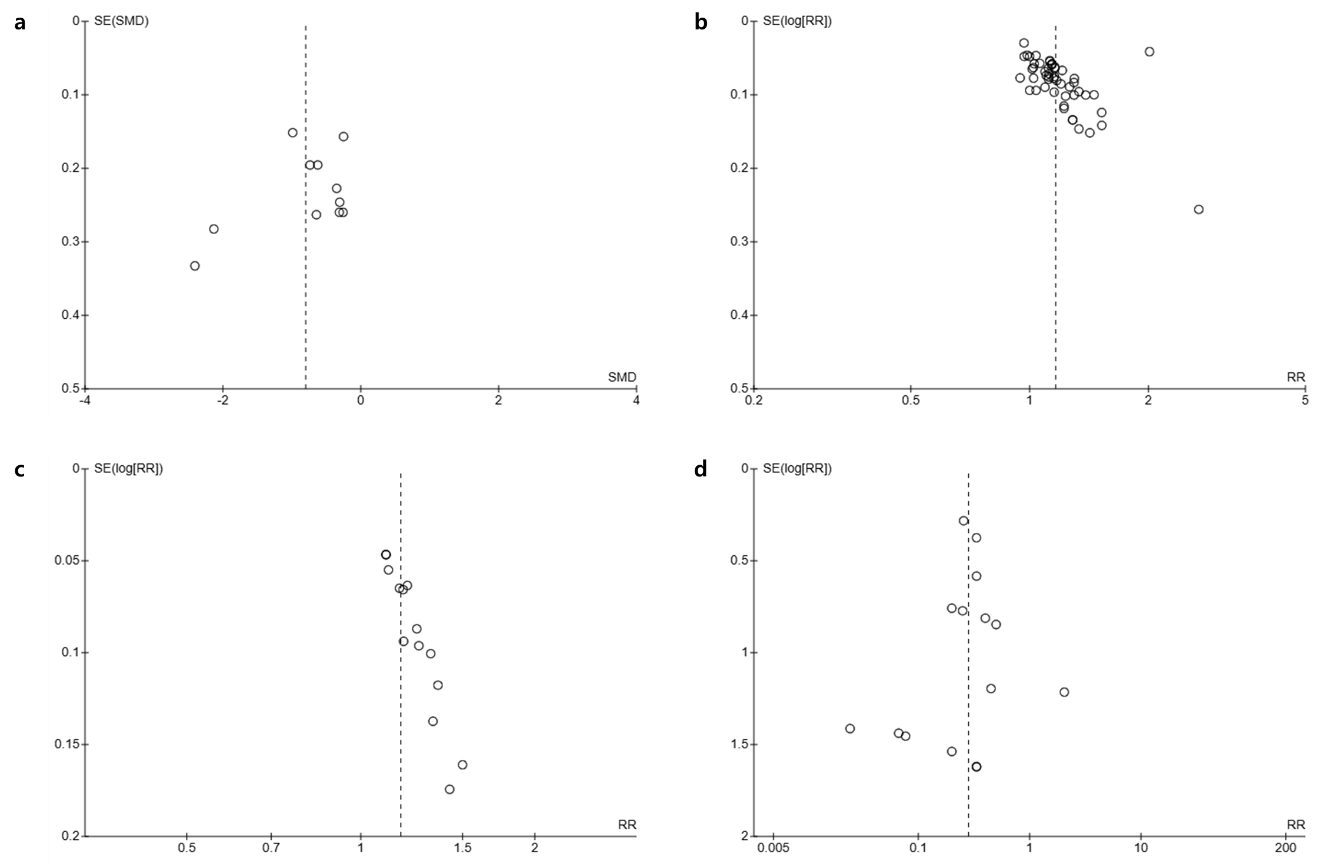


**Supplementary Figure S2.** Funnel plots for publication bias assessment: (a) Total symptom severity of HM vs. Conventional medicine; (b) Total effective rate of HM vs. Conventional medicine; (c) Total effective rate of HM + control vs. Conventional medicine; (d) Adverse events of HM vs. Conventional medicine. SE, standard error of log risk ratio; RR, risk ratio.


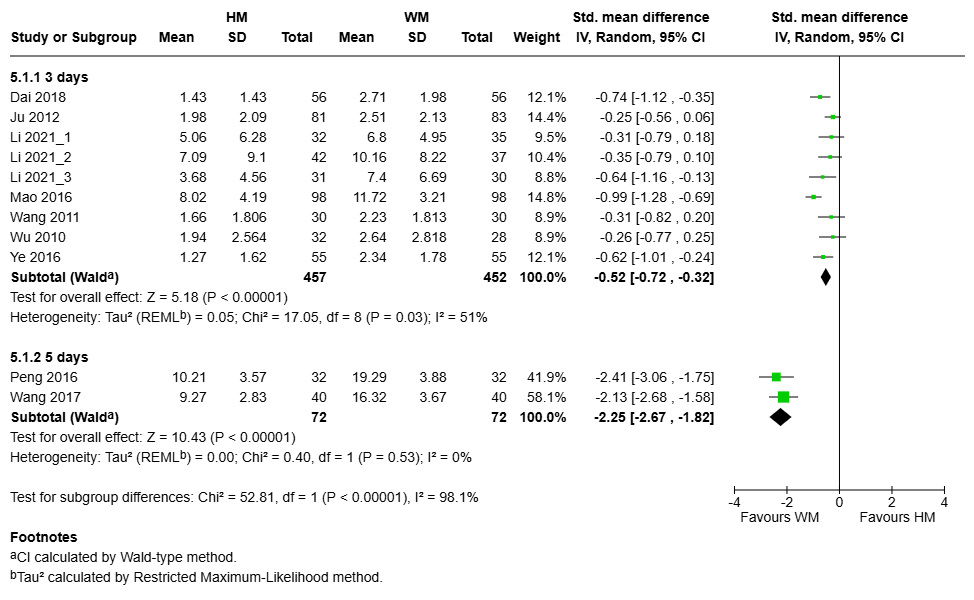


**Supplementary Figure S3.** Subgroup analysis of total symptom severity by treatment duration.

HM, herbal medicine; WM, Western medicine; SD, standard deviation; SMD, standardized mean difference; CI, confidence interval.


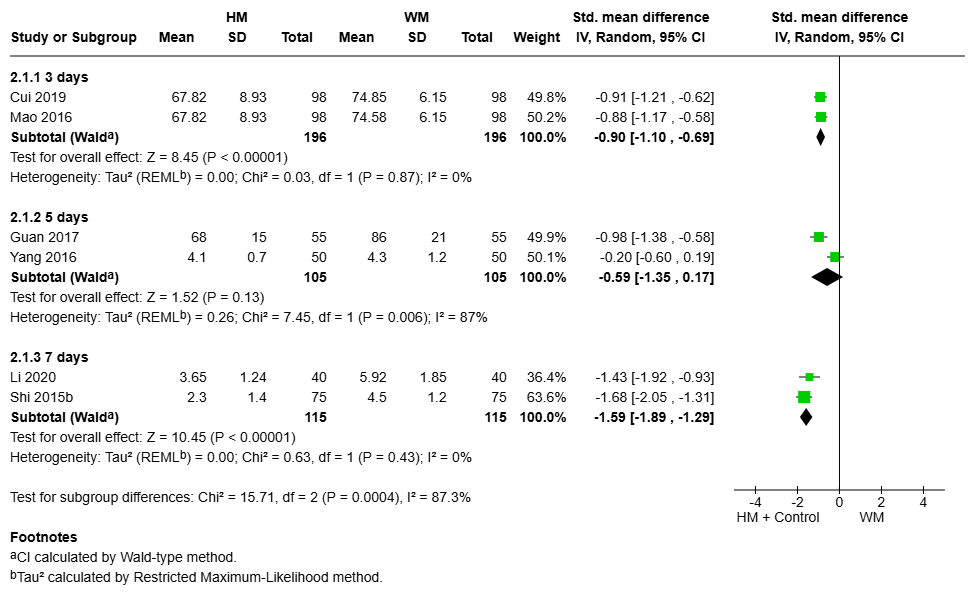


**Supplementary Figure S4.** Subgroup analysis of symptom improvement time by treatment duration.

HM, herbal medicine; WM, Western medicine; SD, standard deviation; SMD, standardized mean difference; CI, confidence interval.


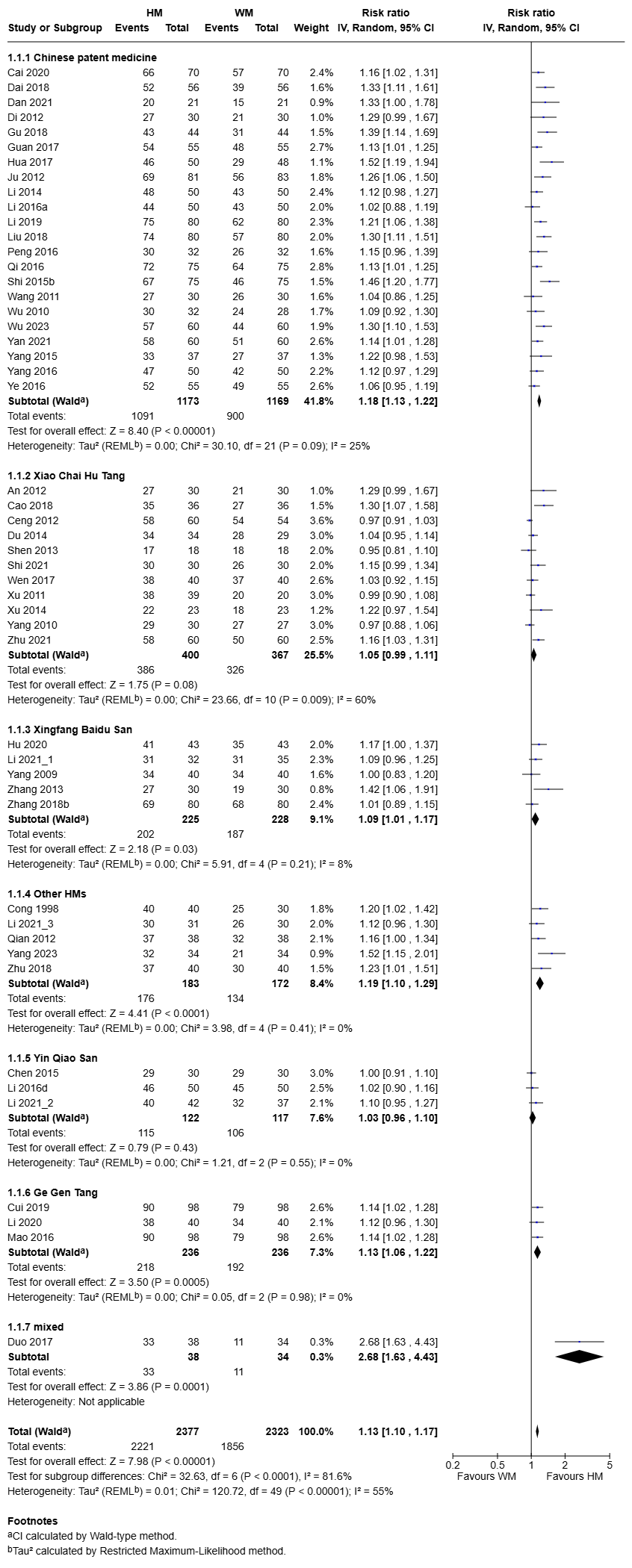


**Supplementary Figure S5.** Subgroup analysis of total effective rate by intervention type.

HM, herbal medicine; WM, Western medicine; SD, standard deviation; CI, confidence interval.
